# Reciprocal Effects on Neurocognitive and Metabolic Phenotypes in Mouse Models of 16p11.2 Deletion and Duplication Syndromes

**DOI:** 10.1371/journal.pgen.1005709

**Published:** 2016-02-12

**Authors:** Thomas Arbogast, Abdel-Mouttalib Ouagazzal, Claire Chevalier, Maksym Kopanitsa, Nurudeen Afinowi, Eugenia Migliavacca, Belinda S. Cowling, Marie-Christine Birling, Marie-France Champy, Alexandre Reymond, Yann Herault

**Affiliations:** 1 Institut de Génétique et de Biologie Moléculaire et Cellulaire, Illkirch, France; 2 Centre National de la Recherche Scientifique, UMR7104, Illkirch, France; 3 Institut National de la Santé et de la Recherche Médicale, U964, Illkirch, France; 4 Université de Strasbourg, Illkirch, France; 5 Synome Ltd, Moneta Building, Babraham Research Campus, Cambridge, United Kingdom; 6 Center for Integrative Genomics, University of Lausanne, Lausanne, Switzerland; 7 Swiss Institute of Bioinformatics (SIB), Lausanne, Switzerland; 8 PHENOMIN, Institut Clinique de la Souris, ICS; CNRS, INSERM, University of Strasbourg, Illkirch-Graffenstaden, France; Stanford University School of Medicine, UNITED STATES

## Abstract

The 16p11.2 600 kb BP4-BP5 deletion and duplication syndromes have been associated with developmental delay; autism spectrum disorders; and reciprocal effects on the body mass index, head circumference and brain volumes. Here, we explored these relationships using novel engineered mouse models carrying a deletion (*Del/+*) or a duplication (*Dup/+*) of the *Sult1a1-Spn* region homologous to the human 16p11.2 BP4-BP5 locus. On a C57BL/6N inbred genetic background, *Del/+* mice exhibited reduced weight and impaired adipogenesis, hyperactivity, repetitive behaviors, and recognition memory deficits. In contrast, *Dup/+* mice showed largely opposite phenotypes. On a F1 C57BL/6N × C3B hybrid genetic background, we also observed alterations in social interaction in the *Del/+* and the *Dup/+* animals, with other robust phenotypes affecting recognition memory and weight. To explore the dosage effect of the 16p11.2 genes on metabolism, *Del/+* and *Dup/+* models were challenged with high fat and high sugar diet, which revealed opposite energy imbalance. Transcriptomic analysis revealed that the majority of the genes located in the *Sult1a1-Spn* region were sensitive to dosage with a major effect on several pathways associated with neurocognitive and metabolic phenotypes. Whereas the behavioral consequence of the 16p11 region genetic dosage was similar in mice and humans with activity and memory alterations, the metabolic defects were opposite: adult *Del/+* mice are lean in comparison to the human obese phenotype and the *Dup/+* mice are overweight in comparison to the human underweight phenotype. Together, these data indicate that the dosage imbalance at the 16p11.2 locus perturbs the expression of modifiers outside the CNV that can modulate the penetrance, expressivity and direction of effects in both humans and mice.

## Author Summary

The 16p11.2 BP4-BP5 deletion and duplication syndromes are frequent copy number variants in humans, and are associated with developmental delay and autism spectrum disorders, with a reciprocal effect on head circumference and body mass index. Here we explored gene dosage effect in mouse models and found that the deletion and duplication induced opposite behavioral phenotypes. Notably, we observed that some behavioral phenotypes, such as social interaction, were sensitive to the genetic background. For the metabolism, the energy imbalance and adipocyte phenotypes were mirrored in the deletion and duplication carriers but opposite to the human phenotypes, the deletion mouse carriers were lean whereas the individuals with the deletion were obese. The main cause of the phenotypic features is the copy number variation of the 16p11.2 region with many genetic pathways altered in the striatum and the liver. Thus the final consequences of the rearrangement are likely governed by the interplay between many cellular pathways in both human cases and mouse models.

## Introduction

Understanding the interactions between genes that determine brain activity, metabolism and behavior is crucial to decipher changes in underlying neurocognitive disorders. For syndromes caused by copy number variation at the 16p11.2 locus, causal gene discovery is particularly challenging. The region shows a high density of genes, the majority of which are expressed in the brain and are potentially important for not only the normal development of the nervous system but they also seem to impact body mass index. The 16p11.2 600 kb BP4-BP5 breakpoint (BP) deletions and reciprocal duplications both have a population prevalence of approximately 1/1000 [1], and both genetic lesions are found in almost 1% of all cases with intellectual disability (ID)[2] and autism spectrum disorders (ASD) [3–7]. Phonological processing and language disorders are affected in 56% (deletion) and 46% (duplication) of the 16p11.2 patients [7] connected with brain anatomic alterations of the auditory and language systems [8], but no hearing impairment has been reported in these individuals so far [7, 9]. In addition to ID and ASD, both rearrangements have also been associated with epilepsy [10–13], whereas the duplication has been linked to schizophrenia, bipolar disorder and depression [14–16]. Opposite effects of 16p11.2 deletions and duplications on body mass index (BMI) and head size have also been reported in 16p11.2 CNVs carriers [1, 11, 12, 17, 18]. We showed recently that the brain volume and the specific cortico-striatal structures were similarly correlated with the number of copies of the 600 kb region [19].

The BP4-BP5 recurrent rearrangements contain 28 “unique” genes (*SPN*, *QPRT*, *C16orf54*, *ZG16*, *KIF22*, *MAZ*, *PRRT2*, *PAGR1* (*C16orf53*), *MVP*, *CDIPT*, *CDIPT-AS*, *SEZ6L2*, ASPHD1, *KCTD13*, *TMEM219*, *TAOK2*, *HIRIP3*, *INO80E*, *DOC2A*, *C16orf92*, *FAM57B*, *ALDOA*, *PP4C*, *TBX6*, *YPEL3*, *GDPD3*, *MAPK3*, *CORO1A*) and multiple copies of *BOLA2*/*2B*, *SLX1A*/*1B*, *SULT1A3*/*4* and *NPIP*. Transcriptome profiling of lymphoblastoid cell lines of deletion and duplication carriers and control individuals showed that all genes with detectable expression within the CNV, in particular the *BOLA2/2B*, *SLX1A/1B*, *SULT1A3/4* and *SLX1A/1B*-*SULT1A3/A4* read-through transcripts, were correlated positively with gene dosage. Further, these analyses implicated a potential role for ciliary dysfunction in the 16p11.2 600 kb BP4-BP5 pathology [20]. The reciprocal impacts on BMI as well as brain volume and structures involved in reward, language and social cognition indicated that the phenotypes could have mirror aetiologies depending on the observed changes in gene transcript levels.

To probe the mechanistic basis of the human genetic data, mouse models have been generated to study the correlation between phenotype and genotype. The first published mouse models of the 16p11.2 syndromes carry deletion and duplication of the *Slx1b-Sept1* region and are reported to display locomotor activity alterations and ventral midbrain volume changes [21]. Despite the presence of compatible phenotypes, the interval targeted in this model includes four genes outside the human BP4-BP5 interval, potentially posing interpretive challenges. In addition, the consequence of the duplication was not investigated comprehensively in this report. More recently, a second 16p11.2 mouse model carrying the deletion of the *Coro1a-Spn* region was generated, mutant animals display circuit defects in the basal ganglia [22]; these animals have a hearing deficit whereas no particular hearing defect has been reported in humans with 16p11.2 CNVs [7] or in the first mouse model [21]. Given the possibility that 1) such condition might alter the response to behavioral tests and 2) the second mouse model might carry a second mutation causing deafness, we decided to engineer and characterize novel 16p11.2 CNV mouse models that carry rearrangements of the *Sult1a1-Spn* interval on an inbred C57BL/6N genetic background, which corresponded to the BP4-BP5 syntenic region in humans. We reasoned that evaluation of the breakpoint region in the engineered deletion or duplication mouse would allow us to decipher if changes in the number of copies have an impact on the phenotypes compared to the two published 16p11.2 models. To understand better the phenotypic variability in individuals carrying the 16p11.2 deletion or duplication, we also posited that evaluation of our model on an inbred C57BL/6N background would allow us to study the genetic background influence between the three mouse model phenotypes. Metabolic response [23] is an exemplar of a phenotype strongly influenced by the inbred genetic background. We thus performed the phenotypic characterization on inbred (C57BL/6N) and hybrid (F1 C57BL/6NxC3B) genetic backgrounds. We first utilized a comprehensive set of tests to assess the impact of the genetic rearrangements on behavior and hippocampal synaptic function to determine whether the models recapitulate some of the cognitive deficits and autistic traits associated with the 16p11.2 syndromes. Second, since humans with deletion or duplication of the 16p11.2 locus display body weight changes (respectively obesity and leanness), we explored metabolism phenotypes in our mouse model on either a normal chow diet or with a high fat high sugar diet to challenge energy balance. Third, and to complete the phenotypic characterization of our models, we performed an analysis of craniofacial structure of the mutant mice. Finally we performed transcriptional analyses of different brain regions and the liver to understand better the consequences of the disease on brain and metabolic functions.

## Results

### Generation of mice carrying rearrangements of the *Sult1a1–Spn* genetic interval

**Fig 1:**
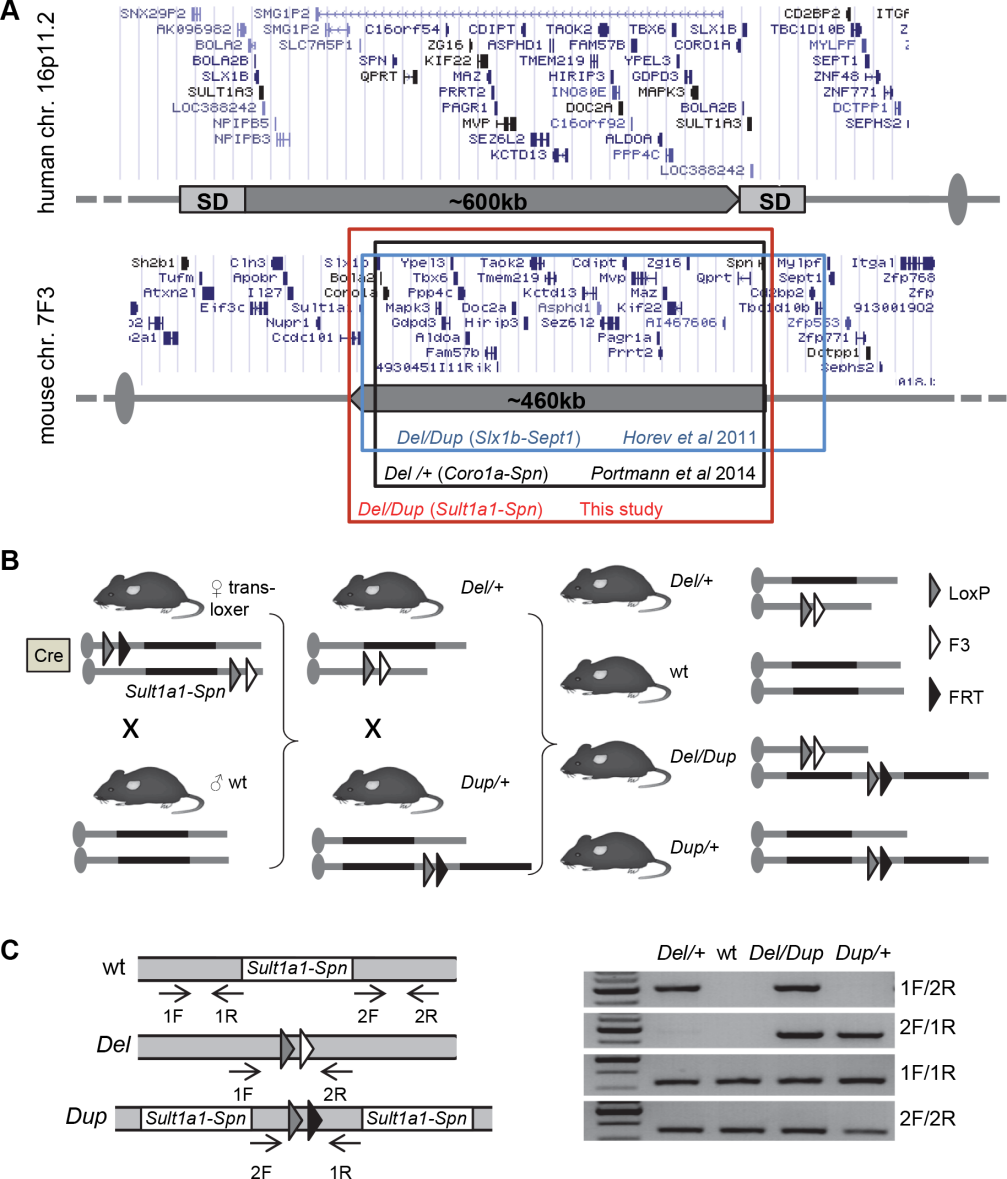
Mouse models for 16p11.2 rearrangements. (A) Top: human 16p11.2 region and proximal segmental duplications (SDs) prone to generate BP4-BP5 copy number variations (CNVs) by a non-allelic homologous recombination (NAHR) mechanism. All genomic positions are given according to UCSC human genome browser GRCh38/hg38. Bottom: 16p11.2 syntenic region on mouse chromosome 7F3. Colored boxes indicate genetic intervals studied in previous publications and in the present study. All genomic positions are given according to UCSC mouse genome browser GRCm38/mm10. (B) Strategy for *in vivo* cre-mediated recombination and targeted meiotic recombination (TAMERE) crossing strategy. LoxP sites were inserted upstream of *Sult1a1* and downstream of *Spn*. The breeding strategy aimed to have trans-loxer females expressing the *Hprt*^*tm1(cre)Mnn*^ transgene and carrying the two loxP sites in a *trans* configuration. The last step consisted of mating trans-loxer females with wt males to generate progeny carrying the deletion or the duplication of the *Sult1a1-Spn* region. *Del/+* and *Dup/+* animals were crossed with wt animals to generate *Del/+* and *Dup/+* cohorts. For the *Del-Dup* cohorts, we crossed *Del/+* with *Dup/+* to generate *Del/+*, wt, *Dup/+* and *Del/Dup* animals. (C) Molecular validation. PCR products specific for the *Dup/+* and *Del/+* alleles are 500-bp and 461-bp long, respectively.

The deletion (*Del/+)* and duplication (*Dup/+)* carrier mice were generated on the C57BL/6N (B6N) genetic background (see S1 File; Fig 1). The segregation of the *Del* allele (30.7%) was reduced considerably compared to the *Dup* allele (45.8%; Table 1). To confirm these results and characterize the mice carrying both the deletion and the duplication, we crossed *Del/+* with *Dup/+* animals. We confirmed that the low transmission of the *Del/+* allele was compensated in the *Del/Dup* carriers, thus demonstrating that lethality is associated with the deletion on the B6N background (Table 1). To evaluate the influence of the genetic background, we crossed B6N *Del/+* and *Dup/+* mice with sighted C3H/HeH (C3B) wild-type (wt) mice [24] and observed normal segregation of the *Del* (49.1%) and *Dup* (49.2%) alleles on the hybrid F1C57BL/6NxC3B (F1B6C3B) background (Table 1).

**Table 1:** Transmission rates of the *Sult1a1-Spn* deletion (*Del*) and duplication (*Dup*) alleles observed at weaning.

Genetic background	Cross	Genotype	Number observed	Observed ratio	χ^2^	*P*
C57BL/6N	*Del/+* × wt	wt	230	69.3%	24.7	6.79x10^−07^
		*Del/+*	102	30.7%		
C57BL/6N	*Dup/+* × wt	wt	129	54.2%	0.84	0.36
		*Dup/+*	109	45.8%		
C57BL/6N	*Del/+* × *Dup/+*	wt	117	34.6%	12.5	4.07x10^−04^
		*Del/+*	39	11.6%	24.5	7.43x10^−07^
		*Dup/+*	90	26.6%	0.36	0.55
		*Del/Dup*	92	27.2%	0.67	0.41
C57BL/6N ×C3B	*Del/+* × wt	wt	80	49.1%	0.03	0.87
		*Del/+*	83	50.9%		
C57BL/6N ×C3B	*Dup/+* × wt	wt	30	50.8%		
		*Dup/+*	29	49.2%	0.01	0.93

On the C57BL/6N genetic background, the *Del* allele showed a reduced transmission rate. The combination of both *Del* and *Dup* alleles led to the rescue of the *Del* allele-associated lethality. On the C57BL/6N×C3B genetic background, no lethality was associated with the *Del* allele.

To determine whether B6N *Del/+* died *in utero* or postnatally, the fetuses of pregnant wt females sired by *Del/+* males were collected at embryonic day 18.5 (E18.5), a few hours before natural delivery. Among the 69 fetuses extracted by caesarean section, we found a normal Mendelian ratio of 37 wt and 32 *Del/+* foetuses. Overall, *Del/+* fetuses required more time to oxygenize, and 3 mutant animals died within several minutes after a caesarean section. Furthermore, *Del/+* were underweight in comparison with wt littermates, an observation which has been reported in human 16p11.2 del newborns [9] (*Del/+*: 1.08 ± 0.02 g, n = 32; wt: 1.18 ± 0.01 g, n = 37; F_(1,67)_ = 27.318, *P* < 0.001, S1A Fig). In contrast, weight was not affected by the *Dup/+* mutation (*Dup/+*: 1.22 ± 0.02 g, *n* = 27; wt: 1.22 ± 0.01 g, *n* = 24; F_(1,49)_ = 0.047, *P* = 0.829, S1A Fig). Furthermore, the *Del/+* allele on the C57BL/6N genetic background induced a developmental delay with weight defect that led to the death of approximately 55% of the *Del/+* neonates between birth and weaning.

### Rearrangements of the *Sult1a1–Spn* region induce opposite phenotypes on locomotor activity, repetitive behaviors, and memory performance

**Fig 2:**
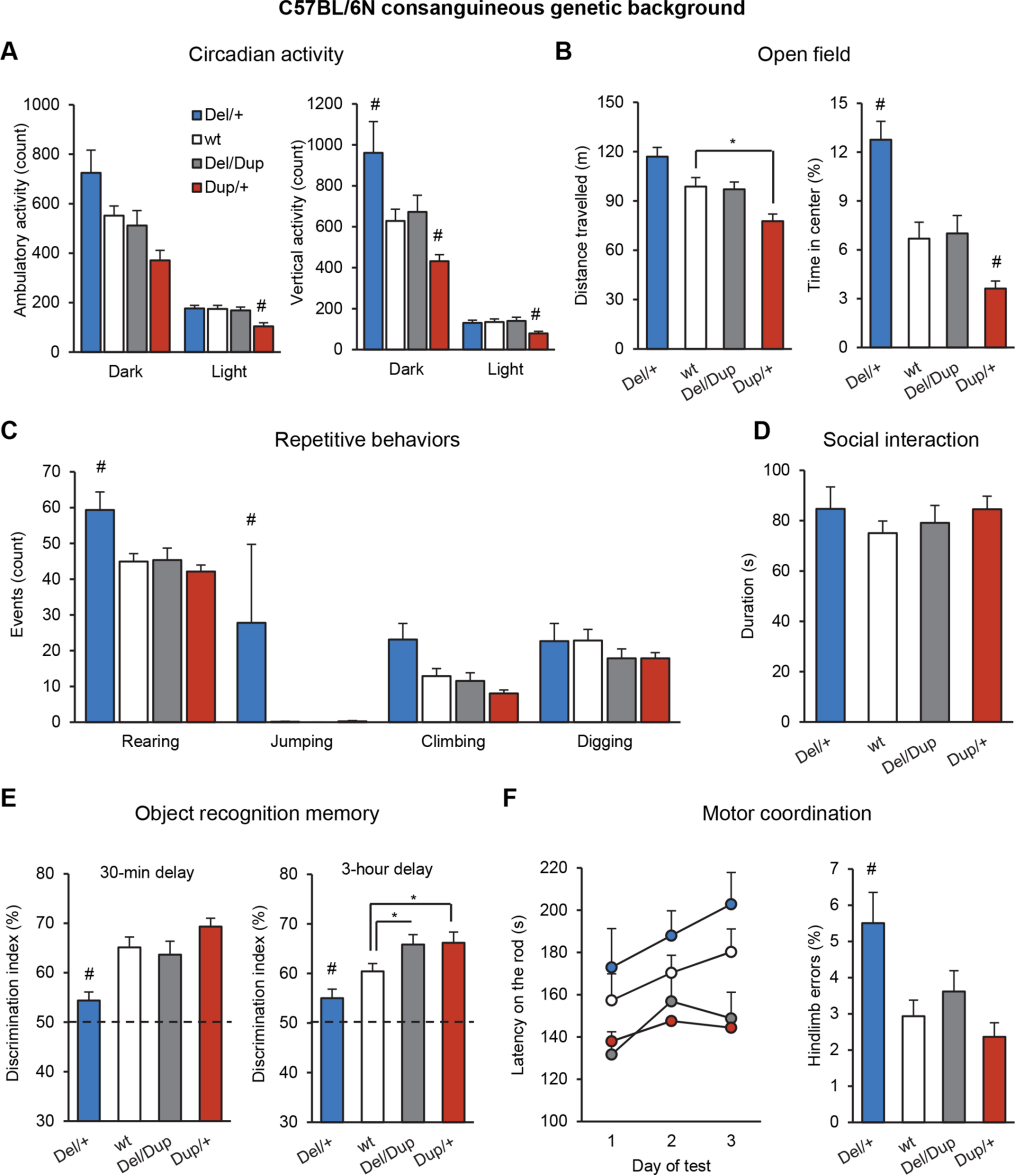
Behavioral characterization of the *Del-Dup* cohort. (A) Circadian activity test. The light-dark cycle was set as 12-h light and 12-h dark (lights on at 7 am). Plots represent counts of spontaneous locomotor activity and rearing behavior during the dark and light phases. (B) Open field test results illustrate exploratory locomotor activity (distance travelled in m) and percentage of time spent in the central area over 30 min of test. (C) Repetitive behaviors are illustrated by the occurrences of rearing, jumping, climbing and digging behaviors during 10 min of observation in a novel home cage. (D) The social interaction test graph indicates the duration of social interaction behaviors (sniffing and following) between pairs of unfamiliar mice of the same genotype and equivalent body weight tested in a familiar open field area during 10 min. (E) Results of the novel object recognition test. Discrimination index reflects the ability of mice to distinguish the novel object from the familiar object after a short (30 min) and a long (3 hours) retention delay. All genotypes performed significantly above the chance level (one-sample Student's *t*-test). (F) Motor coordination evaluation. The left graph illustrates the latency (s) with which mice stayed on the rod before falling under accelerating speed (4–40 rpm in 5 min) over 3 consecutive days of testing. The right graph shows hindlimb errors during the notched bar test. Animals had to cross a notched bar and each time a hind paw went through a gap, it was counted as an error. The data are represented as the mean + s.e.m., *P < 0.05 vs wt and ^#^ *P* < 0.05 vs all other groups. Tukey's and Mann-Whitney *U*-tests were applied following a significant one-way ANOVA and Kruskal-Wallis results, respectively.

We first studied the effects of the *Sult1a1-Spn* deletion and duplication on behaviour with independent cohorts of young adult mice bred on an inbred C57BL/6N genetic background. Complete description of behavioral data is reported in the supplementary information (S1 and S2 Tables, S2 Fig). To confirm these data and to study the behavior of mice carrying both deletion and duplication of the 16p11.2 syntenic region, we generated and characterized a compound *Del-Dup* cohort with littermates of 4 genotypes: *Del/+*, wt, *Del/Dup*, and *Dup/+* (S3 and S4 Tables). Our behavioural tests revealed an opposite effect of *Sult1a1-Spn* deletion and duplication on several traits, including locomotor activity (Fig 2A and 2B), repetitive behaviors (Fig 2C), and learning and memory performance (Fig 2E).

*Del/+* and *Dup/+* mice had normal circadian activity and feeding behaviors (S3 Fig), but during the dark phase, *Del/+* mice showed increased spontaneous locomotor activity and rearing behavior compared to wt (Kruskal-Wallis analysis and Mann-Whitney *U*-test H_(3,51)_ = 15.803, *P* = 0.001; *Del/+* vs wt: *P* = 0.039; Fig 2A). In contrast, *Dup/+* mice manifested reduced locomotor activity during the light phase in comparison to wt animals (one-way ANOVA and Tukey’s *post-hoc* test F_(3,51)_ = 5.724, *P* = 0.002; *Dup/+* vs wt: *P* = 0.003) and reduced vertical (rearing) activity during the dark phase (H_(3,51)_ = 15.803, *P* = 0.001, *Dup/+* vs wt: *P* = 0.015) and light (H_(3, 51)_ = 11.119, *P* = 0.011; *Dup/+* vs wt: *P* = 0.008;Fig 2A). The water and pellet consumption were normal but the pellet loss was increased in *Del/+* mice from separated cohort (S4E Fig). When tested in the open field, only *Dup/+* mice had significantly different locomotor activity scores from those of wt animals (F_(3,58)_ = 8.920, *P* < 0.001; *Del/+* vs wt: *P* = 0.089; *Dup/+* vs wt: *P* = 0.013; Fig 2B). The time spent in the center of the arena, a measure of emotional behavior, was increased in *Del/+* mice and decreased in *Dup/+* mice (H_(3,58)_ = 23.758, *P* < 0.001; *Del/+* vs wt: *P* = 0.001; *Dup/+* vs wt: *P* = 0.029; Fig 2B). The *Del/+* mice also spent more time in the center of the arena in the last 5 min of the test compared to the first 5 min (S4 Fig). Visual observations of the animals in their home cages revealed a range of abnormal repetitive behaviors in the *Del/+* mice (Fig 2C). We recorded a marked increase in rearing (F_(3,41)_ = 6.156, *P* = 0.001; *Del/+* vs wt: *P* = 0.010) and jumping (H_(3,41)_ = 14.394, *P* = 0.002; *Del/+* vs wt: *P* = 0.016) behaviors in these mice, whereas no behavioral abnormalities were detected in the *Dup/+* animals. *Del/Dup* mice carrying one copy of both rearrangements behaved similarly to wt animals (*P* > 0.05 for all measures; Fig 2A–2C), suggesting that abnormal activity phenotypes of *Del/+* and *Dup/+* mice were due to altered gene dosage from the *Sult1a1-Spn* region. In the social interaction test, we observed no difference in sniffing or following time between genotypes (Fig 2D).

Next, we evaluated our models in the novel object recognition test, the most common assay of the various facets of recognition memory in rodents. We first investigated whether *Del/+* and *Dup/+* mice could discriminate a novel object from a previously explored object after a short retention delay of 30 min (Fig 2E). During the acquisition session, mice of all genotypes spent an equal amount of time exploring the sample object (S4 Table). In the subsequent choice session, *Del/+* mice displayed a significant memory impairment compared to wt, whereas *Dup/+* mice tended to display a memory improvement (F_(3,49)_ = 8.080, *P* < 0.001; *Del/+* vs wt: *P* = 0.004). To explore this cognitive phenotype further, we extended the retention delay to 3 h (Fig 2E). The *Del/+* mice again displayed a poor recognition performance, whereas *Dup/+* mice showed a memory improvement compared to wt mice (H_(3,51)_ = 16.014, *P* = 0.001; *Del/+* vs wt: *P* = 0.043, *Dup/+* vs wt: *P* = 0.021, *Del/Dup* vs wt: *P* = 0.048). Interestingly, *Del/Dup also* displayed improved performance in this test, similar to *Dup/+* mice (*P* < 0.05).

We employed a series of assays to investigate potential alterations in sensory and motor functions. No significant differences in motor coordination and motor learning were detected between mutant and wt mice in the rotarod test (Fig 2F). In the notched bar test, the *Del/+* mice made a significantly higher number of errors compared to wt, whereas *Dup/+* and *Del/Dup* mice performed normally (H_(3,60)_ = 14.044, *P* = 0.003; *Del/+* vs wt: *P* = 0.007; Fig 2F). In the grip test, *Del/+* and *Dup/+* mice showed stronger and weaker grip strength respectively, compared to wt (F_(3,60)_ = 23.598, *P* < 0.001; *Del/+* vs wt: *P* = 0.002; *Dup/+* vs wt: *P* < 0.001; S2 Table**)**. Nevertheless, no difference between wt and mutant mice was observed in measuring *in situ* tibialis anterior (TA) isometric contraction in response to nerve stimulation (S55C–S5E Fig). In addition, we observed normal fiber size and normal succinate dehydrogenase (SDH) staining in TA muscle (S5F Fig). Finally, we evaluated the hearing discrimination of animals in the ABR test and detected no abnormal phenotypes in the mutant mice (S6A and S6B Fig).

### Influence of genetic background on behavioral phenotypes associated with *Sult1a1–Spn* interval rearrangements

**Fig 3:**
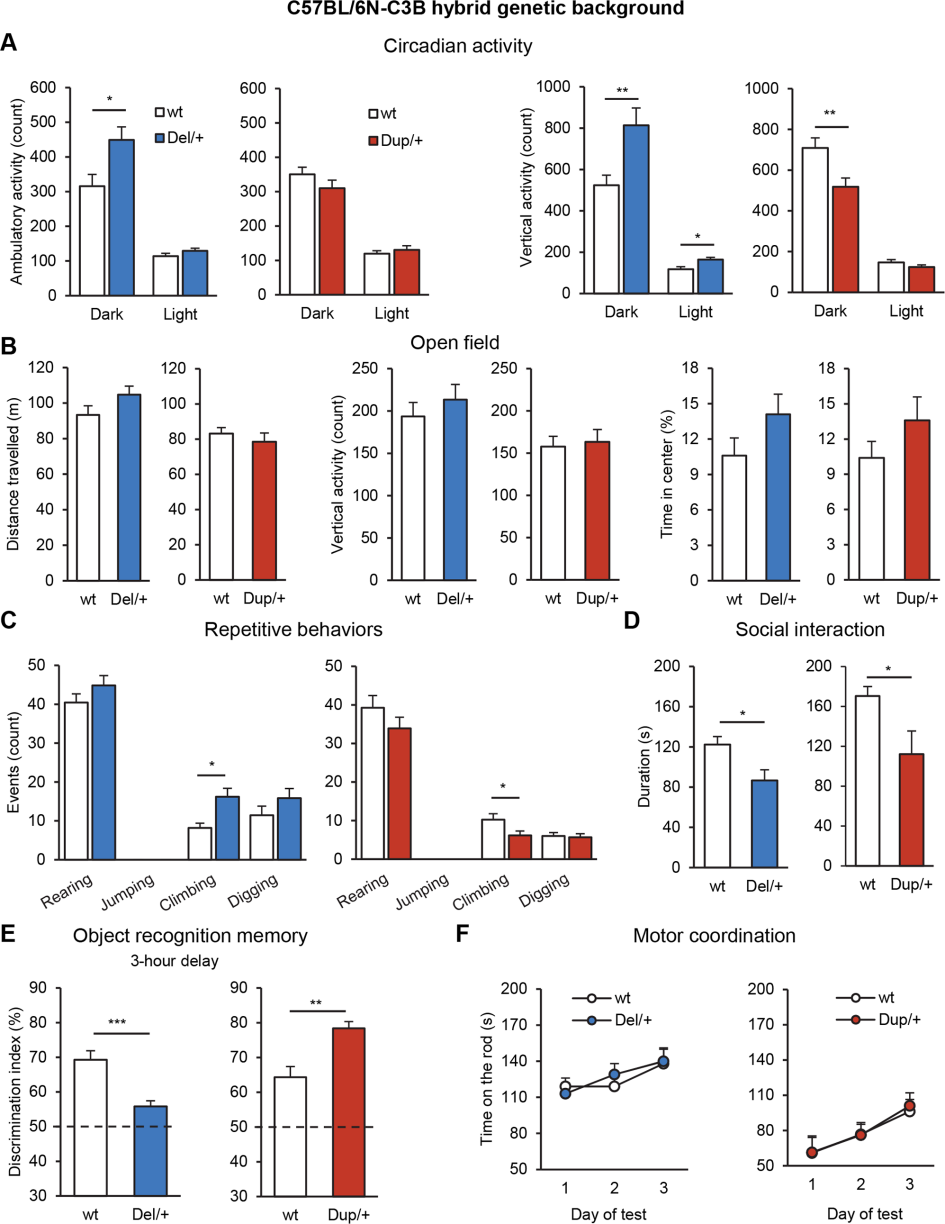
Behavioral characterization of the separated C57BL/6NxC3B *Del/+* and *Dup/+* cohorts. (A) Circadian activity test. Graphs plot the ambulatory activity (count) and the vertical activity/rears (count) during dark and light phases. (B) Open field test. Distance travelled (m), vertical activity/rears (count) and time percentage spent in the central area over 30 min of testing. (C) Repetitive behavior data. Observations of rearing, jumping, climbing and digging behaviors during 10 min of observation in a novel cage. (D) Social interaction test. Graph plots the duration of sniffing and following behaviors. (E) Novel object recognition test. Discrimination index was calculated as the ratio of time spent exploring the novel object vs the familiar object in the choice trial. (F) Motor coordination evaluation. Graphs plot the latency (s) that mice stayed on the rod before falling under acceleration speed over 3 consecutive days of test. Data are represented as the mean + SEM. *P < 0.05, **P < 0.01 and ***P < 0.001, significantly different from matched wt littermates, Student’s t-test.

Humans with 16p11 CNVs present autistic traits. Although hyperactive and repetitive behaviors were found during the study on the C57BL/6N genetic background, we asked whether the observed behavioral phenotypes were sensitive to the genetic background. Thus, we repeated our behavioral analyses on mutant F1 animals with a C57BL/6N×C3B genetic background (Fig 3, S6 and S7 Tables). Sep^2^arate cohorts of *Del/+*, *Dup/+* and their corresponding wt littermates were tested. As expected, *Del/+* and *Dup/+* mice on a hybrid genetic background showed a range of behavioral abnormalities compared to their wt counterparts. Opposite phenotypes were noted for rearing in the circadian activity test (Fig 3A), climbing behavior (Fig 3C), and cognitive capacity (Fig 3E). *Del/+* mice demonstrated recognition memory deficits in the novel object recognition task with a 3 h retention delay as well as lack of object-place recognition memory (Fig 3E, S7 Table). Recognition memory improvements were also confirmed for *Dup/+* mice (Fig 3E). Compared to wt mice, social interaction was reduced in both *Del/+* (F_(1,9)_ = 6.799, *P* = 0.028) and *Dup/+* (F_(1,10)_ = 5.594, *P* = 0.040) mutant mice (Fig 3D). In the three-chambered social procedure, mutant and wt mice displayed similar exploration time of the first stranger (S7 Table). However, in comparison to wt, *Del/+* mice showed deficits of social preference for the second stranger (F_(1,14)_ = 12.849, *P* = 0.003). To validate these social phenotypes, we verified the olfactory discrimination of *Del/+* and *Dup/+* mice after the exposure to social and non-social odors on the C57BL/6N genetic background. No genotype effect was found for these parameters (S6C and S6D Fig).

### Effects of rearrangements of the *Sult1a1–Spn* region on synaptic transmission in hippocampal slices

**Fig 4:**
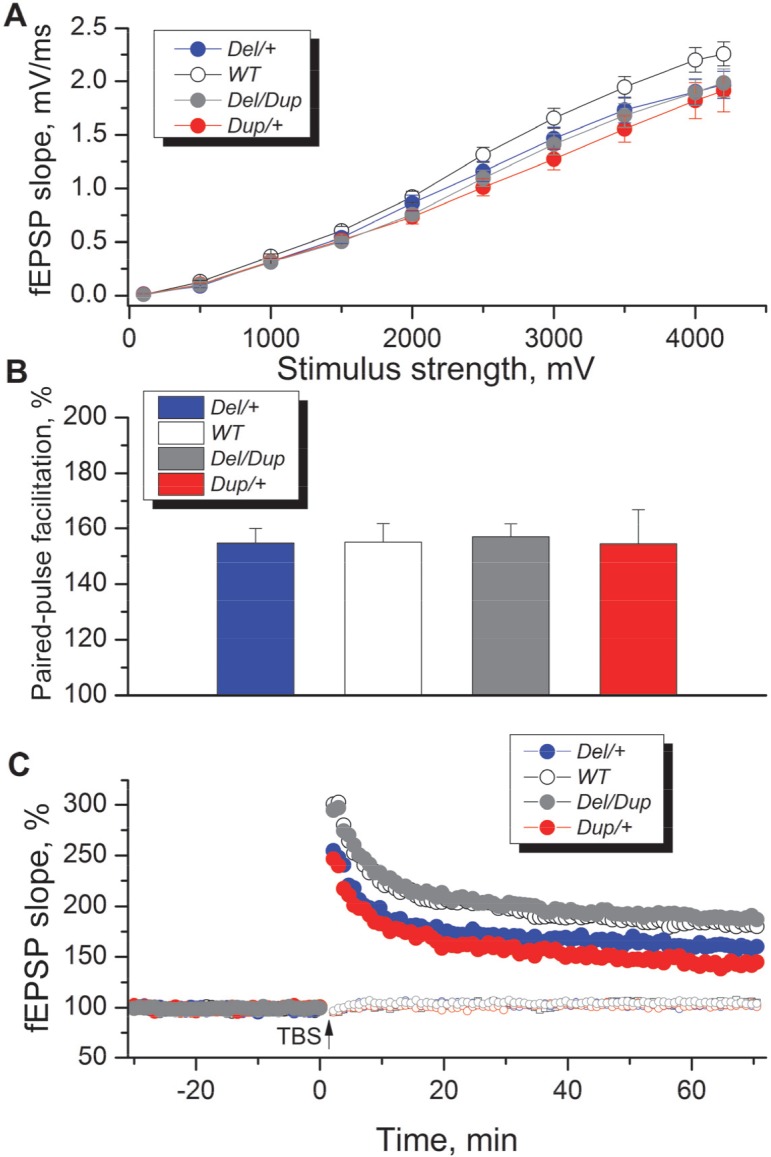
Effects of *Sult1a1*–*Spn* region rearrangements on electrophysiological parameters measured in the CA1 area of hippocampal slices. (A) Baseline synaptic transmission was slightly decreased in mice with *Del/+*, *Dup/+* and *Del/Dup* mutations, but no significant overall genotype effect was observed (F_(3, 52)_ = 1.37; *P* = 0.261; RM ANOVA of data in individual slices). Input-output relationships illustrate averaged fEPSP slopes in slices from *Del/+* (*n* = 10; *N* = 4), *Dup/+* (*n* = 10; *N* = 4), *Del/Dup* (*n* = 19; *N* = 6) and wt mice (*n* = 18; *N* = 8) in response to stimulation of Schaffer collaterals by biphasic voltage pulses of 0.1–4.2 V. (B) Paired-pulse facilitation in slices from mice with *Sult1a1*–*Spn* region rearrangements was normal and no significant genotype effect was detected (F_(3, 35)_ = 0.0298; *P* = 0.993, two-way nested ANOVA). (C) Theta-burst stimulation elicited pathway-specific long-term potentiation of synaptic transmission in the hippocampal CA1 area. The normalized magnitude of this potentiation 60–65 min after LTP induction was influenced by the genotype (F_(3, 35)_ = 3.43; *P* = 0.027, two-way nested ANOVA). *Post hoc* Dunnett’s Multiple Comparison test on values in individual slices demonstrated that LTP was significantly smaller (*Q* = 2.441, *P* < 0.05) in slices from *Dup/+* mice (*n* = 10; *N* = 4) compared to wt slices (*n* = 18; *N* = 8), whereas levels of LTP in *Del/+*(*n* = 10; *N* = 4) and *Del/Dup* slices (*n* = 19; *N* = 6) did not differ significantly from wt levels. The data are expressed as the mean + SEM. Overall effect of genotype was determined, as appropriate, by repeated measures (A) or two-way nested ANOVA (B, C) and *post hoc* comparisons to wt values were made using Dunnett’s multiple comparison test.

Our behavioral studies revealed strong opposite phenotypes of recognition memory in both inbred and hybrid genetic backgrounds. To investigate the potential electrophysiological underpinnings of the observed phenotypes, we studied synaptic transmission and its plasticity in synapses between Schaffer collaterals and apical dendrites of CA1 pyramidal neurons in the hippocampus, the principal region implicated in spatial memory [25–27]. This experiment was performed on hippocampal slices of wt, *Del*/+, *Dup*/+ and *Del*/*Dup* mice on an inbred C57BL/6N genetic background. The analysis of input-output relationships (Fig 4A) showed that there was no significant interaction between genotype and stimulus (repeated measures ANOVA F_(27,468)_ = 0.98; *P* = 0.498). Although we observed nominally decreased slopes of field excitatory postsynaptic potentials (fEPSPs) in *Del*/+ and *Dup*/+ mutants, especially in response to higher stimulus strengths (Fig 4A), a separate two-way nested ANOVA performed on maximum fEPSP values similarly failed to reveal a significant effect of genotype (F_(3, 35)_ = 1.229, *P* = 0.314).

Likewise, genotype did not influence significantly the values of paired-pulse facilitation, a model of short-term synaptic plasticity (F_(3,35)_ = 0.0298; *P* = 0.993; Fig 4B). To investigate long-term synaptic plasticity, we induced long-term potentiation (LTP) of fEPSPs in Schaffer collaterals-CA1 synapses by theta-burst stimulation (Fig 4C). A two-way nested ANOVA demonstrated a significant effect of genotype on LTP values (F_(3, 35)_ = 3.43; *P* = 0.027). *Post hoc* Dunnett’s Multiple Comparison test performed on individual slice values demonstrated that LTP was significantly smaller (*Q* = 2.441; *P* < 0.05) in slices from Dup/+ mice (140 ± 9%, slice number *n* = 10, animal number *N* = 4) compared to wt slices (177 ± 7%, *n* = 18; *N* = 8), while levels of LTP in *Del*/+ (160 ± 12%, *n* = 10; *N* = 4) and *Del*/*Dup* slices (182 ± 11%, *n* = 19; *N* = 6) did not differ significantly from wt levels. We concluded that long-term synaptic plasticity is sensitive to the duplication of the *Sult1a1-Spn* region, whereas neither short- nor long-term plasticity are affected by deletion or deletion/duplication rearrangements. We also observed a trend to nominally lower fEPSP slopes in all three mutants compared to wt values. The latter observation may require further experiments with larger cohorts.

### Rearrangements of the *Sult1a1–Spn* region induce opposite effects on weight and adiposity

**Fig 5:**
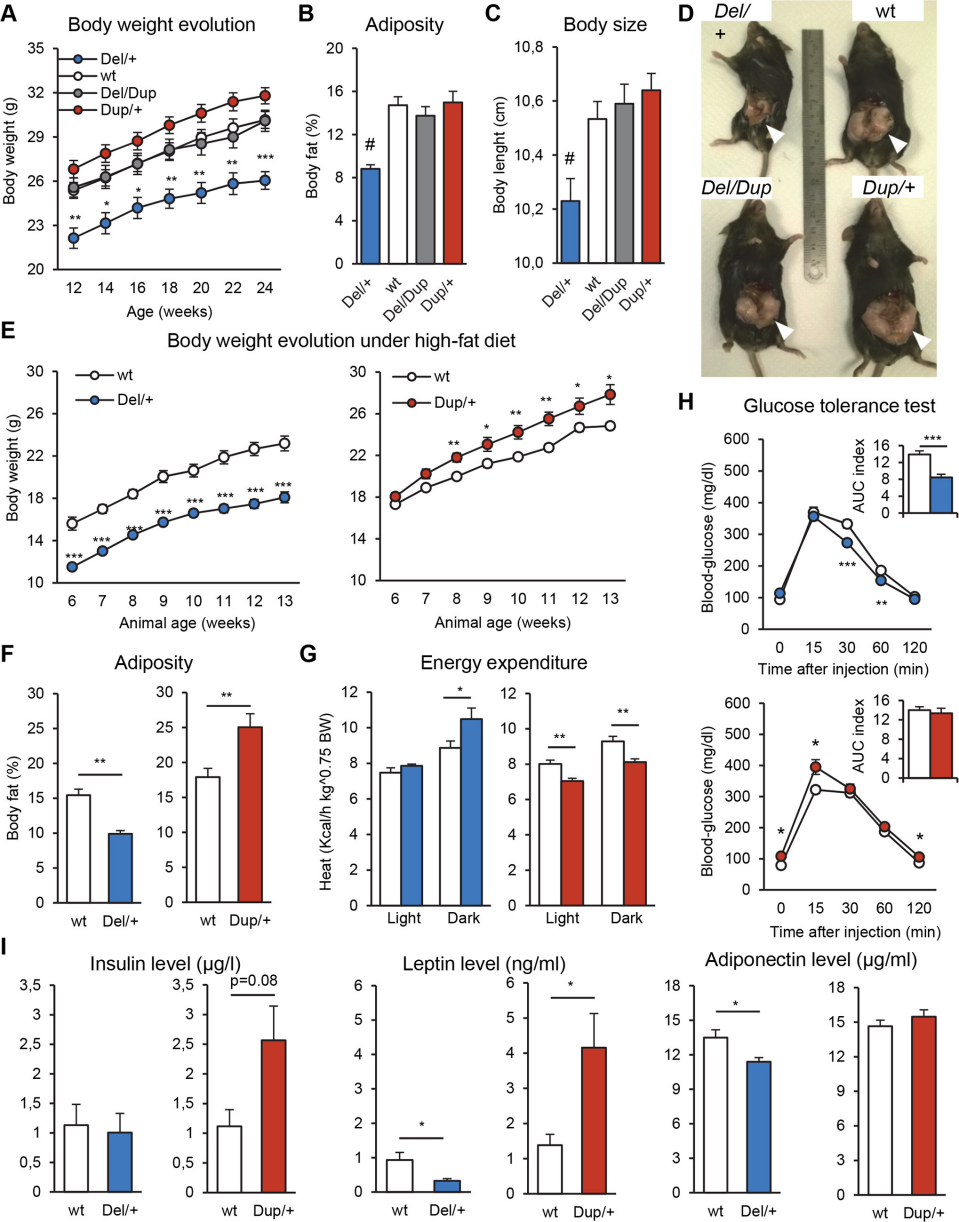
Analysis of weight and adiposity with the standard and high-fat diets. **(A-D) Weight, adiposity and body length of the *Del-Dup* littermates when fed a standard diet.** (A) Evolution of the body weight (g) of adult animals. (B) Body fat percentage of 20-week-old animals measured by qNMR. (C) Body length (distance from the snout to the tail base) of 20-week-old animals. (D) Adult male *Del/+* (21.9 grams), +/+ (31 g), *Del/Dup* (33.9 g) and *Dup/+* (35.8 g) littermates at 24 weeks of age. The *Del/+* mice were shorter and showed a marked lack of epididymal adipose tissues (white arrows). (E) Body mass data measured over time (g). (F-I) Metabolic and endocrinological analysis of separate *Del/+* and *Dup/+* cohorts on the C57BL/6N background put on the high-fat diet at 5 weeks of age. (F) Body fat percentage of 13-week old animals measured by qNMR. (G) Energy expenditure (Kcal/h per kg^0.75 of body weight) during 12-h dark and 12-h light phases. (H) Intraperitoneal glucose tolerance test. Blood glucose (mg/dl) and glucose area under the curve (AUC) index (min × mg / dl × 0.001) (I) Endocrinological analysis. Blood insulin (μg/l), leptin (ng/ml) and adiponectin (μg/ml) levels. The data are presented as the mean + SEM. (A, E) Repeated Measures ANOVA “genotype” analysis, Tukey’s test. (B) Kruskal-Wallis analysis, Mann-Whitney *U*-test. (C) One-way ANOVA analysis, Tukey’s test. (F-I) Student’s *t*-test. *P < 0.05, **P < 0.01, ***P < 0.001 vs wt and ^#^ *P* < 0.05 vs all other groups.

Individuals with 16p11 CNVs present with mirror BMI phenotypes [28]. To determine whether our mouse models recapitulated this phenomenon, body weight of the adult animals was recorded once a week during behavioral analyses. In the first separated cohorts, C57BL/6N *Del/+* mice were significantly underweight (two-way ANOVA genotype effect F_(1,120)_ = 88.115, *P* < 0.001; S1B Fig), whereas C57BL/6N *Dup/+* mice were significantly overweight compared to wt littermates (two-way ANOVA genotype effect F_(1,99)_ = 8.391, *P* = 0.009; S1B Fig). Similar results were observed when we generated the two genotypes at the same time in the *Del-Dup* cohort (Fig 5A). In comparison with wt and *Del/Dup* mice, *Del/+* littermates were underweight, whereas *Dup/+* mice showed trends for overweight (two-way ANOVA genotype effect F_(3,780)_ = 9.954; *P* < 0.001; *Del/+* vs wt: *P* = 0.002; *Dup/+* vs wt: *P* = 0.211). Feeding behaviors evaluated during the circadian activity test were similar in mutant and wt animals (S3C and S3D Fig). In addition, no correlation was observed between the activity and body mass for the different genotypes. We evaluated body fat percentage of live animals by qNMR (quantitative nuclear magnetic resonance) and found a decrease in adiposity in the *Del/+* mice (H_(3, 60)_ = 22.770, *P* < 0.001; *Del/+* vs wt: *P* < 0.001; Fig 5B); these data were confirmed in sacrificed animals which displayed a lack of visceral fat pads (Fig 5D). Body size was reduced in the *Del/+* mice (F_(3,35)_ = 6.834, *P* < 0.001; *Del/+* vs wt: *P* = 0.027; Fig 5C), which correlated with the animal weight (Pearson correlation coefficient, ρ = 0.893, *P* = 0.001). No weight, body fat, or size modifications were noted in *Del/Dup* mice.

On an F1 (C57BL/6N × C3B) hybrid background, *Del/+* animals were still underweight in comparison to wt littermates (two-way ANOVA genotype effect F_(1,456)_ = 16.620, *P* < 0.001; S1D Fig), whereas no body weight phenotype was observed in the *Dup/+* animals. Adiposities were similar between mutant and control mice (S1E Fig). A body size reduction was confirmed in the *Del/+* mice (F_(1,33)_ = 17.137, *P* < 0.001; S1F Fig), but no correlation between body size and body weight was observed.

To characterize further weight and adiposity changes, we performed a metabolic analysis of the C57BL/6N *Del/+* and *Dup/+* separate cohorts challenged with a high-fat diet between 5 and 15 weeks of age (Fig 5E–5I, S8 Table). In comparison to wt animals, the *Del/+* mice were underweight (two-way ANOVA genotype effect F_(1,102)_ = 48.671, *P* < 0.001; Fig 5E), whereas the *Dup/+* mice were overweight (two-way ANOVA genotype effect F_(1,112)_ = 8.674, *P* = 0.010). Body size was decreased in *Del/+* mice (F_(1,17)_ = 51.771, *P* < 0.001; S8 Table) and increased in *Dup/+* mice (F_(1,16)_ = 29.229, *P* < 0.001). Body composition analysis revealed a decrease in fat percentage in the *Del/+* mice (F_(1,16)_ = 32.138, *P* < 0.001; Fig 5F) and an increase in the *Dup/+* mice (F_(1,16)_ = 8.621, *P* = 0.010). No correlation was found between weight and adiposity in any of the genotypes. The energy expenditure (EE) of the animals was analysed by indirect calorimetric measure during the dark and light phases (Fig 5G). The *Del/+* mice showed a higher EE during the dark phase (F_(1,16)_ = 5.313, *P* = 0.035), whereas the *Dup/+* mice showed a lower EE during the dark (F_(1,15)_ = 12.994, *P* = 0.003) and light phases (F_(1,15)_ = 13.997, *P* = 0.002). These results are consistent with endogenous activity phenotypes of mutant mice observed in the circadian activity test. The intraperitoneal glucose-tolerance test (IPGTT) indicated a faster glucose clearance in the *Del/+* mice (F_(1,15)_ = 23.396, *P* < 0.001; Fig 5H) and hyperglycemia in the *Dup/+* animals (F_(1,16)_ = 8.220, *P* = 0.011). Consistent with the body fat composition data, endocrinology analysis (Fig 5I) revealed decreased blood levels of leptin (F_(1,16)_ = 7.059, *P* = 0.017) and adiponectin (F_(1,17)_ = 6.790, *P* = 0.018) in the *Del/+* mice and an increase of leptin blood levels in the *Dup/+* mice (F_(1,15)_ = 5.350, *P* = 0.035). A nominal increase in insulin blood levels, which failed to reach statistical significance, was also noticed in the *Dup/+* mice. Blood chemistry analysis did not reveal gross hematology changes except for a decrease of free fatty acid levels in the *Del/+* mice (F_(1,17)_ = 5.333, *P* = 0.034; S8 Table).

Finally, we repeated the high-fat diet protocol in the *Del/+* animals on the hybrid C57BL/6N×C3B background (S7 Fig, S9 Table). We observed that F1 *Del/+* mice showed similar weight, fat, energy expenditure, glucose clearance and endocrinology phenotypes in comparison to the *Del/+* mice on the C57BL/6N background (S7A–S7E Fig). C57BL/6N×C3B *Del/+* animals also presented with a basal hyperglycemia, higher blood levels of calcium and lower blood levels of total cholesterol and glucose (S7F Fig). Bomb calorimetric analysis of mouse feces revealed a similar level of food energy usage between the wt and the *Del/+* mice (S7G Fig).

### Rearrangements of the *Sult1a1–Spn* region induce craniofacial dysmorphologies

In addition to neuropsychiatric and BMI features, patients with 16p11.2 CNVs also present with brain volume changes and craniofacial malformations [19]. We studied the influence of 16p11.2-homologous CNVs on mouse craniofacial structure by analysing computed tomography (CT) cranial scans of animal heads combined with ulterior reconstruction of 3D skull images using 39 cranial landmarks (S8A Fig). Separate *Del/+* and *Dup/+* cohorts of females were used for the Euclidian distance matrix analysis [29]. A global skull effect was observed in the *Del/+* females with a significantly reduced skull size (*T* = 0.009; S8B Fig) and altered skull shape (*Z* = 0.046; S8C Fig). Most affected regions corresponded to the premaxilla, maxilla and zygomatic human bones, indicating an important alteration of *Del/+* facial features. Although the skull size of the *Dup/+* animals was unchanged (*T* = 0.297; S8D Fig), their skull shape was altered significantly (*Z* = 0.037; S8E Fig).

### Transcriptomic analysis

**Fig 6:**
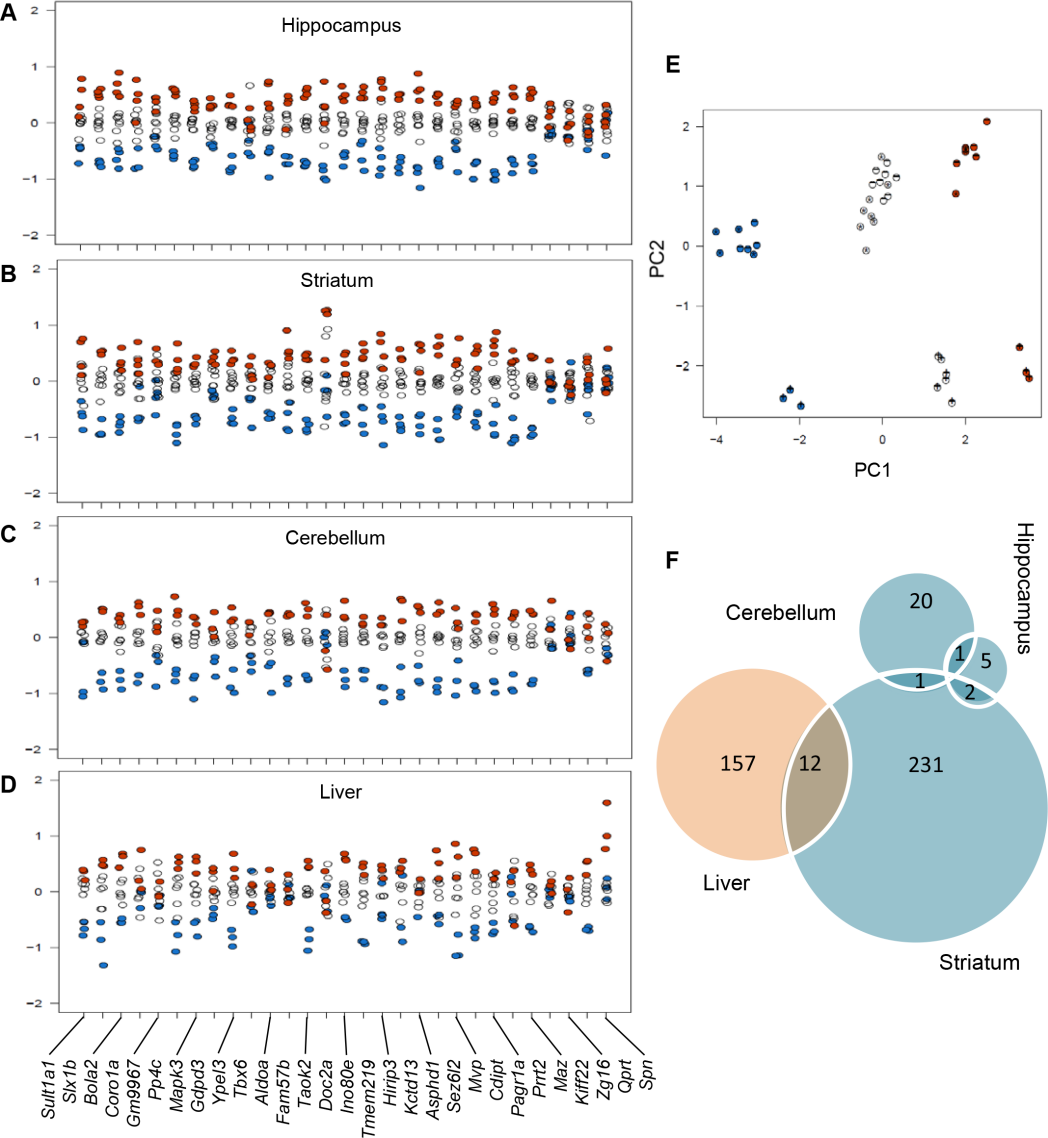
Expression levels of genes within the *Sult1a1-Spn* region in brain and peripheral tissues. Expression levels for 30 probe sets within the engineered region in the hippocampus (A), the striatum (B), the cerebellum (C), and the liver (D). Vertical axis represents log2-fold change in the normalized expression ratio. Blue dots—*Del/+* animals, black circles—wt animals, orange dots—*Dup/+* animals. (E) Principal component analysis of the expression of genes from the *Sult1a1-Spn* region. The first component (horizontal axis) explaining 58.2% of the variance is mainly capturing the CNV effect, the second component (vertical axis) explaining 28.7% of the variance is mainly capturing the regional effect, especially in the cerebellum (+) versus the striatum (*) and the hippocampus (-). Blue dots—*Del/+* animals, black circles—wt mice, orange dots—*Dup/+* animals. (F) A Venn diagram representing the number of gene set enriched pathways identified using the transcriptome analysis in the three regions of the brain and the liver, showing some major impact in the striatum and the liver and 16 pathways found altered in at least two tissues.

To identify altered pathways in the 16p11.2 mouse models, we performed transcriptomic analysis of three brain regions (hippocampus, striatum, and cerebellum) and of one peripheral tissue (liver) in *Del/+*, *Dup/+* and wt animals (data accessible at NCBI GEO database[30], accession GSE66468). We assessed expression of the genes encompassed by the *Sult1a1-Spn* region boundaries and found that the majority of them showed mRNA levels proportional to the CNV copy number (5). The expression of the genes in the *Sult1a1-Spn* region was largely affected by the CNVs in all three brain regions and the liver, with the following exceptions: *Kif22*, *Qprt*, *Spn*, and *Zg16* appeared to be under dosage compensation in all tissues studied, whereas *Aldoa*, *Coro1a*, *Fam57b*, *Prrt2*, and *Tbx6* expression levels were only compensated in the liver (Fig 6A–6D). Furthermore, principal component analysis suggested that the effect of the CNV on the expression of *Sult1a1-Spn* interval genes was stronger in the *Del/+* animals (Fig 6E). At a 5% false discovery rate (FDR), between 7 and 14 probe sets mapping elsewhere on the genome were associated with the CNV dosage (9–32 at 10% FDR). We then performed gene set enrichment analysis (GSEA) on the genes ranked by the results of the differential expression analysis to identify the sets of genes that were positively or negatively associated with the *Sult1a1-Spn* copy number.

Our GSEA analysis considers the pre-ranked gene list according to the LIMMA results based on the dosage model. We used the MSigDB C2 gene set database (c2.all.v4.0.symbols.gmt), which collects curated gene sets from online pathways, and publications in PubMed. The data with an FDR below 25% are reported in S10 Table. In the hippocampus, two gene sets were dosage-dependently upregulated (upregulated in the *Dup/+* mice and downregulated in the *Del/+* mice) including genes from midbrain markers [31] and Akt1 signalling via mTOR [32]. In contrast, six gene sets were downregulated in a dose-dependent manner (upregulated in the *Del/+* mice and downregulated in the *Dup/+* mice) including the targets of Creb1 (a transcription factor induced in brain reward pathways after chronic exposure to recreational drugs [33]) as well as the genes involved in resistance to Ifna2 [34], in the response to TSA and butyrate [35, 36], and in the PID P38alpha/beta pathway [37]. A total of 22 gene sets were affected in the cerebellum including gene sets that were downregulated exclusively in a dosage-dependent fashion. In the striatum, 240 gene sets were dose-dependently upregulated. Six gene sets were dose-dependently downregulated, including genes involved in cocaine reward [33], involved in the regulation of inhibitory GABAergic synapse through NpaS4 [38], regulated by exercise [39], or regulated during the EGF response [40]. In the liver, a total of 163 gene sets were dose-dependently upregulated, including genes involved in adipogenesis [41, 42] and targets of PPARg [43]. Six gene sets were dose-dependently downregulated, including genes involved in the cholesterol biosynthesis and genes involved in lipid and carbohydrate metabolism regulated by HNF1a [44] or by Klf10 [45]. Fourteen pathways were found to be misregulated in different tissue types, including 12 pathways deregulated in the striatum and the liver and two pathways deregulated in common between the hippocampus and the striatum (Fig 6F).

## Discussion

To achieve improved phenotypic resolution of 16p11.2 BP4-BP5 rearrangement syndromes, we engineered and characterized novel mouse models carrying a deletion and/or duplication of the *Sult1a1-Spn* region and evaluated the genetic background effect on phenotype. Compared with mouse models described previously [21, 22] and as shown in Fig 1, our mouse models carry accurate rearrangements corresponding to the 16p11.2 BP4-BP5 syntenic region and do not show hearing deficits. We carried out a comprehensive battery of tests with these mouse models and unravelled the impact of genetic rearrangements on several functions affected in human patients, including cognition, repetitive behaviors, synaptic function, skull volume, and metabolism without a major impact of the genetic background. Compared to previous studies, we addressed new behavioral and metabolic paradigms, and highlighted the occurrence of alterations with opposite directions associated with the deletion or the duplication of the 16p11.2 syntenic region. Most of them were restored to normal levels in the pseudo-disomic mice carrying the deletion and the duplication (Del/Dup, Fig 2).

The *Del/+* and *Dup/+* animals were first generated separately on an inbred C57BL/6N genetic background. Independent cohorts of animals were subjected to two complementary behavioral pipelines to evaluate a range of parameters that can be related to human neuropsychiatric disorders. The *Del/+* mice displayed hyperactivity and recognition memory deficits, whereas the *Dup/+* mice displayed reciprocal phenotypes. Interestingly, *Del/+* mice also showed stereotypic behaviors (jumping and excessive climbing) commonly found in mouse models for autism [46]. No phenotypes related to schizophrenia, depression, or epilepsy were detected in our mouse models; mutant and control littermates showed similar capacities in the prepulse inhibition, sucrose preference, and pentylenetetrazol sensitivity tests (S2 and S6 Tables). We generated an "all-mutant" cohort by crossing *Del/+* with *Dup/+* animals to compare the *Del/+* and *Dup/+* mutant mice with a single group of wt animals and also to characterize the *Del/Dup* animals carrying two copies of the *Sult1a1-Spn* region on a single chromosome. Using this cohort, we confirmed the phenotypes for activity and recognition memory observed previously in the two groups of mutant mice bred separately (Fig 2, S4 and S5 Tables). Motor coordination and grip strength phenotypes were observed in both *Del/+* and *Dup/+* animals. During the evaluation of *in situ* TA isometric contraction in response to nerve stimulation, similar muscle force was measured between wt and mutant mice with (S8C–S8E Fig). In addition, we observed normal fiber size and normal succinate dehydrogenase (SDH) activities in TA muscle (S8F Fig) suggesting that these grip strength phenotypes in both genotypes were not due to severe muscular dysfunction. The ambiguity of *Del/+* results with improvement trends in the rotarod test and obvious deficits in the notched bar test suggested an important bias related to activity alterations of mutant mice.

We were particularly interested in altered learning and memory observed in mutant mice, which can be linked to intellectual disability and memory impairment found in humans carrying 16p11.2 BP4-BP5 CNVs [7, 9]. Thus, we performed the novel object recognition task using two retention delays: 30 min and 3 h. With the shorter delay, the *Del/+* mice showed deficits in recognition memory. With the longer delay, similar to the results in two separate mutant cohorts, the *Del/+* mice and the *Dup/+* mice displayed deficits and improvements, respectively, of novel object discrimination. Notably, the *Del/Dup* animals had similar phenotypes as the *Dup/+* animals and showed recognition memory improvements in comparison with wt mice. This result suggested that the *Dup/+* memory improvement phenotype might be linked to DNA structure changes associated with the duplication of the *Sult1a1-Spn* region. Except for this assay, the *Del/Dup* compound mutants did not display changes in any of the other phenotypes compared to the controls.

Among the series of synaptic transmission parameters measured in acutely prepared hippocampal slices, LTP was the only parameter to be statistically lower in *Dup/+* mice (Fig 4). We did not observe trends for opposite phenotypes in slices from *Dup/+* and *Del/+*. Moreover, LTP in *Del/+* mice was also nominally lower than in wt animals. Similarly, amplitudes of fEPSPs obtained in response to strong stimuli in slices from both *Del/+* and *Dup/+* mice were nominally lower than in slices from wt animals. The lack of statistically significant alterations in the input-output relationship and paired-pulse facilitation in the three lines of mutant mice suggested that despite the observation that several genes were affected by the 16p11.2 copy-number variations, basal synaptic transmission and short-term synaptic plasticity in a conventional hippocampal synapse were resistant to these changes. Of note, a similar lack of these phenotypes was noted recently in 16p11.2 *Del/+* mice by Tian et al. (2015) [47]. Our electrophysiological data were obtained on a modest number of animals and larger cohorts will be required to confirm our observations, particularly with respect to enhanced hippocampal LTP phenotype in *Dup/+* mice. At the same time, it is conceivable that behavioral and cognitive alterations in mice with 16p11.2 CNVs may be accounted for by changes in brain circuits other than the hippocampus. For instance, changes in dopaminergic signaling and striatal electrophysiology may underlie the lack of habituation observed in reaction to novel objects [22].

**Fig 7:**
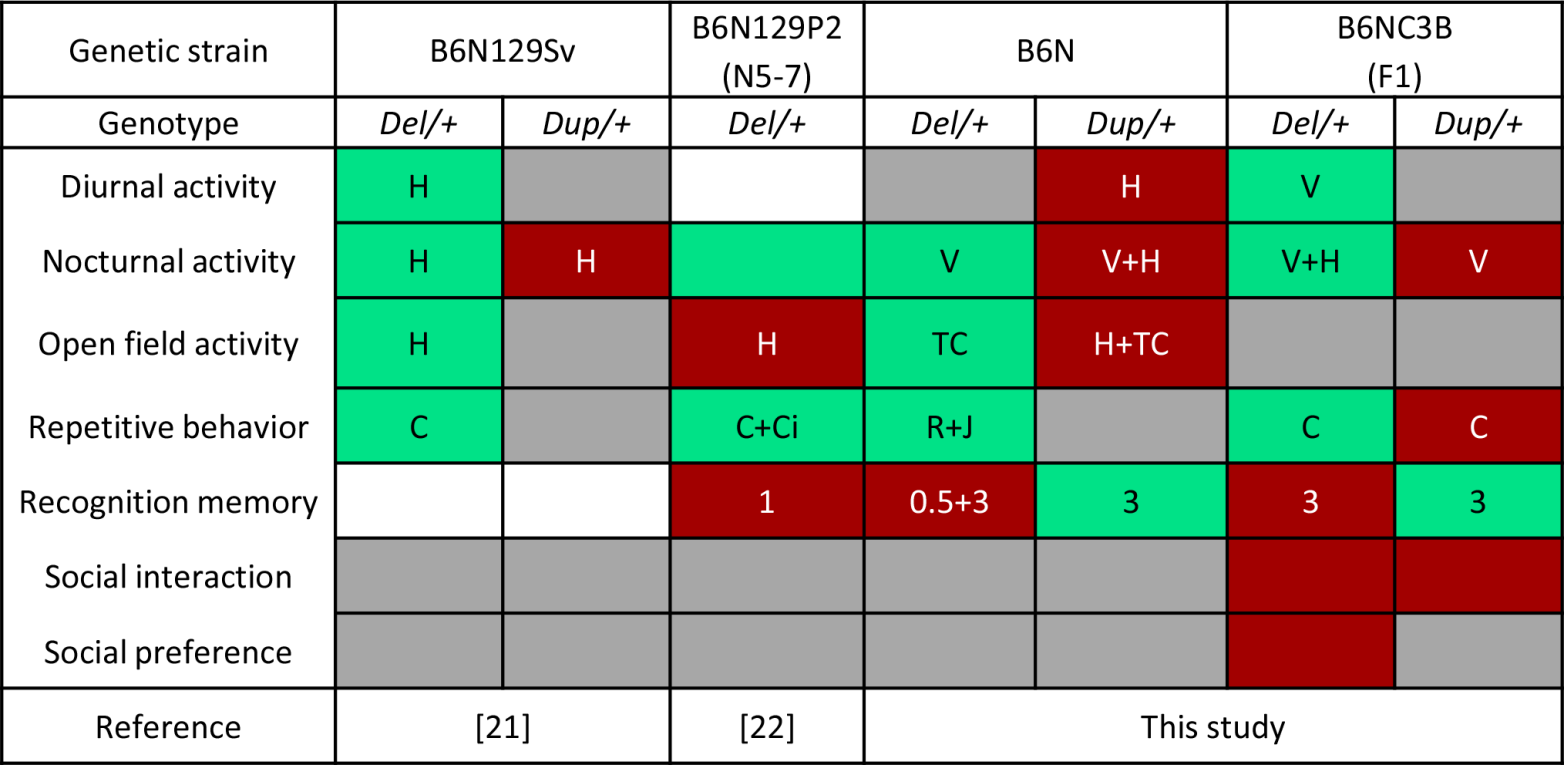
Behavioral map of phenotypes observed in mice carrying deletions and duplications of *Slx1b-Sept1* [21], *Coro1a-Spn* [22] and *Sult1a1-Spn* (this study) genetic intervals on hybrid and inbred genetic backgrounds. Common phenotypes were found for circadian locomotor activity and repetitive behaviors in the 3 mouse models on different genetic background. Recognition memory was not investigated for the *Slx1b-Sept1* models. Mice carrying the *Coro1a-Spn* deletion displayed an impairment of the object recognition memory (1 h retention delay) similar to *Sult1a1-Spn Del/+* mice that exhibited deficits with 30 min and 3 h retention delays. Changes in open field locomotor activity levels of the *Del/+* mice were inconsistent between the different studies. In the present study, mice carrying rearrangements for the *Sult1a1-Spn* region bred on a hybrid genetic background displayed lower levels of social interaction and mice carrying the deletion also showed an absence of social preference for the new individual in the three-chamber sociability test. Phenotype with a significant higher, or respectively lower, level in mutant compared to wt are indicated in green, or in red. When no difference in mutant and wt has been observed, the result is indicated with a grey cell while it is shown with a white box when the phenotype was not investigated. C: Climbing; Ci: Circling; H: Horizontal activity; J: Jumping; R: Rearing; TC: Time in Center; V: Vertical activity; Retention delays 0.5: 30 min; 1: 1h; 3: 3h

Differing from previous studies, we examined the influence of genetic background on the observed phenotypes. In addition to the characterization of inbred mouse models on a C57BL6/N genetic background, we generated and characterized *Del/+* and *Dup/+* animals on a F1 C57BL/6N×C3B hybrid genetic background. The presence of one copy of C3B alleles did not alter the main phenotypes observed in the 16p11.2 CNV models generated in the study (Figs 2 and 3). The nocturnal and diurnal hyperactivity, object recognition defect and repetitive behaviors of *Del/+* mouse models have been observed in both the C57BL/6N and C57BL/6N ×C3B genetic backgrounds and across different laboratories (Fig 7). Those phenotypes are highly reproducible, as they have been observed at least in two independent research centers. We propose that they represent robust and consistent core phenotypes of the 16p11.2 deletion mouse models. The higher and lower levels of climbing activity in the *Del/+* and *Dup/+* mice respectively were found on both inbred and hybrid genetic backgrounds. Horev *et al*. found similar climbing phenotypes in their *Del/+* and *Dup/+* models that were maintained on a C57BL/6N×129Sv hybrid background [21] (Fig 7). Notably, 24% of children with the 16p11.2 BP4-BP5 deletion had a diagnosis of ASD with some level of restricted and repetitive behavior reported [7]; such phenotypes were also observed in the *Del/+* mouse models. Some individuals carrying 16p11.2 CNVs present with attention-deficit/hyperactivity disorder [7, 11] and bipolar disorders [48, 49], which support a relation between 16p11.2 copy number and the regulation of activity and attention. The use of a C57BL/6N×C3B hybrid genetic background increased considerably memory capacities of wt animals. Similar to what we observed for the inbred genetic background, we found recognition memory deficits for *Del/+* mice and improvements for *Dup/+* mice on the hybrid background in the new object recognition task with 3 hours of retention delay. Since the use of the hybrid background reduces the activity phenotypes of *Del/+* and *Dup/+* mice, these results indicated that the recognition memory phenotypes seen on an inbred background were not due to an activity alteration, but rather, were the consequence of a diminished interest for the objects. In addition, *Dup/+* mice also displayed enhanced working memory performance in the Y-maze alternation task (S7 Table), suggesting that duplication of the *Sult1a1-Spn* region impacts primarily short-term memory processes. Portmann *et al*. performed the same recognition memory experiment with a retention delay of 1 hour. The authors also found an important deficit in their *Del/+* model maintained on a N5-7 C57BL/6N×129P2 hybrid genetic background [22] (Fig 7). Taken together, these experiments performed by independent research groups confirm the robustness of the activity and recognition memory phenotypes of the 16p11.2 CNV mouse models. Moreover, a recent study looking at the neuropsychological profile of 16p11.2 BP4-BP5 CNVs carriers shows that duplication carriers outperform intrafamilial controls on a verbal memory task, with the same IQ level [50]. This observation correlates with the higher performance of the mouse duplication carrier in the object recognition paradigm.

Comparison of the phenotypes induced by the 16p11.2 deletion on inbred C57BL/6N and hybrid C57BL/6N×C3B genetic backgrounds revealed striking differences. The embryonic lethality observed in the transmission of the deleted chromosome was completely abolished in the F1 cross (Table 1). The body weight of deletion carrier neonates at birth was normal in the hybrid background (S1C Fig) but the retarded growth was still observed later with a reduced body weight of adult *Del/+* animals observed in both backgrounds (S1D Fig). The loss of the neonatal lethality in the C57BL/6N×C3B *Del/+* mice has already been observed in other studies [51]. We compared the polymorphisms in the *Sult1a1-Spn* region between several mouse strains but we did not find significant differences, suggesting a possible contribution of a recessive allele located in *trans* outside of the 16p11.2 homologous region. An attenuation of the horizontal exploration activity was also observed in the F1 genetic background with the loss of phenotypes in the open field observed in animals with the inbred background (S4A and S4B Fig). In the C57BL/6N×C3B hybrid genetic background, the level of social behavior of wt animals was significantly increased, which allowed us to observe an obvious diminution of social interaction in both *Del/+* and the *Dup/+* mice (S4D Fig). As such, the C57BL/6N genetic background is less permissive to allow the detection of social interaction phenotypes. To confirm that the mutant animals did not present any olfactory deficits, we performed social odor and non-social odor tests and we found no difference between wt and mutant animals (S6C and S6D Fig). Social interaction deficits found in *Del/+* and *Dup/+* can be considered as a read-out for the autistic traits found in humans carrying deletions or duplications of the 16p11.2 BP4-BP5 region [3, 5]. The reduction in social interaction observed for mutant mice on the C57BL/6N×C3B is the first phenotype following the same direction for both *Del/+* and *Dup/+* animals. The presence of this phenotype on the C57BL/6N×C3B hybrid background but not on the C57BL/6N inbred background nor on the C57BL/6N×129Sv or C57BL/6N×P2 hybrid genetic backgrounds reported by Horev *et al*. and Portmann *et al*. respectively [21, 22], suggests that the influence of the genetic background is contributory to the manifestation of social behavior deficits induced by 16p11.2 CNV. Therefore, the genetic background has to be taken into account for the design of the *in vivo* modeling of other neuropsychiatric disorders [52, 53].

Because individuals carrying rearrangements at the 16p11.2 BP4-BP5 locus display extreme BMI alterations (lean or obese), we evaluated the influence of *Sult1a1-Spn* copy number on the weight and metabolism in our mutant animals. On an inbred genetic background, we observed a significant reduction of body weight, size and adipogenesis in the *Del/+* animals under a standard diet, which is consistent with previous studies [21, 22]. During the high-fat diet challenge, the *Del/+* metabolic phenotypes were similar to the normal diet, and we also observed opposite phenotypes in the *Dup/+* mice, including an increase in body weight and adipogenesis (Fig 5). Moreover, analysis of glucose clearance and endocrinological parameters revealed alterations of metabolism in the *Del/+* and *Dup/+* mice. Interestingly, on a C57BL/6N×C3B hybrid background, we observed similar phenotypes under standard (S7 Fig) and high-fat diets (S9 Table). The opposite effects of *Del/+* and *Dup/+* animals on body weight and adipogenesis are independent of the diet and of the genetic background, which suggests that rearrangements of the 16p11.2 homologous region induce robust metabolic alterations. Strikingly, our data indicated that weight and adiposity phenotypes are opposite between humans and mice carrying the 16p11.2 BP4-BP5 rearrangements. In humans, the 16p11.2 BP4-BP5 deletion is associated with obesity and hyperphagia [17], whereas reciprocal duplication is associated with lower weight and restrictive eating behavior [1]. More recently, data collected from young individuals with 16p11.2 deletion report that satiety response is altered before the onset of obesity [54], and should be a strong contributor to the energy imbalance in 16p11.2 CNVs in human. In comparison with their wt littermates, *Del/+* and *Dup/+* mice displayed normal food and water consumption (S2 Fig). No deregulation of the *Sh2b1* gene, a candidate located upstream of the BP4-BP5 region, was found in the liver, the cerebellum, the striatum or the hippocampus of the *Del/+* and *Dup/+* animals. In addition, we did not find any significant expression level changes for the genes located around the *Sult1a1-Spn* rearranged region. Similarly, both *Del/+* and *Dup/+* mouse models show skull shape alterations, however, microcephaly is observed in the *Del/+* mice (S8 Fig), in contrast to humans carrying the 16p11.2 deletion, who display macrocephaly. Looking at the 16p11.2 regions in the human and the mouse genomes, several changes occurred during primate evolution with the duplication of the low copy repeat region where the breakpoints take place and the general organization of the neighboring regions which differs from mouse (https://genome.ucsc.edu/). Such local changes may influence the outcome of the deletion and the duplication of the 16p11.2 region in the two organisms.

The opposite phenotypes described in this study suggest the implication of dosage-sensitive gene(s) within and/or outside the BP4-BP5 region that could influence mouse activity, recognition memory and metabolism. To test this possibility, we analysed transcriptome profiles of *Del/+*, *Dup/+* and wt animals in three brain regions and the liver. Overall, we found that the majority of genes within the *Sult1a1-Spn* region were sensitive to gene dosage. Rearrangements of *Sult1a1-Spn* region had a low impact on the whole genome transcriptome in the hippocampus, suggesting that dosage-sensitive genes leading to opposite phenotypes may be located in the 16p11.2 syntenic region. Principal component analysis suggested further that the effect of the deletion on the expression of the genes in the *Sult1a1-Spn* interval was stronger, as reported previously [21]. This result may indicate why behavioral, anatomic, and metabolic phenotypes are stronger in *Del/+* animals compared to the opposite phenotypes of the *Dup/+* mice. Our GSEA analysis revealed that the genes found “upregulated in the *nucleus accumbens* after cocaine treatment” were also upregulated in the striatum of *Del/+* mice, whereas they were downregulated in the striatum of *Dup/+* mice. These results thus indicated an alteration in the reward system, which could contribute to the behavioral phenotypes observed in our study. A large number of pathways were found altered in the striatum compared to other brain regions, suggesting a major impact of the 16p11.2 genetic dosage on the striatum that could explain repetitive behaviors and cognition defects observed in the 16p11.2 mutant mice. In addition, the analysis pinpointed a misregulation of genes involved in adipogenesis and obesity [55, 56] and contributing to the Reactome cholesterol biosynthesis in the liver of the *Del/+* and *Dup/+* mice which could explain the weight phenotypes observed in the mutant animals. We also found that the genes “down-regulated in liver tissue upon knockout of *Hnf1alpha”* were upregulated in the liver of *Del/+* mice, whereas they were downregulated in the liver of *Dup/+* mice. HNF1A is known to regulate numerous hepatic genes and heterozygous *HNF1A* mutations cause pancreatic-islet beta-cell dysfunction and monogenic diabetes [57, 58]. *Hnf1a* deficiency in mice leads to highly-specific changes in the expression of genes involved in key functions of both islets and liver [44, 59]. In humans HNF1A deficiency is the genetic cause Maturity-Onset Diabetes of the Young type 3 (MODY3). Such changes in the *Hnf1a-*dependent pathway most likely contribute to the metabolic phenotypes found in *Del/+* and *Dup/+* animals. Interestingly, differences exist in MODY3 humans versus mouse models; in the mouse, ablation of two *Hnf1a* alleles are required to induce a phenotype whereas only one copy of *HNF1A* is affected in human cases. These differences might explain the differences observed in the obesity phenotypes found in mouse compared to human.

Together, our results demonstrated that pathways mediating intellectual disability in carriers of the 16p11.2 deletion or duplication might be linked directly to altered expression of genes encompassed within the 16p11.2 BP4-BP5 region. This region contains several candidate genes for intellectual disability and neuropsychiatric disorders including *DOC2A*, *KCTD13*, *SEZ6L2*, and *TAOK2*. *DOC2A* (Double C2-like domains Alpha) encodes a synaptic vesicle-associated Ca^2+^ -binding protein. Mouse studies revealed that Doc2α interacts with Munc13, and is implicated in Ca^2+^-dependent neurotransmitter release [60]. *Doc2α* mutant mice show impairment in long-term potentiation and passive avoidance tasks, pinpointing a potential contribution of Doc2α to memory formation [61]. *KCTD13* (Potassium Channel Tetramerization Domain containing 13) encodes PDIP1 (polymerase delta-interacting protein 1) which interacts with the proliferating cell nuclear antigen and therefore might have a role in the regulation of cell cycle during neurogenesis [62]. A study in zebrafish models revealed that overexpression of the human *KCTD13* transcript in embryos induces microcephaly, whereas knockdown of endogenous *kctd13* by morpholino antisense oligonucleotides leads to macrocephaly, recapitulating the mirrored phenotype (i.e. head circumference) seen in 16p11.2 humans [63]. *SEZ6L2* (Seizure related 6 homolog (mouse)-Like 2) encodes a cell surface protein that has a strong homology with SRPX2 (Sushi-repeat-containing protein, X-linked), mutations in which cause epilepsy and language disorders [64]. In addition, association between a *SEZ6L2* coding variant and ASD was suggested but is still controversial [65]. *TAOK2* (Thousand-And-One-amino acid Kinase 2) encodes a serine/threonine kinase that activates mitogen-activated protein kinase (MAPK) pathways to regulate gene transcription. TAOK2 interacts with semaphorin 3A receptor neuropilin 1, which regulates basal dendrite arborization. Recently, TAOK2 has been shown to play a role in basal dendrite formation in mouse cortical neurons [66]. Taken together these data suggest a possible contribution of these genes in the recognition memory and activity phenotypes observed in *Del/+* and *Dup/+* mouse models. To test this possibility, *Dup/+* mice crossed with each of the heterozygous knockout mouse models for each of these candidate genes would help to determine whether the restoration of a normal copy number rescues the recognition memory and hypoactivity of *Dup/+* mouse model. Although elegant and definitive, this approach is not time and cost-efficient.

While most of the defects were stable and robust in our conditions or in other studies [21, 22], the 16p11.2 CNV affects the transcriptional profile of multiple pathways (a total of 246 and 169 misregulated in the striatum and in the liver, respectively; S10 Table) supporting the hypothesis of a broader genome wide transcriptional effect of the 16p11.2 gene dosage imbalance. Our study highlights the benefit of combining inbred and hybrid genetic background studies in mouse models of human syndromes, particularly when cases show symptoms of incomplete penetrance. In fact, the C57BL/6N background potentiates the activity and weight phenotypes of *Del/+* and *Dup/+* mice whereas the mixed C57BL/6N and C3B backgrounds reveals recognition memory phenotypes and social interaction deficits. We showed for the first time social interaction deficits of *Del/+* and *Dup/+* mice on a C57BL/6N×C3B hybrid background, which is notable due to the observation that 16p11.2 BP4-BP5 rearrangements are one the most common CNVs found in individuals with autism [3–7]. These novel mouse models for the 16p11.2 CNV will thus facilitate the identification of candidate genes responsible for memory and activity symptoms observed in 16p11.2 CNV carriers and could eventually be utilized to test small molecules of therapeutic benefit.

## Methods

### Mouse lines, genotyping and ethics statement

16p11.2Yah mouse models were generated through Cre-LoxP *in vivo* recombination using a mouse line carrying two loxP sites inserted upstream of *Sult1a1* and downstream from *Spn* genes in a *trans* configuration. Deletion of the *Sult1a1–Spn* region, Del(7*Sult1a1-Spn*)6Yah, referred as *Del/+*, was identified by PCR using primers Fwd1 (5’-CCTGTGTGTATTCTCAGCCTCAGGATG-3’) and Rev2 (5’-GGACACACAGGAGAGCTATCCAGGTC-3’). Duplication of the same region, Dp(7*Sult1a1-Spn*)7Yah (here *Dup/+*) was identified using primers Fwd2 (5’-ACTGCAGCCCGTCACCTAACTTCTT-3’) and Rev1 (5’-GGACACACAGGAGAGCTATCCAGGTC-3’). The wt allele was identified using Fwd1 and Rev1 primers. The PCR reactions gave deletion, duplication and wt products of 500 bp, 461 bp and 330 bp, respectively (Fig 1C). All mice were genotyped by PCR using the following program: 95°C /5 min; 35× (95°C/30 s, 65°C/30 s, 70°C/1 min), 70°C/5 min. All mouse experimental procedures were approved by the local ethics committee, Com’Eth (n°17) under the accreditation number 2012–069. YH was the principal investigator of this study (accreditation 67–369). The mouse lines are available through the INFRAFRONTIER/European Mouse Mutant Archive (EM:06133 and EM:06134).

### Viability tests

Viability tests were performed to study causes of *Del/+* lethality and to evaluate the weight of animals at birth. Fetuses were collected at embryonic day 18.5 (E18.5) to monitor their capacity to survive at birth. The fetuses were weighed, placed on a warm plate at 37°C and rolled gently to stimulate them to breathe. At 30 min after extraction, the numbers of breathing animals versus cyanotic and lethargic animals were counted. Tail samples were collected for genotyping. We isolated 37 wt and 32 *Del/+* fetuses on the C57BL/6N background from 7 pregnant wt females mated to *Del/+* males. 24 wt and 30 *Del/+* fetuses on the C57BL/6NxC3B background were isolated from 7 pregnant wt C3B females mated to *Del/+* C57BL/6N males. Finally, we also studied 24 wt and 27 *Dup/+* fetuses isolated from 6 pregnant wt females crossed to *Dup/+* males.

### Behavioral analysis

#### Experimental design

Behavioral studies were conducted between 12 and 20 weeks of age. This age range was chosen because of: 1) a goal of modeling the phenotypes found in young humans with the 16p11.2 CNV; 2) to avoid hyperactivity-like behavior related to young animals; and 3) to avoid age-increased passivity of older animals. To prevent estrous cycle interference, only male mice were used for behavioral experiments. All assessments were scored blind to genotype as recommended by the ARRIVE guidelines [67, 68]. We generated experimental animal cohorts by selecting mice from litters containing a minimum of two male pups. After weaning, animals were sorted by litters into 39 x 20 x 16 cm cages (Green Line, Techniplast, Italy) where they had free access to water and food (D04 chow diet, Safe, Augy, France). The temperature was maintained at 23±1°C, and the light cycle was controlled as 12 h light and 12 h dark (lights on at 7 am). Mice were transferred from the animal housing facility to the phenotyping area at the age of 10 weeks. On testing days, animals were transferred to experimental room antechambers 30 min before the start of the experiment. All experiments were performed between 8:00 AM and 2:00 PM. A resting period of 2 days to 1 week was used between two consecutive tests. The body weights of animals were recorded once a week (the same day at the same time) from the age of 12 weeks to the day of euthanasia.

First, we analyzed individual *Del/+* and *Dup/+* cohorts on B6N genetic background. Two cohorts (n = 15 wt, 11 *Del/+*; n = 13 wt, 11 *Dup/+*) were analyzed separately at the same age. Tests were administered in the following order: plus maze (12 weeks of age), open field (12 weeks), Y-maze (13 weeks), marble burying (13 weeks), forced swimming (14 weeks), prepulse inhibition (15 weeks), novel object recognition (16 weeks), three-chamber sociability (17 weeks), and repetitive behavior observation (18 weeks). Two other cohorts (n = 9 wt, 8 *Del/+*; n = 10 wt, 12 *Dup/+*) were passed under a second behavioral pipeline: circadian activity (13 weeks), new location recognition (14 weeks), social interaction (15 weeks), rotarod (16 weeks), grip test (16 weeks), Morris water maze (17–18 weeks), and pentylenetetrazol (PTZ) sensitivity (19 weeks). A resting period of 2 days to 1 week was used between 2 consecutive tests. Behavioral data of *Del/+* and *Dup/+* separate cohorts maintained on a B6N background are described in the supplementary information (S1 File).

To study the behavior of animals carrying the deletion and/or the duplication, we crossed *Del/+* with *Dup/+* animals and generated two *Del-Dup* cohorts of males (comprising 11 *Del/+*, 19 wt, 14 *Del/Dup*, and 19 *Dup/+* animals). We passed animals at the same age under the same behavioral pipeline and we pooled the data. Tests were administered in the following order: open field (12 weeks of age), sucrose preference (12 weeks), novel object recognition with 3 h retention delay (13 weeks), circadian activity (14 weeks), object recognition with 30 min retention delay (15 weeks), repetitive behavior observation (16 weeks), grip test (17 weeks), rotarod (17 weeks) and notched bar (18 weeks).

Finally, to study the influence of the genetic background, we generated *Del/+* and *Dup/+* separate cohorts on the C57BL/6NxC3B background (*n* = 12 wt, 14 *Del/+*; *n* = 13 wt, 12 *Dup/+*) by crossing C57BL/6N *Del/+* and *Dup/+* animals with C3B wt mice. Tests were administered in the following order: circadian activity (12 weeks of age), open field (13 weeks), new object recognition with 3 hour retention delay (13 weeks), Y-maze (14 weeks), social interaction (15 weeks), repetitive behavior observation (16 weeks), grip strength (17 weeks), rotarod (17 weeks), novel location recognition with 3 hour retention delay for the *Del/+* cohort (18 weeks), novel object recognition with 24 hour retention delay for the *Dup/+* cohort (18 weeks), three-chamber sociability (19 weeks), social odor discrimination and non-social odor discrimination tests (20 weeks).

Circadian activity, open-field, repetitive behavior observation, novel object recognition, social interaction, rotarod, and notched bar tests are described in the main manuscript. Elevated plus maze, Morris water maze, object location recognition, Y-maze, marble burying, three-chamber sociability, forced swimming, prepulse inhibition, PTZ sensibility, sucrose preference, grip strength, social odor discrimination, non-social odour discrimination and auditory brain responses tests are described in the supplementary information (S1 File).

Circadian activity (AC) was measured to assess spontaneous activity and feeding behavior over the complete light/dark cycle. Testing was performed in individual cages (11 x 21 x 18 cm^3^) fitted with infrared captors linked to an electronic interface (Imetronic, France) that provided automated measures of position and locomotor activity. Mice were put into cages at 11 am on the first day and removed on the next day at 7 pm. The light cycle was controlled as 12 h light and 12 h dark (lights on at 7 am). The 32 hours of testing were divided into three different phases: the habituation phase (from 11 am to 7 pm on the first day); the night/dark phase (from 7 pm on the first day to 7 am on the second day); and the day/light phase (from 7 am to 7 pm on the second day). Feeding behavior was evaluated using an automated lickometer and a 20 mg pellet feeder (Test Diet, Hoffman La-Roche).

Open-field (OF) was used to evaluate exploration behavior. Mice were tested in automated open fields (44.3 x 44.3 x 16.8 cm) made of PVC with transparent walls and a black floor, and covered with translucent PVC (Panlab, Barcelona, Spain). The open field arena was divided into central and peripheral regions and was homogeneously illuminated at 150 Lux. Each mouse was placed on the periphery of the open field and allowed to explore the apparatus freely for 30 min. The distance travelled, the number of rears and time spent in the central and peripheral parts of the arena were recorded over the test session.

For studying repetitive behavior, mice were put individually into dimly lit (60 Lux) clean home-cages without pellets or water bottle. The occurrence of repetitive behaviors (rearing, jumping, climbing, digging, grooming) was observed for 10 min and scored using an ethological keyboard (Viewpoint, Labwatcher, France).

The novel object recognition (NOR) memory is based on the innate preference of rodents to explore novelty. The test was carried out in an open field arena as described [69]. On the first day, mice were habituated to the arena for 30 min at 60 Lux. On the following day, animals were submitted to the first 10-min acquisition trial during which they were individually placed in the presence of object A (marble or die) placed 10 cm away from one of the box corners. The exploration time of object A (when the animal’s snout was directed towards the object at a distance ≤1 cm) was recorded. A 10-min retention trial (second trial) was conducted 30 min, 3 h or 24 h later. The familiar object (object A) and the novel object (object B) were placed at a distance 10 cm from two open field corners (the distance between the two objects was approximately 20 cm) and the exploration time of these two objects was recorded. A discrimination index was defined as (*t*_B_/(*t*_A_ + *t*_B_)) × 100. All mice that did not explore the first object for more than 3 seconds during the acquisition trial were excluded from the analysis.

Social behaviors were tested first with the interaction task conducted as described [70]. In each session, 2 mice of the same genotype and similar body weight housed in different cages were put in an open field area for 10 min. The duration of sniffing and social behaviors were recorded during the 10-min session.

Sensorimotor coordination and balance were assessed with the rotarod test [71]. The apparatus (Biosed, France) is a rotating bar of 5 cm diameter (hard plastic materiel covered by grey rubber foam) on which mice are placed facing the direction of rotation. Animals were first habituated to stay on the rod for 30 seconds at a constant speed of 4 rotations per minute (rpm). This was followed by 3 training days with 4 trials per day. Mice were placed on an accelerating rod increasing from 4 rpm to 40 rpm in a 5 min period. The test was stopped when mice fell down from the rod or when they made more than one passive rotation. The latency to fall and the maximal speed before falling was recorded.

The notched bar was used to test hind limb coordination. Testing was performed as described [71]. The mice were trained with a bar consisting of a natural wooden piece 1.7 cm large and 50 cm long bearing terminal platforms of 6 cm × 6 cm. On the second day, mice were tested at 100 Lux with a notched bar of the same dimension that consisted of 12 platforms of 2 cm^2^ spaced out by 13 gaps of 2 cm^2^. Animals had to cross the notched bar twice for training and 15 times for the test. Each time a back paw went through the gap was considered as an error, and a global error percentage was calculated.

### Electrophysiology of hippocampal slices

An independent *Del-Dup* cohort including males and females was used for electrophysiology analysis (comprising 4 *Del/+*, 8 wt, 6 *Del/Dup*, and 4 *Dup/+* animals). All animals were 8–9 months old on the day of dissection. Investigators were blind to the genotype of mice until the end of all experiments within the groups. Acute hippocampal slices were used to record field excitatory post-synaptic potentials (fEPSPs), by the MEA60 electrophysiological suite (Multi Channel Systems, Reutlingen, FRG) as described [72, 73]. Further details are provided in the supplementary information (S1 File).

### Metabolic and biochemistry analysis on a high-fat diet

Since males were used for behavioral analyses, metabolic and biochemistry analysis were performed on females to both reduce the number of animals used and also because female mice are more prone to weight gain after metabolic challenges with an enriched diet. The separate *Del/+* and *Dup/+* cohorts on the B6N genetic background (*n* = 11 wt and 9 *Del/+*; *n* = 8 wt and 10 *Dup/+*) and a *Del/+* cohort on the C57BL/6NxC3B background (*n* = 8 wt and 10 *Del/+*) were put on a high-fat diet for metabolism and biochemical analyses.

Mice were transferred from the animal facility into the phenotyping area at the age of 4 weeks. Two to four animals were housed in one cage and fed with a standard chow diet (D04, Safe, USA) until the age of 5 weeks, when the diet was switched to a high fat/high carbohydrate diet (HFHCD, RD 12492, Research Diet, USA) until the end of the study (16 weeks). Body weights were recorded once a week from the age of 6 to 13 weeks. At the age of 11 weeks, mice were put individually into the TSE cages for 24 hours for the measurement of energy expenditure by indirect calorimetry. At the age of 12 weeks, an intra-peritoneal glucose tolerance test (IPGTT) was performed after overnight fasting. Body composition (lean, fat and free body fluid content) was evaluated in conscious mice by quantitative nuclear magnetic resonance (qNMR) at the age of 13 weeks. At the age of 15 weeks, blood was collected by retro-orbital puncture under isoflurane anesthesia for biochemistry, haematology and endocrine analysis. At the age of 16 weeks, animals of the *Del/+* cohort on the C57BL/6NxC3B background were put in individual cages for 48 h to collect feces for the measurement of energy content by bomb calorimetry.

Body composition analysis by qNMR was performed to give a precise assessment of the body composition for fat content, lean tissues and free body fluid by nuclear magnetic resonance apparatus and Minispec+ analyser (Bruker, Germany). The test was conducted during the light period in conscious, fed mice.

Energy expenditure was evaluated by indirect calorimetry that measured oxygen consumption with an open flow respirometric system (TSE System, Labmaster, Germany). Sensors measured the difference in CO_2_ and O_2_ concentrations in the air volumes flowing through control or animal cages. The amount of oxygen consumed over a given period of time could be calculated, as long as the air flow through the cage was known. The gas exchange data are expressed in Kcal/h/kg^0.75^. The system also monitored CO_2_ production, and we calculated the respiratory exchange ratio (RER = VCO_2_/VO_2_), which defines fuel preference between glucose vs lipid metabolism and heat production (Kcal/h/kg^0.75^). Following a 3 h acclimatization period, the experiment was performed over 21 h: from 17:00 on the first day to 11:00 on the day after, at an ambient temperature (21°C ± 2) under a 7:00/19:00 light/dark cycle.

The intraperitoneal glucose tolerance test (IPGTT) was used to assess the regulation of glycaemia after induced hyperglycaemia by injecting a standardized glucose bolus (2 g/kg). A glucose solution was administered by intraperitoneal injection. Blood glucose was measured in a drop of blood collected from the tail at different time points over 120 min after injection using a blood glucose monitor and glucose test strips (Roche Diagnostics, Accu-Chek, France). The test was conducted during the light period after 16 h of overnight fasting.

Blood was collected by retro-orbital puncture under isoflurane anaesthesia after 4 h fasting. Blood chemistry analysis was performed using a chemistry analyser (Olympus AU-400, USA) and commercial reagents (Olympus Diagnostica GmbH, Lismeehan, Ireland). The following parameters were measured: total cholesterol, HDL and LDL cholesterol, triglycerides, free fatty acids, total proteins, albumin, calcium, phosphorus, glucose, urea, creatinine, total proteins, and albumin. Plasma insulin and leptin levels were measured with a BioPlex analyser using Milliplex beads Panel (Millipore, USA). Plasma adiponectin levels were measured using a mouse adiponectin ELISA kit f (R&D Systems, USA).

Fecal energy content was estimated by bomb calorimetry. The feces were collected over 48 h from mice that were housed individually. The energy content of fecal samples was evaluated in a bomb calorimeter (C503 control, IKA, USA). The samples were burned in an oxygen-rich atmosphere inside a sealed chamber surrounded by a jacket containing a known volume of water. The rise in the water temperature was recorded and used to calculate the amount of heat produced. The assay was performed on feces to evaluate indirectly the energy digested by mice and the intestinal function. The energy digested was calculated by the difference between the total calories ingested and excreted in feces.

### Craniofacial analysis

Craniums of 13 week-old female mice (*n* = 10 wt and 10 *Del/+; n* = 8 wt and 8 *Dup/+*) of C57BL/6N genetic background were stored in 100% ethanol. Three-dimensional coordinates of 39 relevant cranial landmarks were recorded using Landmark software, and posterior comparisons were performed using the Euclidean distance matrix analysis (EDMA) with the WinEDMA software (version 1.0.1 beta). Further details are provided in supplementary information (S1 File).

### Transcriptome analysis

Expression profiling after RNA extraction was performed using an Affymetrix Mouse Gene *2*.*1* ST 24-Array Plate following the manufacturer's protocol. All arrays passed the standard Affymetrix quality control checks. Expression signals from 12 liver samples and 44 brain samples (12 cerebellum, 16 striatum, and 16 hippocampus) were quantile normalized separately. We applied a non-specific filter to discard probe sets with low signals (maximum signal lower than the median signal for all probe sets) or with low variance (lowest 25^th^ percentile of variance). Differentially expressed genes were defined using linear models as implemented in LIMMA v3.18.13 [74]. We fitted a dosage model where the 16p11.2 copy number variants (CNVs) were considered as a numerical variable, i.e., we assumed a dosage effect of the CNVs: gene_i ~ b0+ b1CNV CNV = -1,0,1. Gene Ontology (GO) analysis was performed using the library topGO v2.14.0. Gene Set Enrichment Analysis (GSEA) was conducted using the Broad Institute algorithm v2.1.0 [75, 76]. We used pre-ranked gene lists defined according to the LIMMA results based on the dosage model, and we utilized MSigDB C2 (c2.all.v4.0.symbols.gmt) to collect curated gene sets from online pathways, and publications in PubMed. The data are accessible through the NCBI’s Gene Expression Omnibus with the GEO Series accession number GSE66468.

### Statistical analysis

We assessed statistical significance using Sigma Plot software (Sigma). All acquired behavioral data were analysed using one-way or two-way ANOVA with repeated measures followed by Student’s *t*-test or Tukey’s *post-hoc* test as appropriate. Otherwise, the non-parametric Kruskal-Wallis analysis and Mann-Whitney *U*-test were used. Student’s *t*-tests were used to compare recognition index values to the chance level (50%). A Pearson’s chi-square test was used to evaluate mutant allele transmission. The data are represented as the mean ± standard error of the mean (s.e.m.) and the significance threshold was *P* < 0.05 unless otherwise indicated.

In electrophysiological experiments, input-output relationships were compared initially by mixed model repeated-measures ANOVA and the Bonferroni *post hoc* test implemented in Prism 5 (GraphPad Software, Inc., San Diego, CA, USA) using individual slice data as independent observations. Because several slices were recorded routinely from every mouse, the fEPSPmax, PPF and LTP values between wt and mutant mice were also compared using a two-way nested ANOVA design with genotype (group) and mice (sub-group) as factors (STATISTICA v. 10, StatSoft, Inc., Tulsa, OK, USA). To compare data obtained from *Del*/+, *Dup*/+ and *Del*/*Dup* mice to their litter-matched wt counterparts, we used *post hoc* Dunnett’s Multiple Comparison test on data from individual slices. Statistical effects were considered significant if *P* < 0.05. Graph plots and normalization were performed using OriginPro 8.5 software (OriginLab, Northampton, MA, USA). Throughout the text, the electrophysiological data are presented as the mean ± s.e.m. with *n* and *N* indicating the number of slices and mice, respectively.

## Supporting Information

S1 Fig Body weight, adiposity and body size evaluation of separated C57BL/6N *Del/+* and *Dup/+* cohorts on a standard diet. (A-B) *Del/+* and *Dup/+* cohorts on a C57BL/B6N genetic background. (C-F) *Del/+* and *Dup/+* cohorts on C57BL/B6NxC3B genetic background. (A, C) Weight of fetuses (g) at E18.5 embryonic stage. (B, D) Body mass (g) of adult mice evaluated over time. (E) Body fat percentage of 20-week old animals measured by qNMR. (F) Body length (distance from snout to tail basis) of 20-weeks animals. Data are represented as the mean + SEM. Student’s t-test, *P < 0.05, **P < 0.01, ***P < 0.001.
(TIF)

S2 Fig Behavioral characterization of C57BL/6N *Del/+* and *Dup/+* separated cohorts. (A) Circadian activity test. Graphs plot the ambulatory activity (count) and the vertical activity/rears (count) during dark and light phases. (B) Open field test. Distance travelled (m), vertical activity/rears (count) and percentage time spent in the central area over 30 min of testing. (C) Repetitive behavior. Counts of rearing, jumping, climbing and digging behaviors during 10 min of observation in a novel cage. (D) Social interaction test. Graph plots the duration of sniffing and following behaviors. (E) Novel object recognition test. Discrimination index was calculated as the ratio of time spent exploring the novel object vs the time spent exploring the familiar object in the choice trial. (F) Novel location recognition test. Discrimination index was calculated as the ratio of time spent exploring the displaced object vs the non-displaced object in the choice trial. (G) Motor coordination evaluation. Graphs plot the latency (s) that mice stayed on the rotating rod over 3 consecutive testing days. Data are represented as the mean + SEM. *P < 0.05, **P < 0.01 and ***P < 0.001, significantly different from their wt counterparts, Student’s t-test.
(TIF)

S3 Fig Spontaneous locomotor activity and feeding behavior of the C57BL/6N *Del-Dup* cohort during the circadian activity test. Patterns of locomotor activity (A) and vertical activity (B) profiles during 32-hours of test. *Dup/+* mice showed a global hypoactivity for ambulatory and vertical activity while Del/+ mice are more active. (C-D) Feeding behaviors. Water (C) and pellet (D) consumption during the 32-hours of testing. (E) Pellets lost by animals which passed through the holed ground. *Del/+* mice lost significantly more pellets compared to the other genotypes. Data are represented as the mean + SEM. ^#^ *P* < 0.05 vs all other groups. Mann Whitney U tests following a significant Kruskal Wallis analysis.
(TIF)

S4 Fig Exploratory activity of the C57BL/6N *Del-Dup* cohort during the open field test. (A) Distance travelled (m). Whereas wt and *Dup/+* mice showed a habituation and decreased their activity at the end of the test, *Del/+* and *Del/Dup* mice showed a constant activity. (B) Vertical activity (rears). All genotypes showed similar patterns of vertical activity without habituation. (C) Time in center (%). *Del/+* mice showed a marked increase of time spent in the arena center. Data are represented as the mean ± SEM. Student’s t-test, *P < 0.05, **P < 0.01.
(TIF)

S5 Fig Tibialis anterior (TA) muscle strength, endurance, and succinate dehydrogenase (SDH) staining of the C57BL/6N *Del-Dup* cohort. (A) Whole body weight (g) and TA weight (mg). (B) TA to body weight ratio. (C) Absolute maximal force of the TA muscle. (D) Specific maximal force of the TA. (E) Fatigue in TA muscle, measured as the time taken to reach 50% of maximum muscle force. (F) SDH histoenzymatic staining. Transverse cryosections (8 μm) of mouse skeletal muscles were prepared, fixed, and stained with succinate dehydrogenase (SDH). Data are represented as the mean + SEM. Tukey's test was applied following a significant one-way ANOVA, *** *P* < 0.001.
(TIF)

S6 Fig Hearing and Olfaction capacities of animals. (A-B) Hearing capacities of the C57BL/6N *Del-Dup* cohort in the auditory brain response test. No differences were observed between mutant and control littermates for both genotypes. (C-D) Olfaction capacities of the separated C57BL/6NxC3B *Del/+ and Dup/+* cohorts in the social (A) and non-social (B) odor discrimination tests. Data are represented as the mean ± SEM.
(TIF)

S7 Fig Metabolic and endocrinology analysis of the C57BL/6NxC3B *Del/+* cohort put on a high-fat diet at 5 weeks of age. (A) Evolution of body weight (g). (B) Body fat percentage measured by qNMR. (C) Energy expenditure (Kcal/h per kg^0.75 of body weight) during 12-hour dark and 12-hour light phases. (D) Intraperitoneal glucose tolerance test. Evolution of blood glucose (mg/dl) and glucose area under the curve (AUC) (min*mg/dl) (E) Endocrinology analysis. Leptin (ng/ml) and adiponectin (μg/ml) blood levels. (F) Blood chemistry analysis. Total cholesterol (μg/ml), glucose (mmol/l) and calcium (mmol/l) blood levels. (G) Bomb Calorimetry. Total daily energy excreted (Kcal). Data are represented as the mean + SEM. Student’s t-test, *P < 0.05, **P < 0.01, ***P < 0.001.
(TIF)

S8 Fig Craniofacial analysis of separated C57BL/6N *Del/+* and *Dup/+* cohorts. (A) Representative reconstructed 3D skull images and landmark distribution. Euclidian distances between the different landmarks allowed calculation of both the form (or size) difference (FD) and the shape difference (SD). Analysis revealed size reduction of the skull of *Del/+* animals (B) and no alteration of skull size of *Dup/+* animals (D). An alteration of skull shape was found for *Del/+* (C) and *Dup/+* (E) mice.
(TIF)

S1 Table Behavioral characterization of the separate *Del/+* and *Dup/+* cohorts on the C57BL/6N genetic background. Spontaneous circadian activity and feeding behaviors were assessed in the actimeter cages. *Del/+* mice showed hyperactivity whereas *Dup/+* mice showed hypoactivity during habituation (hab), dark and light phases. A considerable increase in the number of rears was observed for *Del/+* mice during the dark phase. No feeding behavior phenotypes were noted during this test. Exploratory activity and emotional reactivity to novelty was assessed in the open field test. In comparison with wild-type littermates, *Del/+* and *Dup/+* mice travelled longer and shorter distances during the 30 min of test, respectively. Vertical activity/rears were also increased for *Del/+* mice. Time spent in the center area, the most aversive part of the arena, was similar between mutants and controls. The elevated plus maze did not reveal any phenotypes. Observation of repetitive behaviors in a novel home cage during 10 min revealed a higher number of climbing, rearing, and jumping events in *Del/+* mice and a lower number of digging, climbing, and rearing events in *Dup/+* mice as compared to their wt counterparts. In comparison with controls, *Del/+* mice buried less marbles in the marble burying test. Overall, *Del/+* and *Dup/+* mice showed hyperactivity and hypoactivity phenotypes, respectively. No signs of anxiety were detected in mutant mice. Data are shown as the mean ± SEM. *P < 0.05, **P < 0.01 and ***P < 0.001, significantly different from their wt counterparts, Student’s t-test.
(DOCX)

S2 Table Characterization of cognitive and social behavior of *Del/+* and *Dup/+* cohorts on the C57BL/6N genetic background. Working memory was first assessed with the Y maze test, which did not reveal any phenotypes in mutant mice. Memory abilities were then evaluated with novel object and novel location recognition tasks. In the acquisition session (S1), no differences in object exploration were noted between genotypes. After a 3-hour delay, *Del/+* mice showed a recognition memory deficit for object location whereas *Dup/+* mice showed memory improvements for object identity and location. In the Morris water maze, no change of spatial learning and memory was observed. Mutant and control mice travelled the same distance and needed the same duration to find the hidden platform from the first day to the sixth and last training day (D6). In the probe test (PT), performed on the 7^th^ day, without the hidden platform, all mice spent the same percentage of time in the target quadrant. No phenotype was noted in the social interaction and three-chamber sociability tests. In the forced swim test, *Del/+* animals floated less than 2n mice, which may be due to the hyperactivity phenotype of these mice. Prepulse inhibition (PPI) tests did not reveal any abnormalities in acoustic startle reflex responses and sensorimotor gating capacities and the pentylenetetrazole (PTZ) sensitivity test did not reveal any difference in seizure susceptibility between genotypes. In the rotarod test, *Dup/+* mice did not show motor learning improvement with repeated testing (from the first day (D1) to the third day (D3)). Finally, the grip test indicated that *Del/+* and *Dup/+* mice showed stronger and weaker grip strength, respectively. Data are represented as the mean ± SEM. *P < 0.05, **P < 0.01 and ***P < 0.001, significantly different from wt counterparts, Student’s t-test.
(DOCX)

S3 Table Characterization of circadian activity, emotional reactivity and repetitive behaviors in the *Del-Dup* cohort on C57BL/6N genetic background. In the circadian activity test, *Del/+* mice showed vertical hyperactivity during dark phase whereas *Dup/+* mice showed vertical hypoactivity during light and dark phases and ambulatory hypoactivity during light phase (statistical values are summarized in S3 Table). During open field tests, *Dup/+* mice travelled a shorter distance and spent less time in arena center whereas *Del/+* mice spent more time in the arena center. Observation of repetitive behaviors revealed an increase in the level of rearing and jumping for *Del/+* mice. Data are represented as the mean ± SEM.
(DOCX)

S4 Table Behavioral characterization of the *Del-Dup* cohort on the C57BL/6N genetic background. Object recognition memory of mice was assessed with two retention delays, 30 min and 3-hours. *Del/+* mice showed short-term memory deficits at both delays (statistical values are summarized in S2 Table). *Dup/+* and *Del/Dup* mice showed improvement at the 3-hour delay. No anhedonia phenotype was observed in the sucrose preference test. No phenotype was detected in the social interaction test. During the rotarod test, *Del/+* mice showed trends for improvement whereas *Dup/+* and *Del/Dup* mice showed impairment trends (not significant). *Del/+* animals also showed impairments in the notched bar test. *Del/+* and *Dup/+* mice showed stronger and weaker grip strength in the grip test, respectively. Data are represented as the mean ± SEM.
(DOCX)

S5 Table Statistical values for the *Del-Dup* behavioral characterization on the C57BL/6N genetic background. The table summarizes post-hoc analysis of parameters for which analysis of variance revealed significant differences between genotypes. *p < 0.05, **p < 0.01 and ***p < 0.001, Tukey's test following a significant one-way ANOVA analysis. ^+^*p* < 0.05, ^++^*p* < 0.01 and ^+++^*p* < 0.001, Mann Whitney U tests following a significant Kruskal Wallis analysis.
(DOCX)

S6 Table Characterization of circadian activity, emotional reactivity and repetitive behaviors in *Del/+* and *Dup/+* cohorts on the C57BL/6NxC3B genetic background. In the circadian activity test, *Del/+* mice showed increased horizontal activity during the dark phase and enhanced vertical activity during the dark and the light phases whereas *Dup/+* mice showed reduced vertical activity during the dark phase. No behavioral phenotypes were detected in in the open field test. The marble burying test, which was only done for *Del/+* cohort, also did not reveal any phenotypes in mutant mice. Finally, observation of the animals in a novel home cage revealed increased and decreased level of climbing behavior in *Del/+* and *Dup/+* mice, respectively. NT: not tested. Data are shown as mean ± SEM. *p < 0.05, **P < 0.01 and ***P < 0.001, significantly different from their wt counterparts, Student’s t-test.
(DOCX)

S7 Table Behavioral characterization of *Del/+* and *Dup/+* cohorts on the C57BL/6NxC3B genetic background. Working memory was first assessed with the Y maze test, which revealed an improvement in *Dup/+* animals. Recognition memory was first evaluated with the novel object recognition task. In the acquisition session (S1), no difference in object exploration was noticed between genotypes. After a 3-hour delay, *Del/+* and *Dup/+* mice show memory impairment and improvement for object identity, respectively. We did the same test for the *Dup/+* cohort with a 24-hour delay and found no phenotype, suggesting that duplication of Sult1a1–Spn impacts mnemonic processes associated with short-term memory. With the *Del/+* cohort, we performed the novel place recognition task with a 3-hour delay and also found a memory deficit for object location. In the social interaction test, we found a decrease of sniffing behavior for *Del/+* mice and a decrease of sniffing and following behaviors for *Dup/+* animals. In the three-chamber sociability test, we did not observe differences in social interaction, but novel animal discrimination was significantly decreased for *Del/+* animals. No motor coordination phenotype was seen in rotarod and notched bar tests but *Del/+* and *Dup/+* mice still presented stronger and weaker grip strength in the grip test, respectively. NT: not tested. Data are shown as the mean ± SEM. *P < 0.05, **P < 0.01 and ***P < 0.001, significantly different from wt counterparts, Student’s t-test.
(DOCX)

S8 Table High-fat diet analysis of *Del/+* and *Dup/+* cohorts on the C57BL/6N genetic background. Animals were put on a high-fat diet at the age of 5 weeks. In comparison with controls, *Del/+* animals were underweight, shorter in size and deposited less fat whereas *Dup/+* mice were overweight, longer in size and displayed more adipose tissue. Indirect calorimetry showed increased levels of energy expenditure (EE) and oxygen consumption (OC) for *Del/+* mice during the dark phase. *Dup/+* mice presented a diminution of EE and OC during dark and light phases. In intraperitoneal glucose tolerance tests (IPGTT), *Del/+* mice showed faster glucose clearance whereas *Dup/+* mice presented similar glucose clearance in comparison to controls but had an increased glycemia before injection of glucose (T_0_ blood-glucose). Blood chemistry analysis did not reveal gross hematology changes except a diminution of free fatty acids level of *Del/+* mice. Finally, consistent with qNMR results, endocrinology analysis showed diminution of leptin and adiponectin level for *Del/+* mice and an increase of leptin levels for *Dup/+* mice. Data are shown as the mean ± SEM. *P < 0.05, **P < 0.01, ***P < 0.001, significantly different from wt counterparts, Student’s t-test.
(DOCX)

S9 Table High-fat diet analysis of *Del/+* cohort on the C57BL/6N-C3B genetic background. Animals were put on a high-fat diet at the age of 5 weeks. *Del/+* animals were underweight and displayed less fat than wild-type littermates. Indirect calorimetry showed an increase in the levels of energy expenditure (EE) and oxygen consumption (OC) for *Del/+* mice during dark and light phases. No difference of energy excreted in feces was found in bomb calorimetric analyses. During intraperitoneal glucose tolerance tests (IPGTT), *Del/+* mice showed faster glucose clearance but had an increased glycemia before injection of glucose (T_0_ blood-glucose). Blood chemistry analysis revealed an increased level of calcium and decreased levels of glucose and cholesterol for mutant mice. Finally, in consistent with qNMR results, endocrinology analysis showed diminution of leptin and adiponectin level for *Del/+* mice. Data are shown as the mean ± SEM. *P < 0.05, **P < 0.01, ***P < 0.001, significantly different from wt counterparts, Student’s t-test.
(DOCX)

S10 Table Gene set enrichment analysis. Our analysis considers the pre-ranked gene list according to the LIMMA results based on the dosage model. The gene set database used was the MSigDB C2 (c2.all.v4.0.symbols.gmt), which collects curated gene sets from online pathways, publications in PubMed, and the knowledge of domain experts. The table include gene sets dose-dependently upregulated (upregulated in the *Dup/+* mice and downregulated in *Del/+* mice) and downregulated (downregulated in the *Dup/+* mice and upregulated in *Del/+* mice). Data with an FDR below 25% are shown. CEREB: Cerebellum; DEREG: Deregulation; HIP: Hippocampus STRI: Striatum.
(DOCX)

S1 File Supplementary Information. Detailed protocols used in the study or for supplementary figures and tables are provided in the document.
(DOCX)
