# Supplementary figures and images for: Reciprocal Effects on Neurocognitive and Metabolic Phenotypes in Mouse Models of 16p11.2 Deletion and Duplication Syndromes
Source: PLoS Genet. 2016 Feb 12;12(2):e1005709. doi: 10.1371/journal.pgen.1005709 (PMC4752317; doi:10.1371/journal.pgen.1005709)

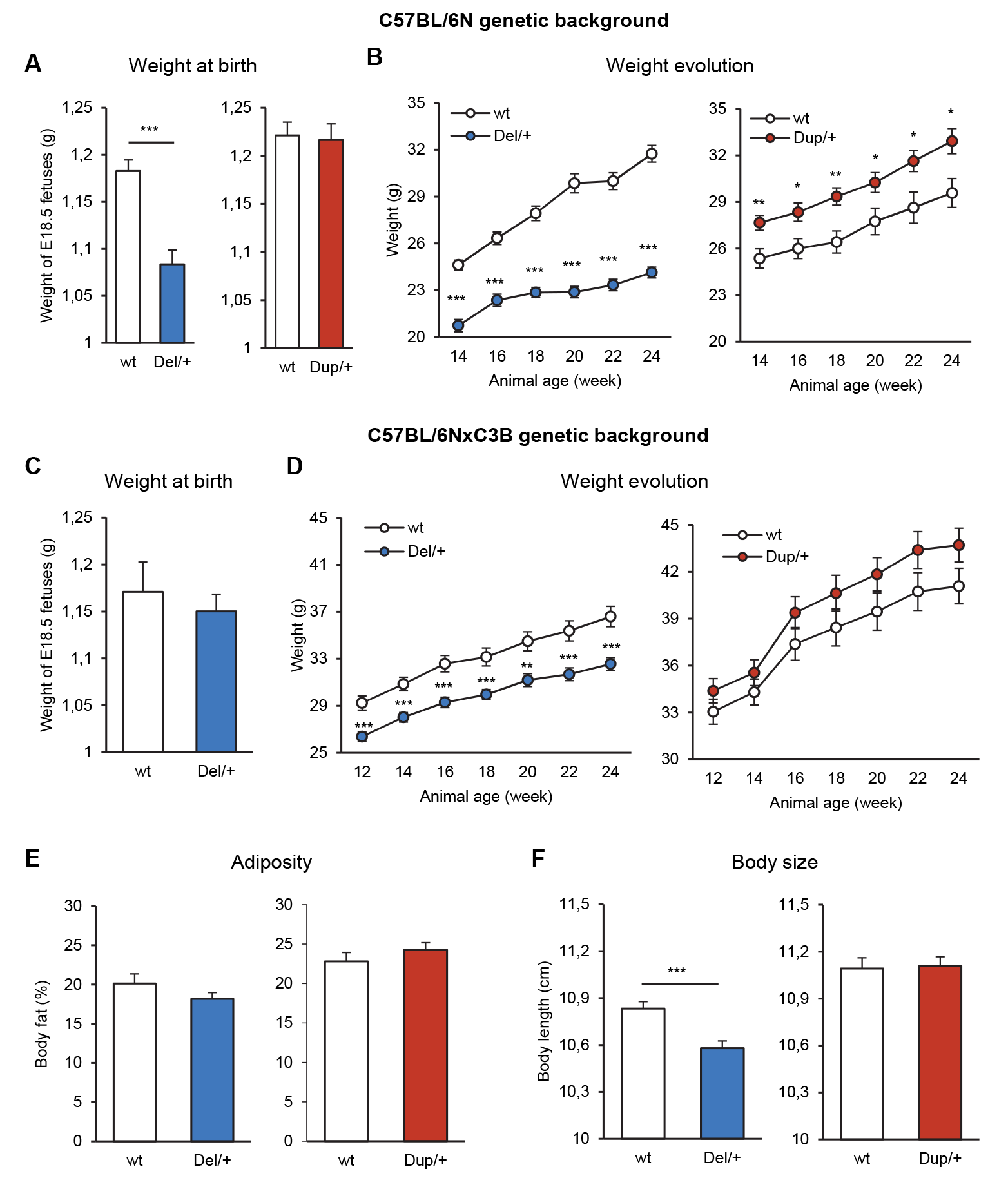

Supplement: S1 Fig — (A-B) Del/+ and Dup/+ cohorts on a C57BL/B6N genetic background. (C-F) Del/+ and Dup/+ cohorts on C57BL/B6NxC3B genetic background. (A, C) Weight of fetuses (g) at E18.5 embryonic stage. (B, D) Body mass (g) of adult mice evaluated over time. (E) Body fat percentage of 20-week old animals measured by qNMR. (F) Body length (distance from snout to tail basis) of 20-weeks animals. Data are represented as the mean + SEM. Student’s t-test, *P < 0.05, **P < 0.01, ***P < 0.001. (TIF) [file pgen.1005709.s001.tif]

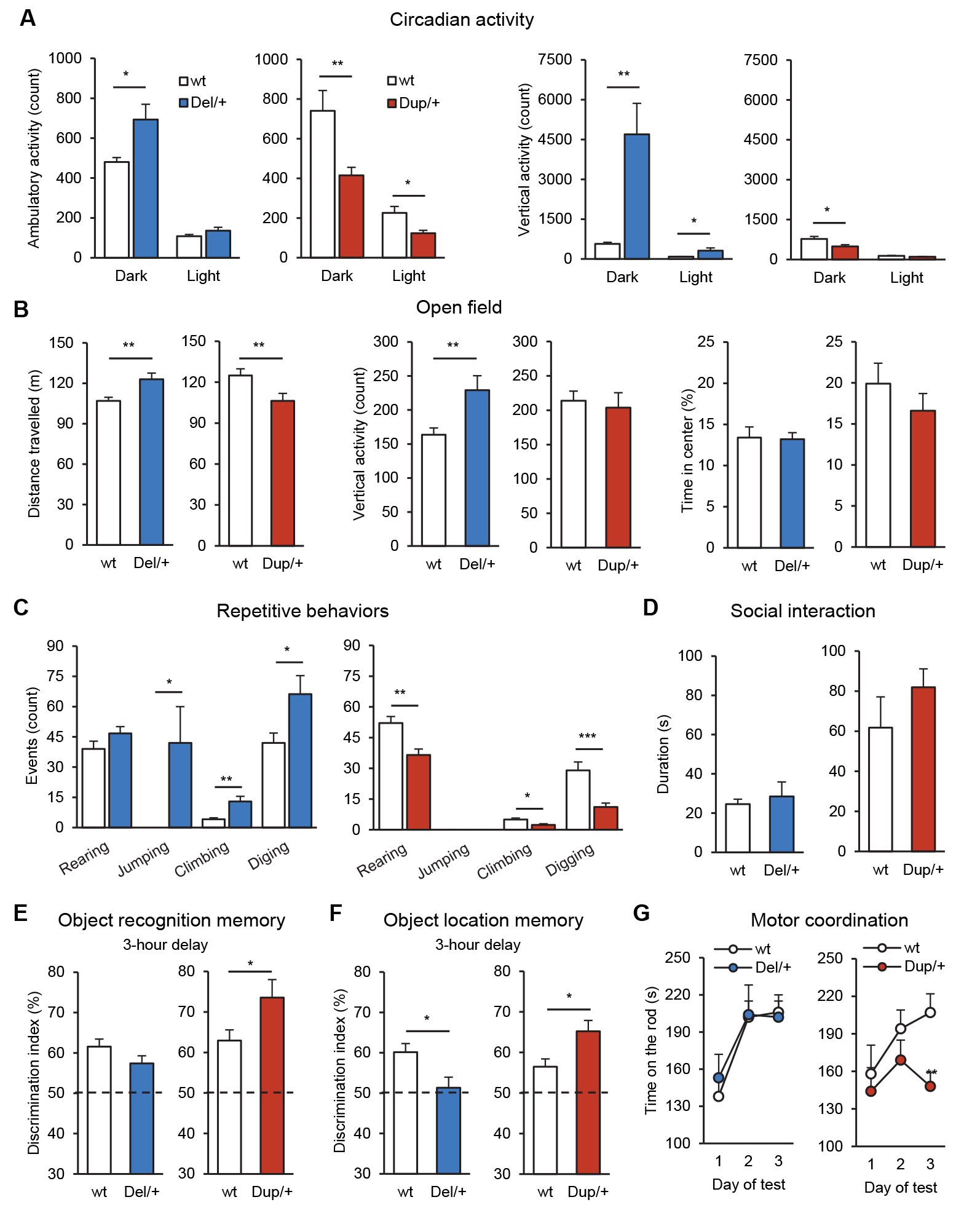

Supplement: S2 Fig — (A) Circadian activity test. Graphs plot the ambulatory activity (count) and the vertical activity/rears (count) during dark and light phases. (B) Open field test. Distance travelled (m), vertical activity/rears (count) and percentage time spent in the central area over 30 min of testing. (C) Repetitive behavior. Counts of rearing, jumping, climbing and digging behaviors during 10 min of observation in a novel cage. (D) Social interaction test. Graph plots the duration of sniffing and following behaviors. (E) Novel object recognition test. Discrimination index was calculated as the ratio of time spent exploring the novel object vs the time spent exploring the familiar object in the choice trial. (F) Novel location recognition test. Discrimination index was calculated as the ratio of time spent exploring the displaced object vs the non-displaced object in the choice trial. (G) Motor coordination evaluation. Graphs plot the latency (s) that mice stayed on the rotating rod over 3 consecutive testing days. Data are represented as the mean + SEM. *P < 0.05, **P < 0.01 and ***P < 0.001, significantly different from their wt counterparts, Student’s t-test. (TIF) [file pgen.1005709.s002.tif]

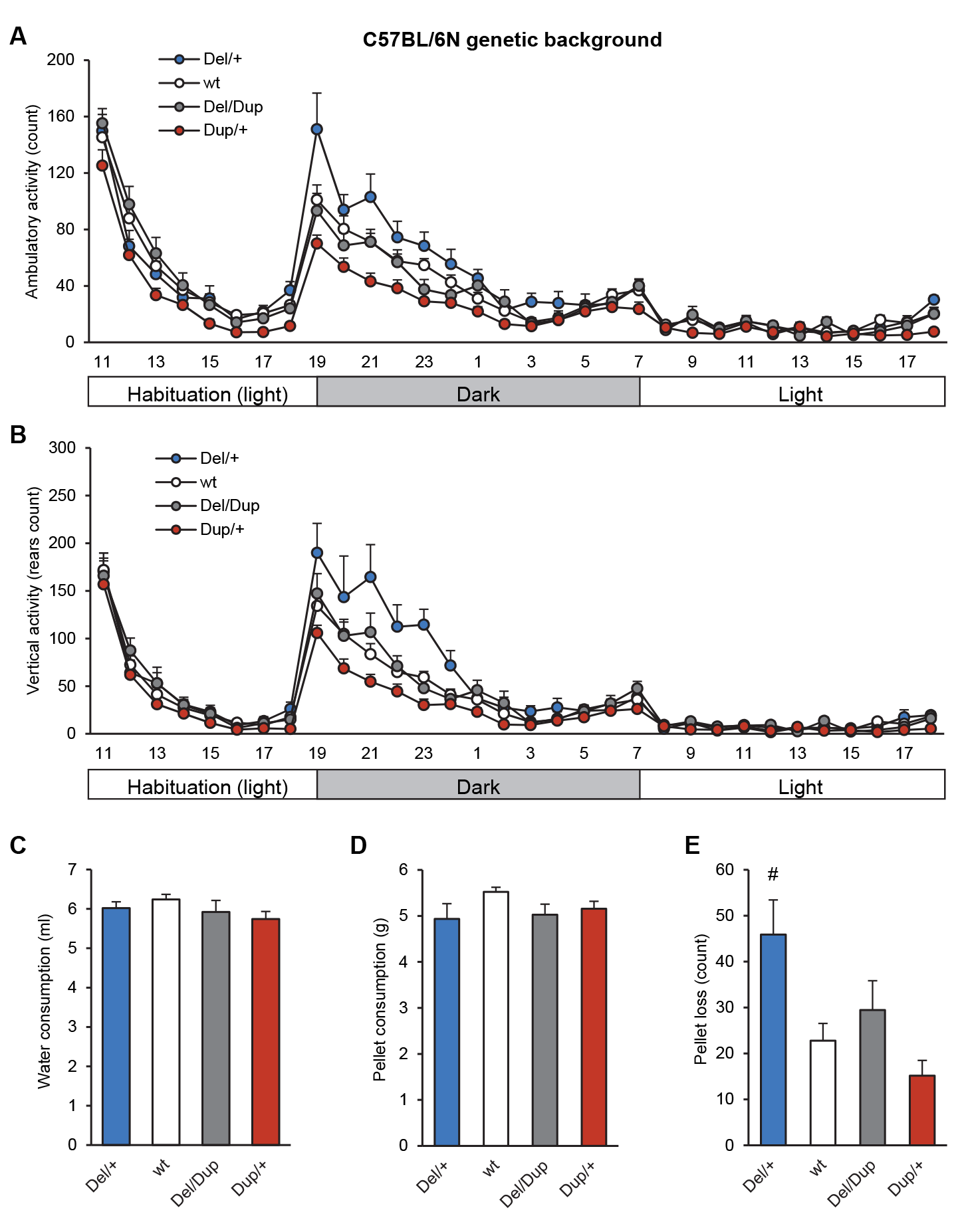

Supplement: S3 Fig — Patterns of locomotor activity (A) and vertical activity (B) profiles during 32-hours of test. Dup/+ mice showed a global hypoactivity for ambulatory and vertical activity while Del/+ mice are more active. (C-D) Feeding behaviors. Water (C) and pellet (D) consumption during the 32-hours of testing. (E) Pellets lost by animals which passed through the holed ground. Del/+ mice lost significantly more pellets compared to the other genotypes. Data are represented as the mean + SEM. # P < 0.05 vs all other groups. Mann Whitney U tests following a significant Kruskal Wallis analysis. (TIF) [file pgen.1005709.s003.tif]

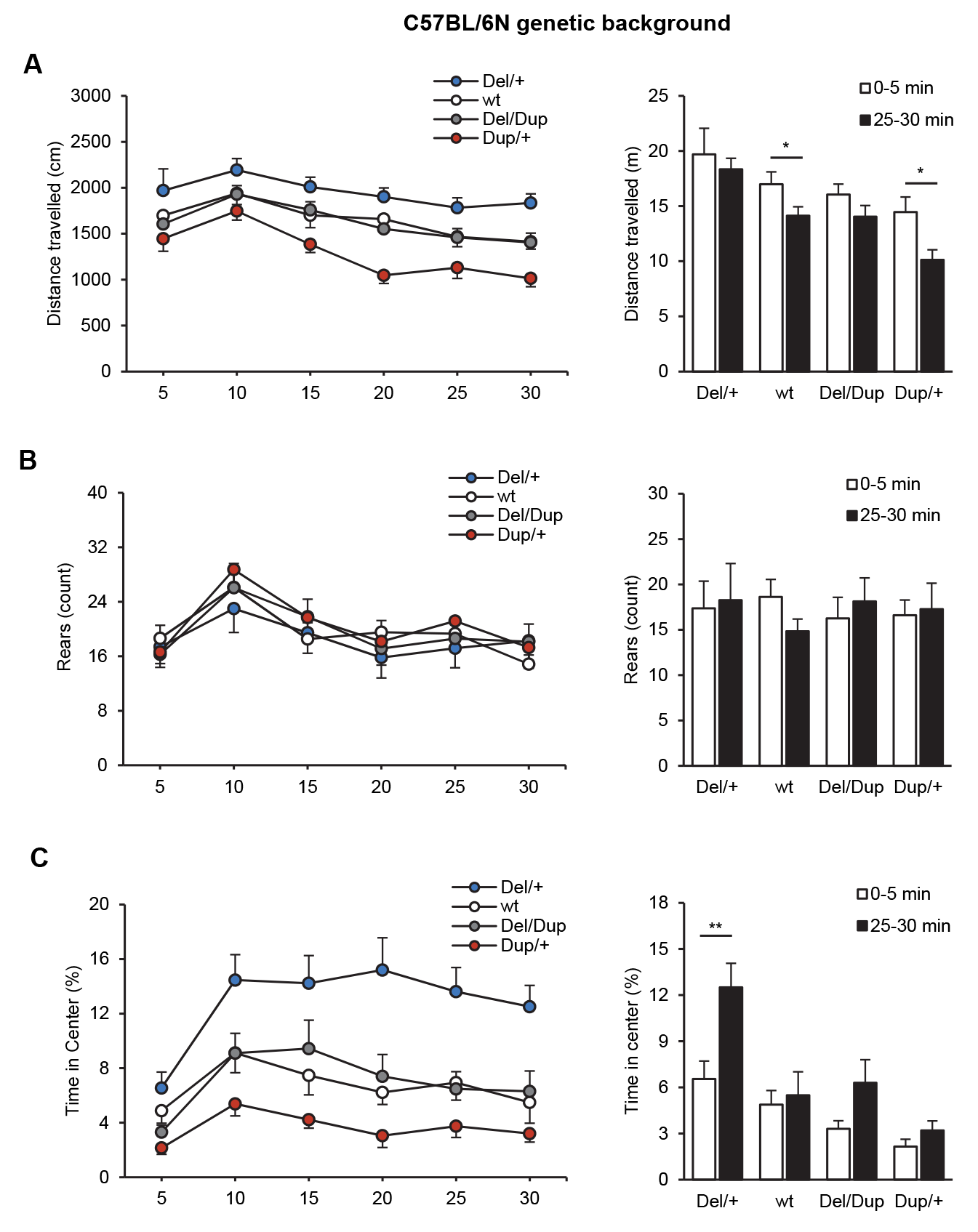

Supplement: S4 Fig — (A) Distance travelled (m). Whereas wt and Dup/+ mice showed a habituation and decreased their activity at the end of the test, Del/+ and Del/Dup mice showed a constant activity. (B) Vertical activity (rears). All genotypes showed similar patterns of vertical activity without habituation. (C) Time in center (%). Del/+ mice showed a marked increase of time spent in the arena center. Data are represented as the mean ± SEM. Student’s t-test, *P < 0.05, **P < 0.01. (TIF) [file pgen.1005709.s004.tif]

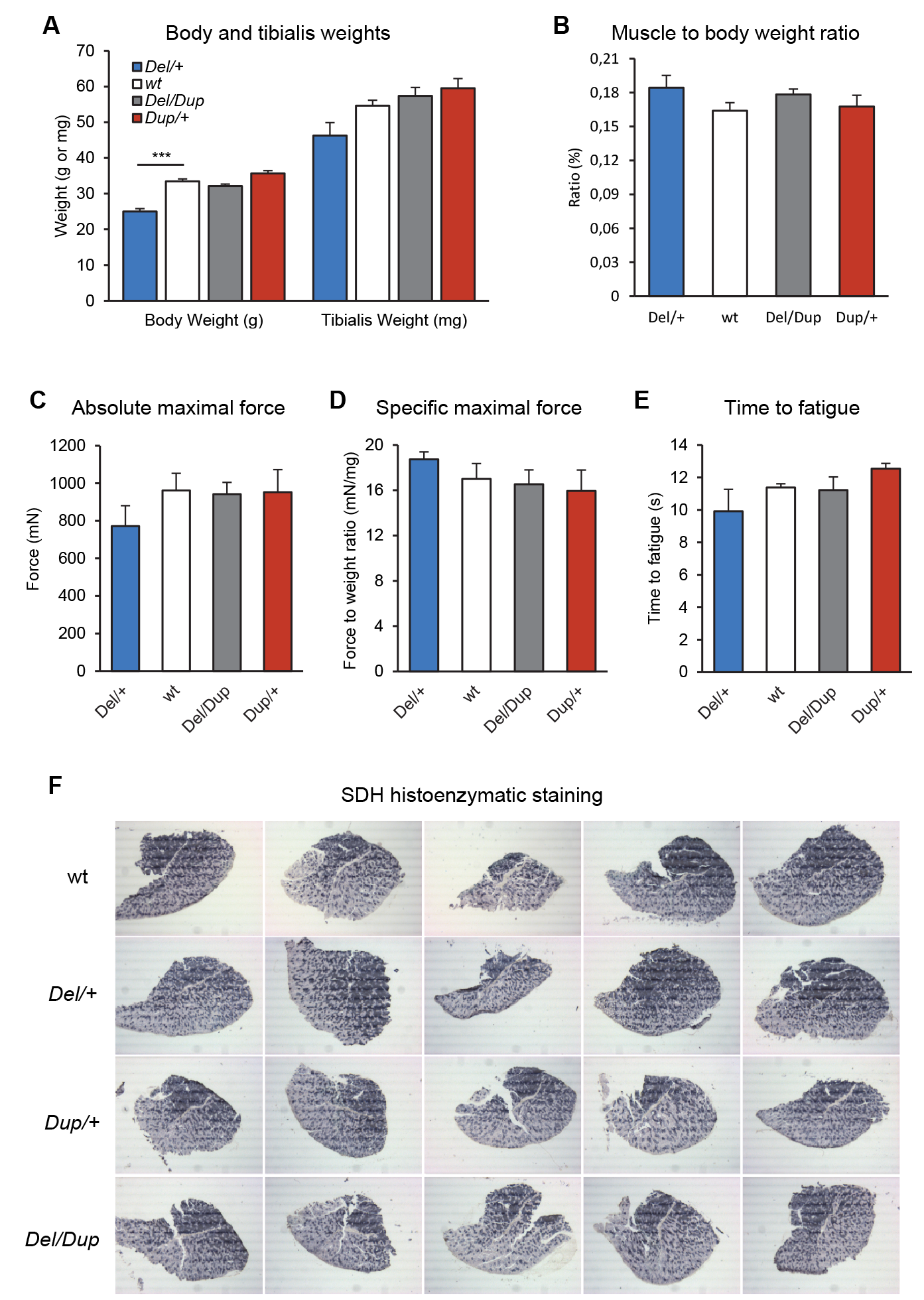

Supplement: S5 Fig — (A) Whole body weight (g) and TA weight (mg). (B) TA to body weight ratio. (C) Absolute maximal force of the TA muscle. (D) Specific maximal force of the TA. (E) Fatigue in TA muscle, measured as the time taken to reach 50% of maximum muscle force. (F) SDH histoenzymatic staining. Transverse cryosections (8 μm) of mouse skeletal muscles were prepared, fixed, and stained with succinate dehydrogenase (SDH). Data are represented as the mean + SEM. Tukey's test was applied following a significant one-way ANOVA, *** P < 0.001. (TIF) [file pgen.1005709.s005.tif]

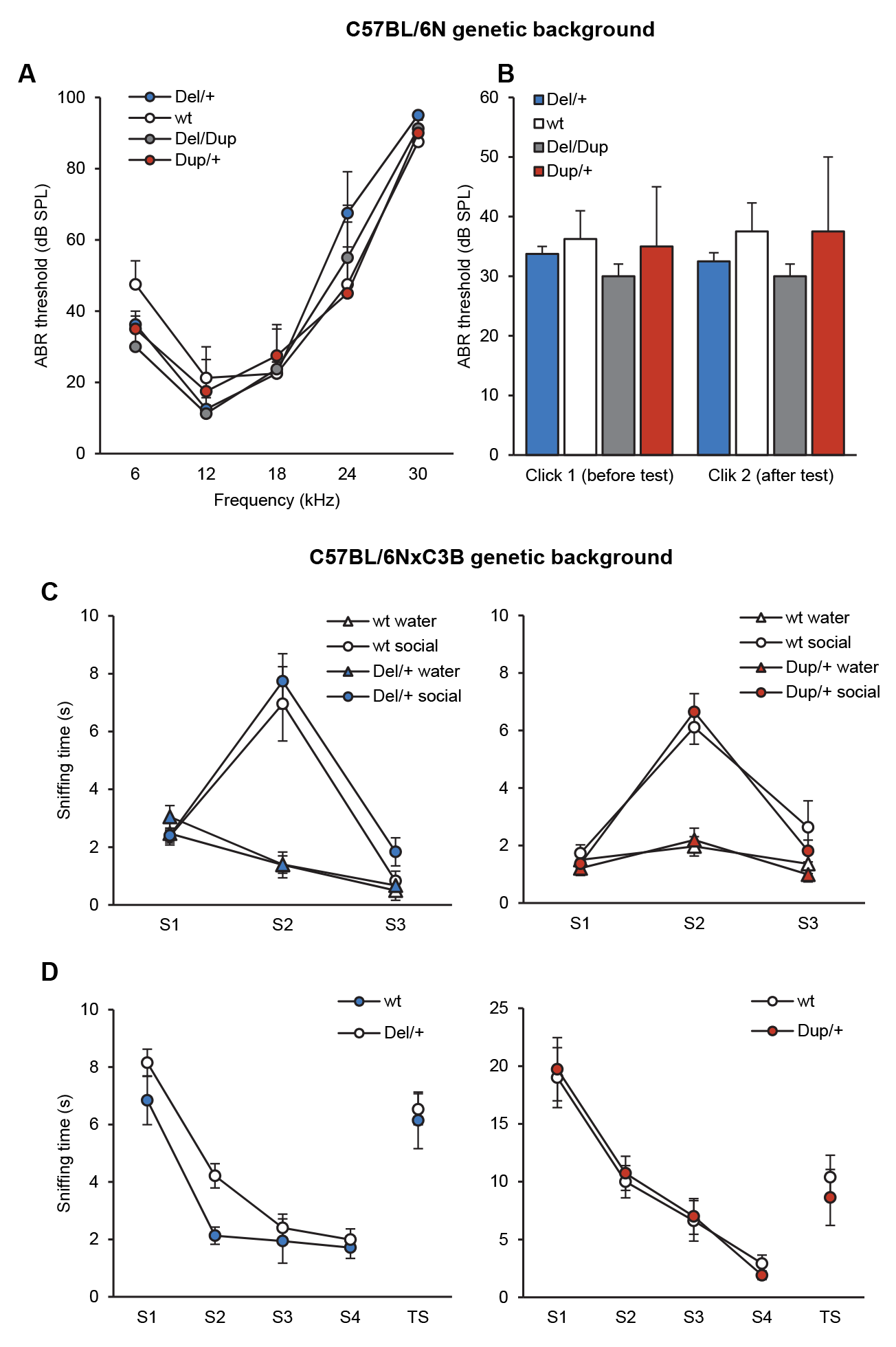

Supplement: S6 Fig — (A-B) Hearing capacities of the C57BL/6N Del-Dup cohort in the auditory brain response test. No differences were observed between mutant and control littermates for both genotypes. (C-D) Olfaction capacities of the separated C57BL/6NxC3B Del/+ and Dup/+ cohorts in the social (A) and non-social (B) odor discrimination tests. Data are represented as the mean ± SEM. (TIF) [file pgen.1005709.s006.tif]

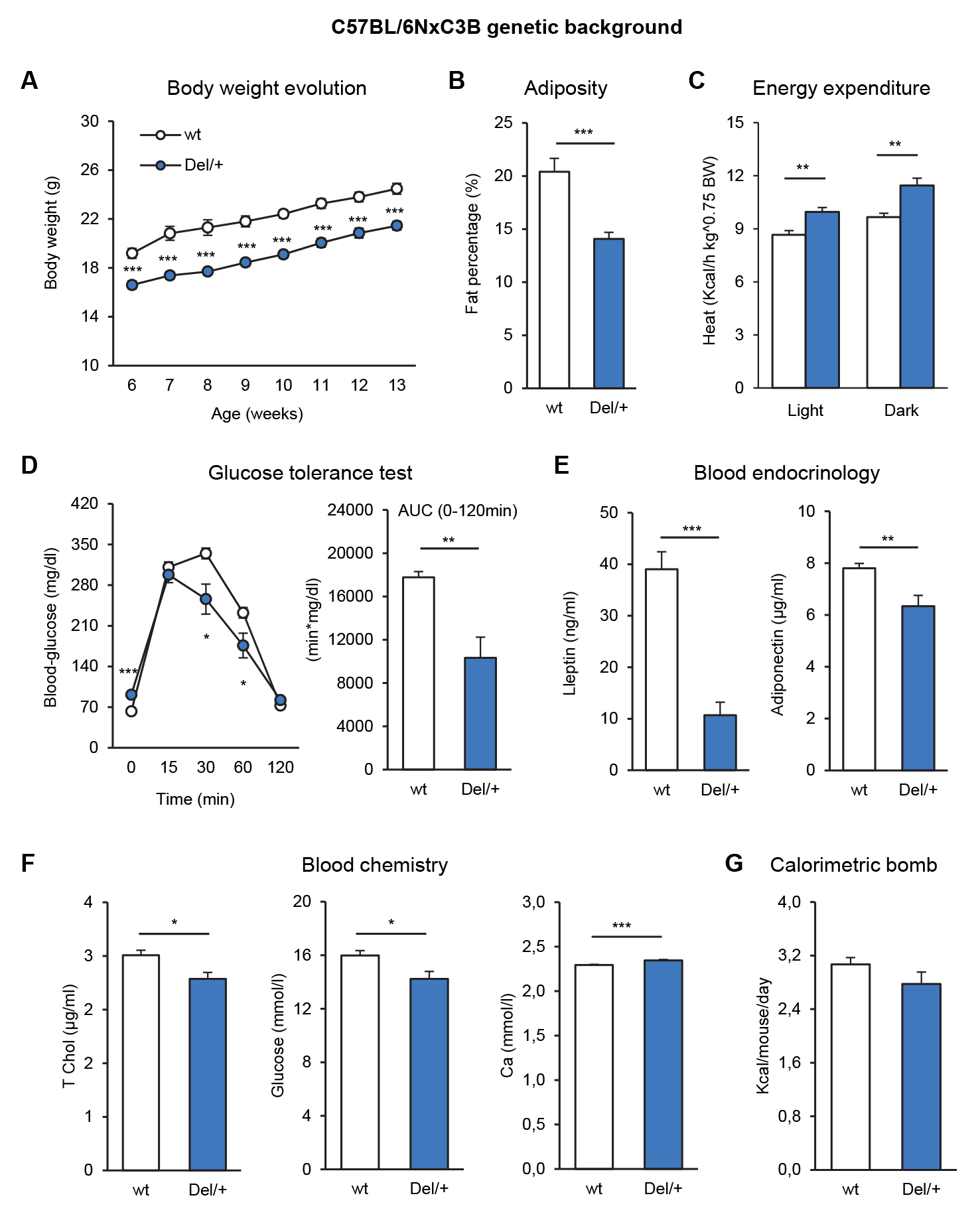

Supplement: S7 Fig — (A) Evolution of body weight (g). (B) Body fat percentage measured by qNMR. (C) Energy expenditure (Kcal/h per kg^0.75 of body weight) during 12-hour dark and 12-hour light phases. (D) Intraperitoneal glucose tolerance test. Evolution of blood glucose (mg/dl) and glucose area under the curve (AUC) (min*mg/dl) (E) Endocrinology analysis. Leptin (ng/ml) and adiponectin (μg/ml) blood levels. (F) Blood chemistry analysis. Total cholesterol (μg/ml), glucose (mmol/l) and calcium (mmol/l) blood levels. (G) Bomb Calorimetry. Total daily energy excreted (Kcal). Data are represented as the mean + SEM. Student’s t-test, *P < 0.05, **P < 0.01, ***P < 0.001. (TIF) [file pgen.1005709.s007.tif]

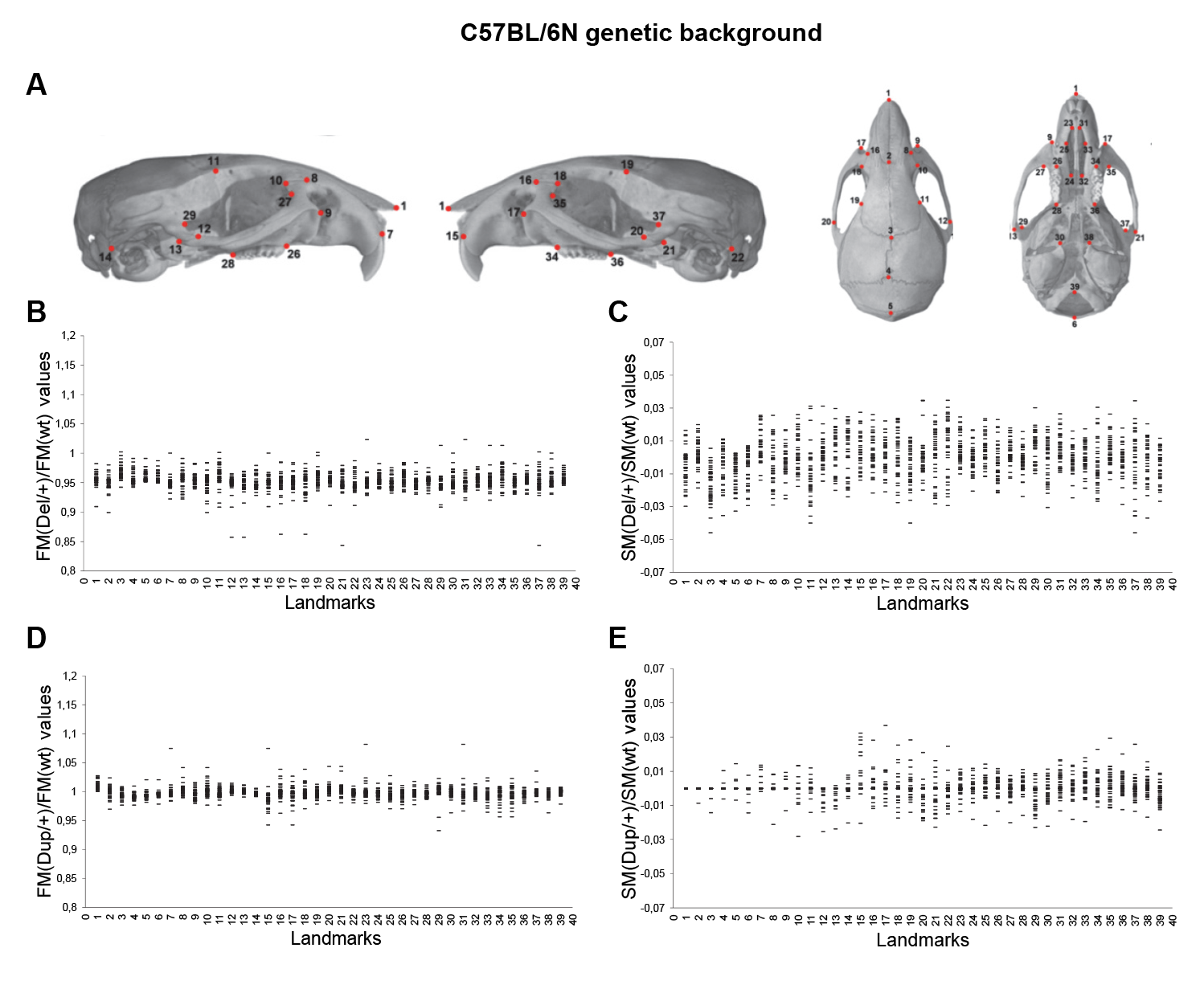

Supplement: S8 Fig — (A) Representative reconstructed 3D skull images and landmark distribution. Euclidian distances between the different landmarks allowed calculation of both the form (or size) difference (FD) and the shape difference (SD). Analysis revealed size reduction of the skull of Del/+ animals (B) and no alteration of skull size of Dup/+ animals (D). An alteration of skull shape was found for Del/+ (C) and Dup/+ (E) mice. (TIF) [file pgen.1005709.s008.tif]
