# Supplementary material for: Reciprocal Effects on Neurocognitive and Metabolic Phenotypes in Mouse Models of 16p11.2 Deletion and Duplication Syndromes
Source: PLoS Genet. 2016 Feb 12;12(2):e1005709. doi: 10.1371/journal.pgen.1005709 (PMC4752317; doi:10.1371/journal.pgen.1005709)
Supplement: S1 Table — Spontaneous circadian activity and feeding behaviors were assessed in the actimeter cages. Del/+ mice showed hyperactivity whereas Dup/+ mice showed hypoactivity during habituation (hab), dark and light phases. A considerable increase in the number of rears was observed for Del/+ mice during the dark phase. No feeding behavior phenotypes were noted during this test. Exploratory activity and emotional reactivity to novelty was assessed in the open field test. In comparison with wild-type littermates, Del/+ and Dup/+ mice travelled longer and shorter distances during the 30 min of test, respectively. Vertical activity/rears were also increased for Del/+ mice. Time spent in the center area, the most aversive part of the arena, was similar between mutants and controls. The elevated plus maze did not reveal any phenotypes. Observation of repetitive behaviors in a novel home cage during 10 min revealed a higher number of climbing, rearing, and jumping events in Del/+ mice and a lower number of digging, climbing, and rearing events in Dup/+ mice as compared to their wt counterparts. In comparison with controls, Del/+ mice buried less marbles in the marble burying test. Overall, Del/+ and Dup/+ mice showed hyperactivity and hypoactivity phenotypes, respectively. No signs of anxiety were detected in mutant mice. Data are shown as the mean ± SEM. *P < 0.05, **P < 0.01 and ***P < 0.001, significantly different from their wt counterparts, Student’s t-test. (DOCX) [file pgen.1005709.s009.docx]

**Supplementary Table S1.** Behavioral characterization of the separate *Del/+* and *Dup/+* cohorts on the C57BL/6N genetic background.

|  |  |  | |  |  | |  |
| --- | --- | --- | --- | --- | --- | --- | --- |
| Test | Parameter | B6N *Del/+* cohort results | | | B6N *Dup/+* cohort results | | |
|  |  | wt | Del/+ | | wt | Dup/+ | |
| Circadian Activity | Hab horizontal activity (count) | 301 ± 31 | 407 ± 46 | | 412 ± 24 | 272 ± 32 ** | |
|  | Hab vertical activity (count) | 305 ± 50 | 718 ± 137 ** | | 510 ± 50 | 304 ± 145 ** | |
|  | Dark horizontal activity (count) | 481 ± 22 | 694 ± 76 * | | 741 ± 103 | 415 ± 41 ** | |
|  | Dark vertical activity (count) | 568 ± 59 | 4692 ± 1168 ** | | 772 ± 94 | 489 ± 69 * | |
|  | Light horizontal activity (count) | 108 ± 9 | 136 ± 18 | | 226 ± 32 | 124 ± 14 ** | |
|  | Light vertical activity (count) | 87 ± 9 | 312 ± 106 * | | 139 ± 13 | 101 ± 14 | |
|  | Total food consumption (g) | 5.9 ± 0.1 | 5.8 ± 0.3 | | 5.4 ± 0.2 | 5.4 ± 0.4 | |
|  | Total water consumption (ml) | 5.3 ± 0.2 | 5.6 ± 0.2 | | 6.1 ± 0.3 | 5.8 ± 0.3 | |
| Open Field | Distance travelled (m) | 107 ± 3 | 123 ± 5 ** | | 125 ± 5 | 106 ± 5 * | |
|  | Rears (count) | 164 ± 10 | 229 ± 21 ** | | 214 ± 14 | 204 ± 22 | |
|  | Time in center (%) | 13.4 ± 1.3 | 13.2 ± 0.8 | | 19.9 ± 2.5 | 16.6 ± 2.1 | |
| Elevated Plus Maze | Arm entries (count) | 17.1 ± 1.1 | 17.3 ± 1.2 | | 15.6 ± 1.1 | 15.1 ± 1.6 | |
|  | Open arm time (%) | 10.1 ± 1.6 | 13.3 ± 3.0 | | 9.4 ± 1.7 | 7.6 ± 2.2 | |
| Stereotypy Observation | Digging (count) | 20.1 ± 2.6 | 43.6 ± 6.5 ** | | 29.0 ± 4.1 | 11.1 ± 1.9 *** | |
|  | Climbing (count) | 4.1 ± 0.7 | 12.9 ± 2.6 ** | | 5.0 ± 0.7 | 2.3 ± 0.6 * | |
|  | Rearing (count) | 39.0 ± 3.8 | 46.7 ± 3.3 | | 52.1 ± 3.2 | 36.5 ± 3.0 ** | |
|  | Jumping (count) | 0 | 42.0 ± 18.0 * | | 0 | 0 | |
| Marble burying | Uncovered marbles (%) | 31.0 ± 3.9 | 44.2 ± 4.2 *** | | 29.2 ± 7.3 | 39.0 ± 8.6 | |
|  | Fully covered marbles (%) | 51.9 ± 4.0 | 31.3 ± 4.5 | | 46.4 ± 6.8 | 32.2 ± 8.2 | |

Spontaneous circadian activity and feeding behaviors were assessed in the actimeter cages. *Del/+* mice showed hyperactivity whereas *Dup/+* mice showed hypoactivity during habituation (hab), dark and light phases. A considerable increase in the number of rears was observed for *Del/+* mice during the dark phase. No feeding behavior phenotypes were noted during this test. Exploratory activity and emotional reactivity to novelty was assessed in the open field test. In comparison with wild-type littermates, *Del/+* and *Dup/+* mice travelled longer and shorter distances during the 30 min of test, respectively. Vertical activity/rears were also increased for *Del/+* mice. Time spent in the center area, the most aversive part of the arena, was similar between mutants and controls. The elevated plus maze did not reveal any phenotypes. Observation of repetitive behaviors in a novel home cage during 10 min revealed a higher number of climbing, rearing, and jumping events in *Del/+* mice and a lower number of digging, climbing, and rearing events in *Dup/+* mice as compared to their wt counterparts. In comparison with controls, *Del/+* mice buried less marbles in the marble burying test. Overall, *Del/+* and *Dup/+* mice showed hyperactivity and hypoactivity phenotypes, respectively. No signs of anxiety were detected in mutant mice. Data are shown as the mean ± SEM. ^*^*P* < 0.05, ^**^*P* < 0.01 and ^***^*P* < 0.001, significantly different from their wt counterparts, Student’s t-test.
