# Supplementary material for: Reciprocal Effects on Neurocognitive and Metabolic Phenotypes in Mouse Models of 16p11.2 Deletion and Duplication Syndromes
Source: PLoS Genet. 2016 Feb 12;12(2):e1005709. doi: 10.1371/journal.pgen.1005709 (PMC4752317; doi:10.1371/journal.pgen.1005709)
Supplement: S2 Table — Working memory was first assessed with the Y maze test, which did not reveal any phenotypes in mutant mice. Memory abilities were then evaluated with novel object and novel location recognition tasks. In the acquisition session (S1), no differences in object exploration were noted between genotypes. After a 3-hour delay, Del/+ mice showed a recognition memory deficit for object location whereas Dup/+ mice showed memory improvements for object identity and location. In the Morris water maze, no change of spatial learning and memory was observed. Mutant and control mice travelled the same distance and needed the same duration to find the hidden platform from the first day to the sixth and last training day (D6). In the probe test (PT), performed on the 7th day, without the hidden platform, all mice spent the same percentage of time in the target quadrant. No phenotype was noted in the social interaction and three-chamber sociability tests. In the forced swim test, Del/+ animals floated less than 2n mice, which may be due to the hyperactivity phenotype of these mice. Prepulse inhibition (PPI) tests did not reveal any abnormalities in acoustic startle reflex responses and sensorimotor gating capacities and the pentylenetetrazole (PTZ) sensitivity test did not reveal any difference in seizure susceptibility between genotypes. In the rotarod test, Dup/+ mice did not show motor learning improvement with repeated testing (from the first day (D1) to the third day (D3)). Finally, the grip test indicated that Del/+ and Dup/+ mice showed stronger and weaker grip strength, respectively. Data are represented as the mean ± SEM. *P < 0.05, **P < 0.01 and ***P < 0.001, significantly different from wt counterparts, Student’s t-test. (DOCX) [file pgen.1005709.s010.docx]

**Supplementary Table S2. C**haracterization of cognitive and social behavior of *Del/+* and *Dup/+* cohorts on the C57BL/6N genetic background.

|  |  |  |  |  |  |
| --- | --- | --- | --- | --- | --- |
| Test | Parameter | B6N *Del/+* cohort results | | B6N *Dup/+* cohort results | |
|  |  | wt | Del/+ | wt | Dup/+ |
| Y Maze | Arm entries (count) | 16.2 ± 1.8 | 18.7 ± 1.5 | 21.2 ± 2.2 | 18.8 ± 2.7 |
|  | Alternation (%) | 68.0 ± 3.5 | 65.6 ± 3.7 | 58.3 ± 4.1 | 64.3 ± 4.0 |
| Novel Object Recognition 3 hour delay | S1 First object exploration (s) | 6.9 ± 0.6 | 7.2 ± 0.4 | 7.3 ± 0.8 | 7.1 ± 0.5 |
|  | S2 Former object exploration (s) | 2.8 ± 0.4 | 4.9 ± 0.9 * | 2.2 ± 0.4 | 1.8 ± 0.5 |
|  | S2 Novel object exploration (s) | 4.4 ± 0.5 | 6.6 ± 1.3 | 3.6 ± 0.6 | 4.1 ± 0.7 |
|  | Discrimination index (%) | 61.5 ± 1.9 | 57.4 ± 1.9 | 63.0 ± 2.6 | 73.5 ± 4.4 * |
| Novel Location Recognition 3 hour delay | S1 Object A exploration (s) | 3.9 ± 0.6 | 3.9 ± 0.5 | 5.8 ± 0.5 | 5.2 ± 0.6 |
|  | S1 Object B exploration (s) | 4.2 ± 0.8 | 3.9 ± 0.5 | 6.5 ± 0.7 | 5.4 ± 0.7 |
|  | S2 Fixed object explo. (s) | 2.2 ± 0.5 | 3.7 ± 0.9 | 3.8 ± 0.5 | 2.0 ± 0.3 ** |
|  | S2 Moved object explo. (s) | 3.1 ± 0.5 | 3.5 ± 0.4 | 5.0 ± 0.7 | 3.7 ± 0.6 |
|  | Discrimination index (%) | 60.1 ± 2.2 | 51.3 ± 2.6 * | 56.5 ± 1.9 | 65.2 ± 2.7 * |
| Morris watermaze | D6 distance to platform (m) | 6.7 ± 1.1 | 6.3 ± 0.9 | 6.3 ± 1.1 | 8.8 ± 2.1 |
|  | D6 latency to platform (s) | 49.7 ± 8.1 | 42.0 ± 8.0 | 42.6 ± 10.2 | 43.6 ± 9.4 |
|  | Probe - time in target quad. (%) | 44.8 ± 8.4 | 42.2 ± 9.6 | 41.3 ± 7.9 | 42.0 ± 5.3 |
| Social Interaction | Sniffing time (s) | 21.1 ± 3.4 | 18.4 ± 3.7 | 50.3 ± 8.9 | 67.4 ± 6.5 |
|  | Following time (s) | 3.4 ± 1.6 | 7.6 ± 3.9 | 11.5 ± 7.3 | 7.3 ± 3.7 |
| Three-Chamber Sociability | S1 First animal exploration (s) | 139 ± 13 | 129 ± 9 | 124 ± 13 | 113 ± 11 |
|  | S2 Former animal exploration (s) | 76.0 ± 7.8 | 81.9 ± 8.7 | 43.1 ± 4.1 | 35.8 ± 3.0 |
|  | S2 Novel animal exploration (s) | 110 ± 11 | 112 ± 11 | 75.4 ± 11.2 | 62.2 ± 7.8 |
|  | Discrimination index (%) | 58.5 ± 3.6 | 58.1 ± 4.2 | 61.9 ± 2.3 | 62.2 ± 2.8 |
| Forced Swimming | Floating time (%) | 74.5 ± 1.2 | 57.6 ± 5.7 ** | 75.9 ± 1.0 | 75.5 ± 1.9 |
| Prepulse Inhibition (PPI) | 120dB/40ms startle reflex | 113 ± 15 | 135 ± 11 | 136 ± 16 | 110 ± 20 |
|  | Global prepulse inhibition (%) | 68.0 ± 2.3 | 68.3 ± 1.7 | 74.9 ± 1.8 | 69.2 ± 4.1 |
| PTZ sensitivity | Clonic seizure latency (s) | 227 ± 33 | 255 ± 61 | 411 ± 77 | 368 ± 15 |
|  | Clonic seizure duration (s) | 48.6 ± 8.0 | 65.6 ± 15.5 | 30.7 ± 5.6 | 55.3 ± 13.2 |
| Rotarod | D1 Time on the rod (s) | 138 ± 16 | 153 ± 19 | 158 ± 23 | 144 ± 19 |
|  | D2 Time on the rod (s) | 202 ± 13 | 204 ± 24 | 194 ± 15 | 169 ± 16 |
|  | D3 Time on the rod (s) | 206 ± 14 | 202 ± 13 | 207 ± 15 | 148 ± 11 ** |
| Grip Test | Grip strength (g/g body weight) | 7.7 ± 0.2 | 9.7 ± 0.3 *** | 7.2 ± 0.4 | 5.8 ± 0.3 * |

Working memory was first assessed with the Y maze test, which did not reveal any phenotypes in mutant mice. Memory abilities were then evaluated with novel object and novel location recognition tasks. In the acquisition session (S1), no differences in object exploration were noted between genotypes. After a 3-hour delay, *Del/+* mice showed a recognition memory deficit for object location whereas *Dup/+* mice showed memory improvements for object identity and location. In the Morris water maze, no change of spatial learning and memory was observed. Mutant and control mice travelled the same distance and needed the same duration to find the hidden platform from the first day to the sixth and last training day (D6). In the probe test (PT), performed on the 7^th^ day, without the hidden platform, all mice spent the same percentage of time in the target quadrant. No phenotype was noted in the social interaction and three-chamber sociability tests. In the forced swim test, *Del/+* animals floated less than 2n mice, which may be due to the hyperactivity phenotype of these mice. Prepulse inhibition (PPI) tests did not reveal any abnormalities in acoustic startle reflex responses and sensorimotor gating capacities and the pentylenetetrazole (PTZ) sensitivity test did not reveal any difference in seizure susceptibility between genotypes. In the rotarod test, *Dup/+* mice did not show motor learning improvement with repeated testing (from the first day (D1) to the third day (D3)). Finally, the grip test indicated that *Del/+* and *Dup/+* mice showed stronger and weaker grip strength, respectively. Data are represented as the mean ± SEM. ^*^*P* < 0.05, ^**^*P* < 0.01 and ^***^*P* < 0.001, significantly different from wt counterparts, Student’s t-test.
