# Supplementary material for: Reciprocal Effects on Neurocognitive and Metabolic Phenotypes in Mouse Models of 16p11.2 Deletion and Duplication Syndromes
Source: PLoS Genet. 2016 Feb 12;12(2):e1005709. doi: 10.1371/journal.pgen.1005709 (PMC4752317; doi:10.1371/journal.pgen.1005709)
Supplement: S3 Table — In the circadian activity test, Del/+ mice showed vertical hyperactivity during dark phase whereas Dup/+ mice showed vertical hypoactivity during light and dark phases and ambulatory hypoactivity during light phase (statistical values are summarized in S3 Table). During open field tests, Dup/+ mice travelled a shorter distance and spent less time in arena center whereas Del/+ mice spent more time in the arena center. Observation of repetitive behaviors revealed an increase in the level of rearing and jumping for Del/+ mice. Data are represented as the mean ± SEM. (DOCX) [file pgen.1005709.s011.docx]

**Supplementary Table S3.**  Characterization of circadian activity, emotional reactivity and repetitive behaviors in the *Del-Dup* cohort on C57BL/6N genetic background.

|  |  |  |  | | |  | |  |
| --- | --- | --- | --- | --- | --- | --- | --- | --- |
| Test | Parameter | B6N *Del-Dup* cohort results | | | | | | |
|  |  | Del/+ | | wt | Del/Dup | | Dup/+ | |
| Circadian Activity | Hab horizontal activity (count) | 367 ± 44 | | 393± 36 | 414 ± 51 | | 275 ± 32 | |
|  | Hab vertical activity (count) | 722 ± 363 | | 353 ± 40 | 375 ± 46 | | 292 ± 61 | |
|  | Dark horizontal activity (count) | 725 ± 92 | | 552 ± 39 | 512 ± 61 | | 371 ± 40 | |
|  | Dark vertical activity (count) | 2178 ± 1225 | | 629 ± 57 | 672 ± 82 | | 432 ± 32 | |
|  | Light horizontal activity (count) | 176 ± 13 | | 175 ± 14 | 169 ± 13 | | 104 ± 15 | |
|  | Light vertical activity (count) | 128 ± 12 | | 135 ± 15 | 140 ± 19 | | 79.3 ± 10.0 | |
|  | Total food consumption (g) | 4.9 ± 0.3 | | 5.5 ± 0.1 | 5.0 ± 0.2 | | 5.2 ± 0.2 | |
|  | Total water consumption (ml) | 6.0 ± 0.2 | | 6.3 ± 0.1 | 5.9 ± 0.3 | | 5.7 ± 0.2 | |
| Open Field | Distance travelled (m) | 117 ± 6 | | 98.8 ± 5.5 | 97.1 ± 4.4 | | 79.3 ± 4.5 | |
|  | Rears (count) | 111 ± 15 | | 117 ± 9 | 118 ± 14 | | 123 ± 13 | |
|  | Time in centre (%) | 12.8 ± 1.1 | | 6.7 ± 1.0 | 7.0 ± 1.1 | | 4.0 ± 0.5 | |
| Stereotypy Observation | Digging (count) | 22.7 ± 4.9 | | 22.8 ± 3.1 | 17.9 ± 2.6 | | 17.9 ± 1.6 | |
|  | Climbing (count) | 23.1 ± 4.5 | | 12.9 ± 2.1 | 11.6 ± 2.3 | | 8.1 ± 0.9 | |
|  | Rearing (count) | 59.3 ± 5.1 | | 44.9 ± 2.2 | 45.3 ± 3.4 | | 42.1 ± 1.8 | |
|  | Jumping (count) | 23.3 ± 22.1 | | 0.2 ± 0.1 | 0 | | 0.3 ± 0.2 | |

In the circadian activity test, *Del/+* mice showed vertical hyperactivity during dark phase whereas *Dup/+* mice showed vertical hypoactivity during light and dark phases and ambulatory hypoactivity during light phase (statistical values are summarized in Supplementary Table S3). During open field tests, *Dup/+* mice travelled a shorter distance and spent less time in arena center whereas *Del/+* mice spent more time in the arena center. Observation of repetitive behaviors revealed an increase in the level of rearing and jumping for *Del/+* mice. Data are represented as the mean ± SEM.
