# Supplementary material for: Reciprocal Effects on Neurocognitive and Metabolic Phenotypes in Mouse Models of 16p11.2 Deletion and Duplication Syndromes
Source: PLoS Genet. 2016 Feb 12;12(2):e1005709. doi: 10.1371/journal.pgen.1005709 (PMC4752317; doi:10.1371/journal.pgen.1005709)
Supplement: S4 Table — Object recognition memory of mice was assessed with two retention delays, 30 min and 3-hours. Del/+ mice showed short-term memory deficits at both delays (statistical values are summarized in S2 Table). Dup/+ and Del/Dup mice showed improvement at the 3-hour delay. No anhedonia phenotype was observed in the sucrose preference test. No phenotype was detected in the social interaction test. During the rotarod test, Del/+ mice showed trends for improvement whereas Dup/+ and Del/Dup mice showed impairment trends (not significant). Del/+ animals also showed impairments in the notched bar test. Del/+ and Dup/+ mice showed stronger and weaker grip strength in the grip test, respectively. Data are represented as the mean ± SEM. (DOCX) [file pgen.1005709.s012.docx]

**Supplementary Table S4.** Behavioral characterization of the *Del-Dup* cohort on the C57BL/6N genetic background.

|  |  |  |  | |  |  |
| --- | --- | --- | --- | --- | --- | --- |
| Test | Parameter | B6N *Del-Dup* cohort results | | | | |
|  |  | Del/+ | | wt | Del/Dup | Dup/+ |
| New Object Recognition 30 min delay | S1 First object exploration (s) | 6.7 ± 0.7 | | 6.8 ± 0.7 | 6.6 ± 0.8 | 6.5 ± 0.4 |
|  | S2 Former object exploration (s) | 5.4 ± 0.7 | | 4.4 ± 0.6 | 4.7 ± 0.6 | 3.7 ± 0.6 |
|  | S2 Novel object exploration (s) | 6.3 ± 0.8 | | 8.0 ± 0.8 | 8.3 ± 1.0 | 7.7 ± 1.1 |
|  | Discrimination index (%) | 54.4 ± 1.7 | | 65.1 ± 2.1 | 63.7 ± 2.7 | 69.3 ± 1.7 |
| New Object Recognition 3 hour delay | S1 First object exploration (s) | 10.7 ± 1.1 | | 12.1 ± 0.6 | 12.3 ± 1.3 | 10.2 ± 1.0 |
|  | S2 Former object exploration (s) | 6.8 ± 1.4 | | 6.3 ± 1.0 | 4.6 ± 0.6 | 3.6 ± 0.4 |
|  | S2 Novel object exploration (s) | 8.4 ± 1.9 | | 9.2 ± 1.1 | 8.6 ± 0.8 | 8.0 ± 1.3 |
|  | Discrimination index (%) | 55.0 ± 1.8 | | 60.4 ± 1.6 | 65.8 ± 2.0 | 66.2 ± 2.2 |
| Sucrose Preference | D1 sucrose preference (%) | 65.7 ± 4.4 | | 70.8 ± 4.1 | 71.3 ± 3.6 | 74.6 ± 3.6 |
|  | D2 sucrose preference (%) | 68.7 ± 5.6 | | 76.6 ± 4.0 | 71.7 ± 2.7 | 71.5 ± 4.7 |
|  | D3 sucrose preference (%) | 81.5 ± 2.6 | | 78.2 ± 4.1 | 76.1 ± 2.1 | 71.7 ± 2.7 |
| Social Interaction | Sniffing time (s) | 73.8 ± 7.9 | | 60.4 ± 4.4 | 61.0 ± 3.6 | 73.9 ± 7.5 |
|  | Following time (s) | 18.2 ± 3.9 | | 11.4 ± 2.8 | 18.1 ± 5.3 | 16.0 ± 4.7 |
| Rotarod | D1 Time on the rod (s) | 173 ± 18 | | 157 ± 13 | 132 ± 11 | 138 ± 14 |
|  | D2 Time on the rod (s) | 188 ± 12 | | 170 ± 8 | 157 ± 13 | 147 ± 12 |
|  | D3 Time on the rod (s) | 203 ± 15 | | 180 ± 11 | 149 ± 12 | 144 ± 14 |
| Notched Bar | Hind limb errors (%) | 5.5 ± 0.8 | | 2.9 ± 0.4 | 3.6 ± 0.6 | 2.4 ± 0.4 |
| Grip Test | Grip strength (g/g body weight) | 10.8 ± 0.2 | | 9.3 ± 0.2 | 9.2 ± 0.4 | 7.6 ± 0.2 |

Object recognition memory of mice was assessed with two retention delays, 30 min and 3-hours. *Del/+* mice showed short-term memory deficits at both delays (statistical values are summarized in Table S2). *Dup/+* and *Del/Dup* mice showed improvement at the 3-hour delay. No anhedonia phenotype was observed in the sucrose preference test. No phenotype was detected in the social interaction test. During the rotarod test, *Del/+* mice showed trends for improvement whereas *Dup/+* and *Del/Dup* mice showed impairment trends (not significant). *Del/+* animals also showed impairments in the notched bar test. *Del/+* and *Dup/+* mice showed stronger and weaker grip strength in the grip test, respectively. Data are represented as the mean ± SEM.
