# Supplementary material for: Reciprocal Effects on Neurocognitive and Metabolic Phenotypes in Mouse Models of 16p11.2 Deletion and Duplication Syndromes
Source: PLoS Genet. 2016 Feb 12;12(2):e1005709. doi: 10.1371/journal.pgen.1005709 (PMC4752317; doi:10.1371/journal.pgen.1005709)
Supplement: S5 Table — The table summarizes post-hoc analysis of parameters for which analysis of variance revealed significant differences between genotypes. *p < 0.05, **p < 0.01 and ***p < 0.001, Tukey's test following a significant one-way ANOVA analysis. +p < 0.05, ++p < 0.01 and +++p < 0.001, Mann Whitney U tests following a significant Kruskal Wallis analysis. (DOCX) [file pgen.1005709.s013.docx]

**Supplementary Table S5.** Statistical values for the *Del-Dup* behavioral characterization on the C57BL/6N genetic background.

| Circadian activity test |  |  |  |  |
| --- | --- | --- | --- | --- |
|  | *Del/+* vs wt | *Dup/+* vs wt | Del/Dup vs wt | Del/+ vs Dup/+ |
| Dark horizontal activity | q=3.108, p=0.138 | q=3.609, p=0.064 | q=0.763, p=0.949 | q=6.002, p<0.001 *** |
| Dark vertical activity | U=43, p=0.039 + | U=66, p=0.015 + | U=105, p=0.730 | U=5, p<0.001 +++ |
| Light horizontal activity | q=0.122, p=0.997 | q=5.227, p=0.003 ** | q=0.424, p=0.991 | q=4.562, p=0.012 * |
| Light vertical activity | U=85, p=1.000 | U=59.5, p=0.008 ++ | U=109.5, p=0.871 | U=20.5, p=0.008 ++ |
| Open field test |  |  |  |  |
|  | Del/+ vs 2n | Dup/+ vs 2n | Del/Dup vs 2n | Del/+ vs Dup/+ |
| Distance travelled | q=3.388, p=0.089 | q=4.474, p=0.013 * | q=0.341, p=0.995 | q=7.178, p<0.001 *** |
| Time in center | U=29, p=0.001 ++ | U=92, p=0.029 + | U=140, p=0.945 | U=2.5, p=0.004 ++ |
| Stereotypy count |  |  |  |  |
|  | Del/+ vs 2n | Dup/+ vs 2n | Del/Dup vs 2n | Del/+ vs Dup/+ |
| Climbing | U=27, p=0.039 + | U=53, p=0.074 | U=47, p=0.642 | U=26.5, p=0.015 + |
| Rearing | q=4.682, p=0.010 * | q=1.029, p=0.886 | q=0.135, p=1.000 | q=5.842, p=0.001 ** |
| Jumping | U=24, p=0.016 + | U=88, p=0.906 | U=45, p=0.235 | U=30, p=0.008 ++ |
| NOR test (30 min delay) |  |  |  |  |
|  | Del/+ vs 2n | Dup/+ vs 2n | Del/Dup vs 2n | Del/+ vs Dup/+ |
| Discrimination index | q=5.054, p=0.004 ** | q=2.107, p=0.451 | q=0.711, p=0.958 | q=6.826, p<0.001 *** |
| NOR test (3 hour delay) |  |  |  |  |
|  | Del/+ vs 2n | Dup/+ vs 2n | Del/Dup vs 2n | Del/+ vs Dup/+ |
| Discrimination index | U=38, p=0.043 + | U=66, p=0.021 + | U=54, p=0.048 + | U=22, p=0.002 ++ |
| Rotarod test |  |  |  |  |
|  | Del/+ vs 2n | Dup/+ vs 2n | Del/Dup vs 2n | Del/+ vs Dup/+ |
| D1 Time on the rod | q=1.128, p=0.855 | q=1.656, p=0.647 | q=2.088, p=0.456 | q=2.878, p=0.182 |
| D2 Time on the rod | q=1.275, p=0.804 | q=1.950, p=0.516 | q=1.101, p=0.864 | q=2.923, p=0.172 |
| D3 Time on the rod | q=1.631, p=0.658 | q=3.065, p=0.140 | q=2.563, p=0.274 | q=4.221, p=0.019 * |
| Notched bar test |  |  |  |  |
|  | Del/+ vs 2n | Dup/+ vs 2n | Del/Dup vs 2n | Del/+ vs Dup/+ |
| Hind limb errors (%) | U=41.5, p=0.007 ++ | U=144.5, p=0.298 | U=113, p=0.313 | U=29, p=0.001 ++ |
| Grip test |  |  |  |  |
|  | Del/+ vs 2n | Dup/+ vs 2n | Del/Dup vs 2n | Del/+ vs Dup/+ |
| Grip strength | q=5.447, p=0.002 ** | q=7.175, p<0.001 *** | q=0.321, p=0.996 | q=11.591, p<0.001 *** |

The table summarizes post-hoc analysis of parameters for which analysis of variance revealed significant differences between genotypes. ^*^*p* < 0.05, ^**^*p* < 0.01 and ^***^*p* < 0.001, Tukey's test following a significant one-way ANOVA analysis. ^+^*p* < 0.05, ^++^*p* < 0.01 and ^+++^*p* < 0.001, Mann Whitney U tests following a significant Kruskal Wallis analysis.
