# Supplementary material for: Reciprocal Effects on Neurocognitive and Metabolic Phenotypes in Mouse Models of 16p11.2 Deletion and Duplication Syndromes
Source: PLoS Genet. 2016 Feb 12;12(2):e1005709. doi: 10.1371/journal.pgen.1005709 (PMC4752317; doi:10.1371/journal.pgen.1005709)
Supplement: S6 Table — In the circadian activity test, Del/+ mice showed increased horizontal activity during the dark phase and enhanced vertical activity during the dark and the light phases whereas Dup/+ mice showed reduced vertical activity during the dark phase. No behavioral phenotypes were detected in in the open field test. The marble burying test, which was only done for Del/+ cohort, also did not reveal any phenotypes in mutant mice. Finally, observation of the animals in a novel home cage revealed increased and decreased level of climbing behavior in Del/+ and Dup/+ mice, respectively. NT: not tested. Data are shown as mean ± SEM. *p < 0.05, **P < 0.01 and ***P < 0.001, significantly different from their wt counterparts, Student’s t-test. (DOCX) [file pgen.1005709.s014.docx]

**Supplementary Table S6.** Characterization of circadian activity, emotional reactivity and repetitive behaviors in *Del/+* and *Dup/+* cohorts on the C57BL/6NxC3B genetic background.

| Test | Parameter | B6C3B *Del/+* cohort results | | B6C3B *Dup/+* cohort results | |
| --- | --- | --- | --- | --- | --- |
|  |  | wt | Del/+ | wt | Dup/+ |
| Circadian Activity | Hab horizontal activity (count) | 206 ± 20 | 220± 16 | 170 ± 13 | 172 ± 14 |
|  | Hab vertical activity (count) | 331 ± 57 | 393 ± 45 | 319 ± 28 | 300 ± 34 |
|  | Dark horizontal activity (count) | 316 ± 34 | 449 ± 38 * | 350 ± 21 | 310 ± 24 |
|  | Dark vertical activity (count) | 524 ± 49 | 814 ± 84 ** | 709 ± 49 | 518 ± 43 ** |
|  | Light horizontal activity (count) | 114 ± 8 | 129 ± 7 | 120 ± 9 | 131 ± 12 |
|  | Light vertical activity (count) | 118 ± 12 | 164 ± 11 ** | 147 ± 14 | 123 ± 11 |
|  | Total food consumption (g) | 5.1 ± 0.1 | 4.8 ± 0.1 | 5.2 ± 0.3 | 5.6 ± 0.3 |
|  | Total water consumption (ml) | 5.8 ± 0.2 | 6.1 ± 0.2 | 5.8 ± 0.2 | 6.1 ± 0.2 |
| Open Field | Distance travelled (m) | 93.4 ± 5.1 | 105 ± 5 | 83.2 ± 3.4 | 79.6 ± 5.0 |
|  | Rears (count) | 194 ± 17 | 213 ± 18 | 158 ± 12 | 163 ± 15 |
|  | Time in centre (%) | 10.6 ± 1.5 | 14.1 ± 1.7 | 10.4 ± 1.4 | 13.6 ± 2.0 |
| Stereotypy Observation | Digging (count) | 11.4 ± 2.3 | 15.9 ± 2.5 | 6.0 ± 0.9 | 5.7 ± 0.9 |
|  | Climbing (count) | 8.2 ± 1.2 | 16.2 ± 2.2 ** | 10.3 ± 1.5 | 6.2 ± 1.1 * |
|  | Rearing (count) | 40.5 ± 2.2 | 44.9 ± 2.5 | 39.3 ± 3.2 | 33.9 ± 2.9 |
| Marble burying | Uncovered marbles (%) | 35.1 ± 5.4 | 41.7 ± 4.4 | NT | |
|  | Partially covered marbles (%) | 16.7 ± 2.1 | 19.6 ± 1.7 |  |  |
|  | Fully covered marbles (%) | 48.3 ± 5.5 | 38.7 ± 4.3 |  |  |

In the circadian activity test, *Del/+* mice showed increased horizontal activity during the dark phase and enhanced vertical activity during the dark and the light phases whereas *Dup/+* mice showed reduced vertical activity during the dark phase. No behavioral phenotypes were detected in in the open field test. The marble burying test, which was only done for *Del/+* cohort, also did not reveal any phenotypes in mutant mice. Finally, observation of the animals in a novel home cage revealed increased and decreased level of climbing behavior in *Del/+* and *Dup/+* mice, respectively. NT: not tested. Data are shown as mean ± SEM. ^*^*p* < 0.05, ^**^*P* < 0.01 and ^***^*P* < 0.001, significantly different from their wt counterparts, Student’s t-test.
