# Supplementary material for: Reciprocal Effects on Neurocognitive and Metabolic Phenotypes in Mouse Models of 16p11.2 Deletion and Duplication Syndromes
Source: PLoS Genet. 2016 Feb 12;12(2):e1005709. doi: 10.1371/journal.pgen.1005709 (PMC4752317; doi:10.1371/journal.pgen.1005709)
Supplement: S7 Table — Working memory was first assessed with the Y maze test, which revealed an improvement in Dup/+ animals. Recognition memory was first evaluated with the novel object recognition task. In the acquisition session (S1), no difference in object exploration was noticed between genotypes. After a 3-hour delay, Del/+ and Dup/+ mice show memory impairment and improvement for object identity, respectively. We did the same test for the Dup/+ cohort with a 24-hour delay and found no phenotype, suggesting that duplication of Sult1a1–Spn impacts mnemonic processes associated with short-term memory. With the Del/+ cohort, we performed the novel place recognition task with a 3-hour delay and also found a memory deficit for object location. In the social interaction test, we found a decrease of sniffing behavior for Del/+ mice and a decrease of sniffing and following behaviors for Dup/+ animals. In the three-chamber sociability test, we did not observe differences in social interaction, but novel animal discrimination was significantly decreased for Del/+ animals. No motor coordination phenotype was seen in rotarod and notched bar tests but Del/+ and Dup/+ mice still presented stronger and weaker grip strength in the grip test, respectively. NT: not tested. Data are shown as the mean ± SEM. *P < 0.05, **P < 0.01 and ***P < 0.001, significantly different from wt counterparts, Student’s t-test. (DOCX) [file pgen.1005709.s015.docx]

**Supplementary Table S7.** Behavioral characterization of *Del/+* and *Dup/+* cohorts on the C57BL/6NxC3B genetic background.

| Test | Parameter | B6C3B *Del/+* cohort results | | B6C3B *Dup/+* cohort results | |
| --- | --- | --- | --- | --- | --- |
|  |  | wt | Del/+ | wt | Dup/+ |
| Y Maze | Arm entries (count) | 26.4 ± 1.5 | 30.1 ± 1.9 | 29.7 ± 1.4 | 23.4 ± 1.1 ** |
|  | Alternation (%) | 65.9 ± 2.2 | 66.7 ± 2.6 | 65.3 ± 2.4 | 73.1 ± 2.2 * |
| Novel Object Recognition 3 hour delay | S1 First object exploration (s) | 10.0 ± 1.1 | 10.5 ± 1.0 | 7.1 ± 0.9 | 5.1 ± 0.7 |
|  | S2 Former object exploration (s) | 4.1 ± 1.1 | 4.6 ± 0.5 | 4.2 ± 0.5 | 1.8 ± 0.6 ** |
|  | S2 Novel object exploration (s) | 9.1 ± 2.0 | 5.8 ± 0.7 | 7.3 ± 0.6 | 6.2 ± 0.6 |
|  | Discrimination index (%) | 69.3 ± 2.6 | 55.8 ± 1.6 *** | 64.4 ± 3.0 | 78.4 ± 1.9 ** |
| Novel Object Recognition 24 hour delay | S1 First object exploration (s) | NT | | 14.0 ± 2.0 | 13.5 ± 2.5 |
|  | S2 Former object exploration (s) |  |  | 4.8 ± 0.6 | 4.9 ± 0.9 |
|  | S2 Novel object exploration (s) |  |  | 9.7 ± 1.6 | 12.8 ± 3.1 |
|  | Discrimination index (%) |  |  | 64.0 ± 3.8 | 68.5 ± 2.7 |
| Novel Location Recognition 3 hour delay | S1 Object A exploration (s) | 4.6 ± 0.6 | 5.5 ± 0.6 | NT | |
|  | S1 Object B exploration (s) | 4.3 ± 0.8 | 5.8 ± 0.7 |  |  |
|  | S2 Fixed object explo. (s) | 1.7 ± 0.2 | 2.5 ± 0.4 |  |  |
|  | S2 Moved object explo. (s) | 2.8 ± 0.5 | 2.9 ± 0.6 |  |  |
|  | Discrimination index (%) | 62.8 ± 2.9 | 51.8 ± 2.4 * |  |  |
| Social Interaction | Sniffing time (s) | 106 ± 9 | 76 ± 8 * | 147 ± 7 | 99.1 ± 18.9 * |
|  | Following time (s) | 16.7 ± 1.8 | 10.6 ± 3.6 | 23.8 ± 2.5 | 12.3 ± 4.3 * |
| Three-Chamber Sociability | S1 First animal exploration (s) | 167 ± 26 | 171 ± 21 | 155 ± 20 | 135 ± 13 |
|  | S2 Former animal exploration (s) | 35.4 ± 8.4 | 82.4 ± 10.3 ** | 64.2 ± 10.1 | 62.7 ± 5.5 |
|  | S2 Novel animal exploration (s) | 218 ± 51 | 113 ± 11 * | 86.6 ± 6.3 | 97.2 ± 11.4 |
|  | Discrimination index (%) | 81.3 ± 5.3 | 57.7 ± 4.0 ** | 59.2 ± 4.0 | 59.3 ± 3.9 |
| Rotarod | D1 Time on the rod (s) | 119 ± 7 | 113 ± 9 | 60.9 ± 14.4 | 61.4 ± 12.6 |
|  | D2 Time on the rod (s) | 119 ± 10 | 129 ± 9 | 76.7 ± 8.5 | 76.1 ± 10.7 |
|  | D3 Time on the rod (s) | 138 ± 12 | 140 ± 11 | 96.2 ± 10.1 | 101 ± 11 |
| Notched Bar | Hind limb errors (%) | 4.5 ± 0.8 | 5.2 ± 0.6 | 5.3 ± 0.6 | 5.6 ± 1.0 |
| Grip Test | Grip strength (g/g body weight) | 8.9 ± 0.5 | 10.7 ± 0.5 * | 8.5 ± 0.5 | 7.2 ± 0.4 * |

Working memory was first assessed with the Y maze test, which revealed an improvement in *Dup/+* animals. Recognition memory was first evaluated with the novel object recognition task. In the acquisition session (S1), no difference in object exploration was noticed between genotypes. After a 3-hour delay, *Del/+* and *Dup/+* mice show memory impairment and improvement for object identity, respectively. We did the same test for the *Dup/+* cohort with a 24-hour delay and found no phenotype, suggesting that duplication of Sult1a1–Spn impacts mnemonic processes associated with short-term memory. With the *Del/+* cohort, we performed the novel place recognition task with a 3-hour delay and also found a memory deficit for object location. In the social interaction test, we found a decrease of sniffing behavior for *Del/+* mice and a decrease of sniffing and following behaviors for *Dup/+* animals. In the three-chamber sociability test, we did not observe differences in social interaction, but novel animal discrimination was significantly decreased for *Del/+* animals. No motor coordination phenotype was seen in rotarod and notched bar tests but *Del/+* and *Dup/+* mice still presented stronger and weaker grip strength in the grip test, respectively. NT: not tested. Data are shown as the mean ± SEM. ^*^*P* < 0.05, ^**^*P* < 0.01 and ^***^*P* < 0.001, significantly different from wt counterparts, Student’s t-test.
