# Supplementary material for: Reciprocal Effects on Neurocognitive and Metabolic Phenotypes in Mouse Models of 16p11.2 Deletion and Duplication Syndromes
Source: PLoS Genet. 2016 Feb 12;12(2):e1005709. doi: 10.1371/journal.pgen.1005709 (PMC4752317; doi:10.1371/journal.pgen.1005709)
Supplement: S8 Table — Animals were put on a high-fat diet at the age of 5 weeks. In comparison with controls, Del/+ animals were underweight, shorter in size and deposited less fat whereas Dup/+ mice were overweight, longer in size and displayed more adipose tissue. Indirect calorimetry showed increased levels of energy expenditure (EE) and oxygen consumption (OC) for Del/+ mice during the dark phase. Dup/+ mice presented a diminution of EE and OC during dark and light phases. In intraperitoneal glucose tolerance tests (IPGTT), Del/+ mice showed faster glucose clearance whereas Dup/+ mice presented similar glucose clearance in comparison to controls but had an increased glycemia before injection of glucose (T0 blood-glucose). Blood chemistry analysis did not reveal gross hematology changes except a diminution of free fatty acids level of Del/+ mice. Finally, consistent with qNMR results, endocrinology analysis showed diminution of leptin and adiponectin level for Del/+ mice and an increase of leptin levels for Dup/+ mice. Data are shown as the mean ± SEM. *P < 0.05, **P < 0.01, ***P < 0.001, significantly different from wt counterparts, Student’s t-test. (DOCX) [file pgen.1005709.s016.docx]

**Supplementary Table S8.** High-fat diet analysis of *Del/+* and *Dup/+* cohorts on the C57BL/6N genetic background.

| Test | Parameter | B6N *Del/+* cohort results | | B6N *Dup/+* cohort results | |
| --- | --- | --- | --- | --- | --- |
|  |  | wt | Del/+ | wt | Dup/+ |
| Body Weight Evolution | 6-week weight | 15.6 ± 0.6 | 11.5 ± 0.1 *** | 17.3 ± 0.2 | 18.1 ± 0.4 |
|  | 10-week weight | 20.6 ± 0.6 | 16.6 ± 0.3 *** | 21.9 ± 0.3 | 24.2 ± 0.6 ** |
|  | 14-week weight | 23.5 ± 0.8 | 18.5 ± 0.5 *** | 25.6 ± 0.6 | 29.4 ± 1.2 * |
| Body size | snout/tail basis distance | 9.61 ± 0.05 | 9.01 ± 0.07 *** | 9.43 ± 0.04 | 9.84 ± 0.06 *** |
| qNMR | Fat body (%) | 17.4 ± 2.1 | 9.9 ± 0.5 ** | 17.9 ± 1.2 | 25.0 ± 1.9 ** |
|  | Lean body (%) | 72.8 ± 1.7 | 78.5 ± 0.7 ** | 73.1 ± 1.0 | 67.3 ± 1.6 * |
|  | Free body fluids (%) | 7.17 ± 0.80 | 8.54 ± 0.89 | 6.15 ± 0.26 | 5.52 ± 0.20 |
| Indirect Calorimetry (TSE) | Light EE (Kcal/kg^0.75/h) | 7.48 ± 0.27 | 7.86 ± 0.10 | 8.01 ± 0.21 | 7.04 ± 0.20 ** |
|  | Dark EE (Kcal/kg^0.75/h) | 8.87 ± 0.39 | 10.49 ± 0.62 * | 9.29 ± 0.28 | 8.11 ± 0.19 ** |
|  | Light OC (ml/kg^0.75/h) | 1355 ± 47 | 1429 ± 18 | 1458 ± 37 | 1283 ± 28 ** |
|  | Dark OC (ml/kg^0.75/h) | 1600 ± 70 | 1893 ± 111 * | 1678 ± 51 | 1468 ± 32 ** |
| Intraperitoneal Glucose tolerance test (IPGTT) | T_0_ blood glucose (mg/dl) | 94.0 ± 4.0 | 114 ± 8 | 79.0 ± 4.5 | 109 ± 8 * |
|  | T_60_ blood glucose (mg/dl) | 186 ± 6 | 154 ± 6 ** | 187 ± 10 | 203 ± 10 |
|  | T_120_ blood glucose (mg/dl) | 103 ± 3 | 95.0 ± 7.8 | 87.1 ± 4.1 | 106 ± 6 |
|  | AUC (min*mg/dl) | 13900 ± 855 | 8465 ± 739 *** | 13995 ± 718 | 13352 ± 1038 |
| Blood chemistry | Glucose (mmol/l) | 15.0 ± 0.5 | 15.5 ± 0.4 | 16.5 ± 0.6 | 16.4 ± 0.5 |
|  | T Cholesterol (mmol/l) | 2.40 ± 0.15 | 2.41 ± 0.09 | 2.51 ± 0.09 | 2.66 ± 0.17 |
|  | Triglycerides (mmol/l) | 0.57 ± 0.03 | 0.52 ± 0.02 | 0.61 ± 0.02 | 0.61 ± 0.04 |
|  | Free fatty acids (mEq/l) | 0.65 ± 0.02 | 0.57 ± 0.03 * | 0.54 ± 0.06 | 0.56 ± 0.02 |
|  | Glycerol (µmol/l) | 208 ± 10 | 193 ± 7 | 190 ± 16 | 212 ± 12 |
| Endocrinology | Insulin (µg/l) | 1.13 ± 0.35 | 1.40 ± 0.49 | 1.12 ± 0.28 | 2.56 ± 0.58 |
|  | Leptin (ng/ml) | 0.93 ± 0.22 | 0.32 ± 0.06 * | 1.38 ± 0.31 | 4.16 ± 0.97 * |
|  | Adiponectin (µg/l) | 13.5 ± 0.7 | 11.4 ± 0.4 * | 14.7 ± 0.5 | 15.5 ± 0.6 |

Animals were put on a high-fat diet at the age of 5 weeks. In comparison with controls, *Del/+* animals were underweight, shorter in size and deposited less fat whereas *Dup/+* mice were overweight, longer in size and displayed more adipose tissue. Indirect calorimetry showed increased levels of energy expenditure (EE) and oxygen consumption (OC) for *Del/+* mice during the dark phase. *Dup/+* mice presented a diminution of EE and OC during dark and light phases. In intraperitoneal glucose tolerance tests (IPGTT), *Del/+* mice showed faster glucose clearance whereas *Dup/+* mice presented similar glucose clearance in comparison to controls but had an increased glycemia before injection of glucose (T_0_ blood-glucose). Blood chemistry analysis did not reveal gross hematology changes except a diminution of free fatty acids level of *Del/+* mice. Finally, consistent with qNMR results, endocrinology analysis showed diminution of leptin and adiponectin level for *Del/+* mice and an increase of leptin levels for *Dup/+* mice. Data are shown as the mean ± SEM. ^*^*P* < 0.05, ^**^*P* < 0.01, ^***^*P* < 0.001, significantly different from wt counterparts, Student’s t-test.
