# Supplementary material for: Reciprocal Effects on Neurocognitive and Metabolic Phenotypes in Mouse Models of 16p11.2 Deletion and Duplication Syndromes
Source: PLoS Genet. 2016 Feb 12;12(2):e1005709. doi: 10.1371/journal.pgen.1005709 (PMC4752317; doi:10.1371/journal.pgen.1005709)
Supplement: S9 Table — Animals were put on a high-fat diet at the age of 5 weeks. Del/+ animals were underweight and displayed less fat than wild-type littermates. Indirect calorimetry showed an increase in the levels of energy expenditure (EE) and oxygen consumption (OC) for Del/+ mice during dark and light phases. No difference of energy excreted in feces was found in bomb calorimetric analyses. During intraperitoneal glucose tolerance tests (IPGTT), Del/+ mice showed faster glucose clearance but had an increased glycemia before injection of glucose (T0 blood-glucose). Blood chemistry analysis revealed an increased level of calcium and decreased levels of glucose and cholesterol for mutant mice. Finally, in consistent with qNMR results, endocrinology analysis showed diminution of leptin and adiponectin level for Del/+ mice. Data are shown as the mean ± SEM. *P < 0.05, **P < 0.01, ***P < 0.001, significantly different from wt counterparts, Student’s t-test. (DOCX) [file pgen.1005709.s017.docx]

**Supplementary Table S9.** High-fat diet analysis of *Del/+* cohort on the C57BL/6N-C3B genetic background.

|  |  |  |  | |
| --- | --- | --- | --- | --- |
| Test | Parameter | B6N *Del/+* cohort results | | |
|  |  | wt | | Del/+ |
| Body Weight Evolution | 6-week weight | 19.2 ± 0.4 | | 16.6 ± 0.3 *** |
|  | 10-week weight | 22.4 ± 0.3 | | 19.1 ± 0.3 *** |
|  | 14-week weight | 25.2 ± 0.3 | | 22.0 ± 0.4 *** |
| qNMR | Fat body (%) | 20.4 ± 1.3 | | 14.1 ± 0.6 *** |
|  | Lean body (%) | 71.7 ± 1.4 | | 76.5 ± 0.5 ** |
|  | Free body fluids (%) | 5.99 ± 0.28 | | 7.23 ± 0.30 ** |
| Indirect Calorimetry (TSE) | Light EE (Kcal/kg^0.75/h) | 8.66 ± 0.23 | | 9.96 ± 0.25 ** |
|  | Dark EE (Kcal/kg^0.75/h) | 9.66 ± 0.22 | | 11.45 ± 0.41 ** |
|  | Light OC (ml/kg^0.75/h) | 1570 ± 40 | | 1800 ± 45 ** |
|  | Dark OC (ml/kg^0.75/h) | 1311 ± 33 | | 1584 ± 57 ** |
| Calorimetric Bomb | Faeces energy content (cal/g faeces) | 3835 ± 46 | | 3782 ± 70 |
|  | Energy excreted (Kcal/mouse/day) | 3.07 ± 0.10 | | 2.78 ± 0.20 |
| Intraperitoneal Glucose tolerance test (IPGTT) | T_0_ blood glucose (mg/dl) | 62.7 ± 4.0 | | 91.4 ± 4.7 *** |
|  | T_60_ blood glucose (mg/dl) | 232 ± 9 | | 176 ± 21 * |
|  | T_120_ blood glucose (mg/dl) | 72.8 ± 1.6 | | 82.1 ± 4.3 |
|  | AUC (min*mg/dl) | 17778 ± 519 | | 10334 ± 1908 ** |
| Blood chemistry | Glucose (mmol/l) | 16.0 ± 0.4 | | 14.2 ± 0.6 * |
|  | T Cholesterol (mmol/l) | 3.21 ± 0.07 | | 2.86 ± 0.10 * |
|  | Triglycerides (mmol/l) | 0.86 ± 0.10 | | 0.95 ± 0.09 |
|  | Free fatty acids (mEq/l) | 0.95 ± 0.07 | | 0.95 ± 0.03 |
|  | Glycerol (µmol/l) | 286 ± 14 | | 281 ± 8 |
|  | Ca (mmol/l) | 2.29 ± 0.01 | | 2.35 ± 0.01*** |
|  | P (mmol/l) | 1.93 ± 0.08 | | 1.86 ± 0.50 |
| Endocrinology | Leptin (ng/ml) | < 0.1 | | < 0.1 |
|  | Leptin (ng/ml) | 39.0 ± 3.4 | | 10.7 ± 2.5 *** |
|  | Adiponectin (µg/l) | 7.80 ± 0.18 | | 6.34 ± 0.42 ** |

Animals were put on a high-fat diet at the age of 5 weeks. *Del/+* animals were underweight and displayed less fat than wild-type littermates. Indirect calorimetry showed an increase in the levels of energy expenditure (EE) and oxygen consumption (OC) for *Del/+* mice during dark and light phases. No difference of energy excreted in feces was found in bomb calorimetric analyses. During intraperitoneal glucose tolerance tests (IPGTT), *Del/+* mice showed faster glucose clearance but had an increased glycemia before injection of glucose (T_0_ blood-glucose). Blood chemistry analysis revealed an increased level of calcium and decreased levels of glucose and cholesterol for mutant mice. Finally, in consistent with qNMR results, endocrinology analysis showed diminution of leptin and adiponectin level for *Del/+* mice. Data are shown as the mean ± SEM. ^*^*P* < 0.05, ^**^*P* < 0.01, ^***^*P* < 0.001, significantly different from wt counterparts, Student’s t-test.
