# Supplementary material for: Reciprocal Effects on Neurocognitive and Metabolic Phenotypes in Mouse Models of 16p11.2 Deletion and Duplication Syndromes
Source: PLoS Genet. 2016 Feb 12;12(2):e1005709. doi: 10.1371/journal.pgen.1005709 (PMC4752317; doi:10.1371/journal.pgen.1005709)
Supplement: S10 Table — Our analysis considers the pre-ranked gene list according to the LIMMA results based on the dosage model. The gene set database used was the MSigDB C2 (c2.all.v4.0.symbols.gmt), which collects curated gene sets from online pathways, publications in PubMed, and the knowledge of domain experts. The table include gene sets dose-dependently upregulated (upregulated in the Dup/+ mice and downregulated in Del/+ mice) and downregulated (downregulated in the Dup/+ mice and upregulated in Del/+ mice). Data with an FDR below 25% are shown. CEREB: Cerebellum; DEREG: Deregulation; HIP: Hippocampus STRI: Striatum. (DOCX) [file pgen.1005709.s018.docx]

**Supplementary Table S10.** GSEA analysis

Our GSEA analysis considers the pre-ranked gene list according to the LIMMA results based on the dosage model. The gene set database used was the MSigDB C2 (c2.all.v4.0.symbols.gmt), which collects curated gene sets from online pathways, publications in PubMed, and the knowledge of domain experts. The table include gene sets dose-dependently upregulated (upregulated in the *Dup/+* mice and downregulated in *Del/+* mice) and downregulated (downregulated in the *Dup/+* mice and upregulated in *Del/+* mice). Data with an FDR below 25% are shown. CEREB: Cerebellum; DEREG: Deregulation; HIP: Hippocampus STRI: Striatum.
