# Supplementary material for: Reciprocal Effects on Neurocognitive and Metabolic Phenotypes in Mouse Models of 16p11.2 Deletion and Duplication Syndromes
Source: PLoS Genet. 2016 Feb 12;12(2):e1005709. doi: 10.1371/journal.pgen.1005709 (PMC4752317; doi:10.1371/journal.pgen.1005709)
Supplement: S1 File — Detailed protocols used in the study or for supplementary figures and tables are provided in the document. (DOCX) [file pgen.1005709.s019.docx]

# Supplementary information

**Generation of mice carrying rearrangements of the *Sult1a1–Spn* genetic interval**

To generate mouse models of the 16p11.2 BP4-BP5 rearrangements, we introduced LoxP sites flanking the syntenic region located on chromosome 7F3 **(**Supp Fig. 1A). Mutant mice were obtained by using an *in vivo* TAMERE strategy [1, 2]. LoxP sites were first introduced by homologous recombination in C57BL/6N (B6N) embryonic stem cells at *Sult1a1* and *Spn* loci in the same orientation and the corresponding mouse line were generated. Selection cassettes were excised and mice were crossed with Hprt<tm1(cre)Mnn> mice [3], expressing the Cre recombinase under the control of the X-linked hypoxanthine guanine phosphoribosyl transferase gene promoter active in oocytes. Females born from this mating and bearing both the Hprt<tm1(cre)Mnn> transgene and loxP sites in a trans configuration were mated with wt B6N males (Supp Fig. 1B). We recovered mice carrying the deletion (*Del/+*) and the duplication (*Dup/+*) for the *Sult1a1-Spn* region with a recombination frequency of respectively 3 and 4 animals out of 97 newborns.

**Behavioral analysis**

Anxiety and activity of mice were evaluated with the elevated plus maze. The apparatus consists of two opposed open arms (30 x 5 cm) crossed by two enclosed arms (30 x 5 x 15 cm), and elevated 66 cm from the floor. The light intensity at the extremity of the open arms was kept at 50 Lux. Each mouse was tested for 5 min after being placed in the central platform and allowed to explore freely the apparatus. The number of entries and time spent in the open arms were used as an anxiety index. Closed arm entries and rears in the closed arms were used as measures of general motor activity.

The Morris water maze paradigm was used to evaluate spatial learning and memory of mice. Our protocol is adapted from an established protocol [4]. The apparatus consists in a circular pool (150-cm diameter, 60-cm height) filled to a depth of 40 cm with water maintained at 20°C–22°C and made opaque using a white aqueous emulsion (Acusol OP 301 opacifier). An escape platform, made of 6 cm diameter rough plastic, is submerged 1 cm below the water surface. The test began with 6 days of acquisition, 4 trials per day, at 120 Lux. Each trial started with the mouse facing the interior wall of the pool and ended when the animal either climbs on the platform or after a maximum searching time of 90 sec. The platform was at the same position for all four trials but starting positions changed randomly between each trial with departures from each cardinal point. Distances travelled to find the platform and swimming speeds were analysed each day. On the 7^th^ day, mice were given a single trial of 60 seconds during the probe test or removal session in which the platform was removed. The distance travelled and duration spent in each quadrant (NW, NE, SW, SE) were recorded. Annulus crossing index was calculated as the number of times that animals crossed the exact platform position. On 12^th^ and 13^th^ days, mice were tested with reversal sessions with 4 trials of 90 seconds per day. The platform was made visible by a small dark ball placed 12cm on top of the platform, while the external cues were hidden by surrounding the pool with a black curtain. To be sure that the mouse used the platform cue, starting position and platform position were changed for each trial.

In addition to the new object recognition (NOR), we used the object location recognition task (NLR) to evaluate the recognition memory of animals. In the first 10-min acquisition trial, animals were exposed to two objects, a green marble and a die. 3 hours later, one of the familiar objects was displaced to a novel location (object B) and the exploration time of the two objects was recorded for 10 min. A discrimination index was defined as (*t*_B_/(*t*_A_ + *t*_B_)) x 100. All mice which did not explore both objects more than 4 seconds during the acquisition trial were excluded from the analysis.

The Y-maze test was used to evaluate short-term working memory. This test is based on the innate preference of animals to explore an arm that has not been previously explored, a behavior that, if occurring with a frequency greater than 50%, is called spontaneous alternation. In this test, we used a Y-shaped maze with three white, opaque plexiglass arms of equivalent length forming a 120° angle with each other. The arms have walls with specific motifs to distinguish from each other. After introduction at the center of the maze illuminated at 60 Lux, animals were allowed to explore freely the three arms for 6 minutes. The number of arm entries and the number of triads were recorded to calculate the percentage of alternation.

The marble burying test was used to evaluate anxiety and impulsive/compulsive behaviors of animals. Mice were put individually into dimly lit (60 Lux) clean home-cages containing a 6-cm layer of sawdust covered with 24 colorless marbles of 1-cm diameter. Numbers of marbles partially and fully buried are recorded after 15-min of test.

In addition to the social interaction test, social behavior was tested using the three-chamber sociability test for social preference and discrimination using a specific apparatus (Stoelting, Dublin) with three successive and identical chambers (20 cm × 40 cm × 22 (height) cm with 5 cm × 8 cm openings allowing access between the chambers). The testing wad performed as previously described [5]. During habituation, mice were allowed to explore the three chambers freely for 10 min. In the second phase, the test mouse was placed into the central box, while an unfamiliar mouse (stranger 1) was put into one of the wire cages in a random and balanced manner. The doors were reopened and the test mouse was allowed to explore the chambers for 10 min. Time spent to explore the empty cage and the cage with stranger 1 was recorded. In the third phase, the social discrimination was evaluated with a new mouse being placed into the empty wire cage and the test mouse was allowed to explore again the entire arena for 10 min, having the choice between the familiar mouse (stranger 1 or 1) and the novel mouse (stranger 2 or 2). A social discrimination index or social preference was defined as the percentage of time spent exploring the novel mouse as (*t*_2_/(*t*_1_ + *t*_2_)) × 100.

A forced swimming test was used to detect a depressed state by forcing mice to swim in a narrow cylinder from which they cannot escape. Mice were placed in a Plexiglass cylinder containing water (21°C-23°C) at a depth of 15 cm. After a brief period of vigorous activity, mice adopt a characteristic floating posture. Each animal was submitted to a forced swim session of 6 min and the total duration of floating and swimming behaviors were measured.

Acoustic startle reflex was evaluated with the prepulse inhibition (PPI) test using eight startle devices (SRLAB, San Diego Instruments, San Diego, CA), each consisting of a Plexiglas cylinder (5.1 cm outside diameter) mounted on a Plexiglas platform in a ventilated, sound-attenuated cubicle with a high-frequency loudspeaker (28 cm above the cylinder) producing both a continuous background noise and various acoustic stimuli. The background noise of each chamber was set at 65 dB. Movements within the cylinder were detected and transduced by a piezoelectric accelerometer attached to the Plexiglass base, digitized, and stored by a computer. Beginning at the stimulus onset, 65 readings of 1ms were recorded to obtain the animal’s startle amplitude. Auditory stimuli are bursts of white noise (0–20KHz and 0 ms rise–decay). The optimal PPI parameters (eg, stimuli intensity and duration, prepulse–pulse intervals) were defined based on our previous validation studies [6]. Stimuli levels and piezoaccelerometer sensitivity were calibrated before each PPI session. Testing is adapted from the established protocol [7]. The session was initiated with 5 min acclimation period followed by 10 different trial types: acoustic startle pulse alone (white noise, 110 dB/40 ms); 8 different prepulse trials in which either 20 ms long 70, 80, 85 or 90 dB stimuli were presented alone or preceded the pulse by 50 ms, and finally 1 trial in which only the background noise was presented to measure the baseline movement in the Plexiglass cylinder. The test session begun with five presentations of the startle pulse trial, which were excluded from statistical analysis. Then, each acoustic or BN trial was presented 10 times in random order. The average ITI was 15 s (10–20 s).

The pentylenetetrazol (PTZ) sensitivity test was used to determine the seizure threshold of animals. PTZ acts as a non-competitive GABA-A receptor antagonist, reducing neuronal inhibition [8]. This substance induces myoclonic, clonic or tonic seizures depending on the dose injected [9]. PTZ solution was freshly prepared (30 mg in 10 ml of 0.9% NaCl) and injected intraperitoneally at 30 mg/kl (300µL of solution for a 30g mouse). Immediately after PTZ adimistration, mice were put in a clean translucent arena. Events of myoclonic seizures were noted. If mice had clonic seizure, the latency after PTZ injection and the duration of event was noted.

Hedonic behavior was studied in the sucrose preference test by measuring preference for sucrose when the animal has free access to two bottles containing either a 0.8% sucrose solution or water. Mice were first habituated to sucrose in their home cages where water was replaced with sucrose solution (from 5 pm on day 1 to 9 am on day 2). On days 2–4, mice were evaluated for sucrose preference in testing cages (30 × 15 × 12 cm^3^). At 5 pm of each day, mice were put into individual cages with food *ad libitum*. One hour later, two bottles of water and 0.8% sucrose were provided for 15 h. At 9 am on the next day, mice were put back to their home cage and the two bottles were weighed.

The muscular strength was evaluated with the grip strength. Mice were first weighed and tested with a handy force gauge (Bioseb, France). Animals were placed on the instrument grid and pulled by the tail until letting go. The force (g) was related to animal weight (g).

The social odour discrimination test was undertaken to verify olfaction and to validate sociability results based principally on this sense. The test was adapted from a described procedure[10]. Mice were assessed for water versus social odor discrimination. Odor exploration was measured in a clean cage with fresh bedding. Odors were presented on two cotton-tipped wooden applicators. Animals were first habituated to the novel cage for 3 min, and then tested during 3 consecutive sessions of 3 min each with 2 min inter-trial interval (ITI). During each session, two applicators were presented: (session 1) water-water, (session 2) water-social odour, and (session 3) water-social odour. The position of the odour for the sessions 2 and 3 was randomized. Cotton sniffing durations (when the animal’s snout was directed toward the cotton at a distance ≤1 cm) were recorded for each session.

The non-social odor discrimination was adapted from an established protocol [11]. Odor exploration was measured in a clean cage with fresh bedding. Odors were presented on a small piece of Whatman paper placed in a perforated tube (H: 4 cm, diameter: 3 cm). Animals were first habituated to the novel cage for 3 min, and then tested during 5 consecutive sessions of 2 min, each with 8 min inter-trial interval (ITI). In the 4 first sessions, the perforated tube contained Whatman paper soaked with orange flower water. On the last session, Whatman paper was replaced with the paper soaked with vanilla extract. Odor sniffing duration (when the animal’s snout was directed toward the perforated tube at a distance ≤1 cm) was recorded for each session.

#### Auditory brain response (ABR) recordings

The auditory brainstem response (ABR) helped us to determine hearing sensitivity [12]. Four *Del/+*, 4 wt, 4 *Del/Dup*, and 2 *Dup/+* animals were anesthetized using intraperitoneal injection of ketamine/xylazine and placed on a heating blanket inside a sound attenuating booth. Sub-cutaneous needle electrodes were inserted in the skin on the vertex (active) and overlying the ventral region of the left (reference) and right (ground) bullae to record responses of the left ear. Stimuli were presented as free-field sounds from a speaker (Tucker Davis Technologies, FF1) whose leading edge was 10 cm in front of the mouse's interaural axis at an elevation of 30°. The sound delivery system was calibrated using an ACO Pacific 7017 microphone. For threshold determination, custom software, and Tucker Davis Technologies hardware were used to deliver click (0.01 ms duration) and tone pip (6, 12, 18, 24 and 30 kHz of 5 ms duration, 1 ms rise/fall time) stimuli at a range of intensity levels from 10–100+ dB SPL in 3 dB steps. Averaged responses to 256 stimuli, presented at 42.2/s, were analyzed and thresholds established as the lowest sound intensity giving a visually-detectable ABR response. For clicks, responses were also recorded from the right ear.

#### Hippocampal slice electrophysiology

Acute hippocampal slices were used to record field excitatory post synaptic potentials (fEPSPs), by the MEA60 electrophysiological suite (Multi Channel Systems, Reutlingen, FRG) as described [13, 14]. Eight set-ups consisting of a MEA1060-BC pre-amplifier and a filter amplifier (gain 550x) were run simultaneously by data acquisition units operated by MC_Rack software. Raw electrode data were digitized at 10 kHz and stored on a PC hard disk for subsequent analysis. To record fEPSPs, a hippocampal slice was placed into the well of the 5x13 3D multi electrode array (MEA) biochip (Qwane Biosciences, Lausanne, Switzerland). The slice was guided to a desired position with a fine paint brush and gently fixed over MEA electrodes by a silver ring with attached nylon mesh lowered vertically by a one-dimensional U-1C micromanipulator (You Ltd, Tokyo, Japan). MEA biochips were fitted into the pre-amplifier case and fresh ACSF was delivered to the MEA well through a temperature-controlled perfusion cannula that warmed perfused media to 32°C. Monopolar stimulation of Schäffer collateral /commissural fibers through array electrodes was performed by STG4008 stimulus generator (Multi Channel Systems, Reutlingen, FRG). Biphasic (positive/negative, 100 µs/a phase) voltage pulses were used. Amplitude, duration and frequency of stimulation were controlled by MC_Stimulus II software. All experiments were performed using two-pathway stimulation of Schäffer collateral/commissural fibers. Our previous experiments that utilized MEAs, demonstrated that largest LTP was recorded in proximal part of apical dendrites of CA1 pyramidal neurons [13]. We have therefore picked a single principal recording electrode in the middle of the proximal part of the CA1 region and assigned two electrodes for stimulation of the control and test pathways on the subicular side and on the CA3 side of *stratum radiatum* respectively. The distance from the recording electrode to the test stimulation electrode was 400-510 µm and to the control stimulation electrode 316-447 µm. To evoke orthodromic fEPSPs, test and control pathways were activated in succession at a frequency of 0.02 Hz. Baseline stimulation strength was adjusted to evoke a response that corresponded to 40% of the maximal attainable fEPSP at the recording electrode located in proximal *stratum radiatum*. Slope of the negative part of fEPSPs was used as a measure of the synaptic strength. Paired stimulation with an interpulse interval of 50 ms was used to observe paired-pulse facilitation (PPF) in baseline conditions in the test pathway before LTP induction. PPF was calculated by dividing the negative slope of fEPSP obtained in response to the second pulse by the amplitude of fEPSP amplitude evoked by the preceding pulse. To induce LTP, 10 bursts of baseline strength stimuli were administered at 5 Hz to test pathway with 4 pulses given at 100 Hz per burst (total 40 stimuli). LTP plots were scaled to the average of the first five baseline points. Normalization of LTP values was performed by dividing the fEPSP amplitude in the tetanized pathway by the amplitude of the control fEPSP at corresponding time points. Normalized LTP values averaged across the period of 61-65 min after theta-burst stimulation were used for statistical comparison.

**Craniofacial analysis**

Three-dimensional coordinates of 39 relevant cranial landmarks were recorded using Landmark software, and posterior comparisons were performed using the Euclidean distance matrix analysis (EDMA) with the WinEDMA software (version 1.0.1 beta). Three-dimensional data were converted into linear distances compiled into a matrix. Both the form (size of the skull) difference matrix (FDM) and the shape difference matrix (SDM) were analysed. A ratio different from 1 (FDM) or 0 (SDM) for any linear distance indicates that the two samples are not similar for that measure.

Confidence intervals were estimated using a non-parametric bootstrapping algorithm. For each linear distance, the null hypothesis was rejected if the 90% confidence interval did not include 1 (FDM) or 0 (SDM): rejection of the null hypothesis enabled localization of differences to specific landmarks and linear distances. Bootstrap Distributions of T (FDM) and Z (SDM) were calculated as follows: for each FDM, a T value was calculated.

**Tibialis anterior muscle contractile properties and succinate dehydrogenase staining**

Tibialis anterior (TA) of 28 week-old male mice (*n* = 6 wt; n = 5 *Del/+; n* = 7 *Del/Dup*; n = 8 *Dup/+*) of C57BL/6N genetic background were used for the study. Muscle force measurements were evaluated by measuring in situ muscle isometric contraction in response to nerve stimulation, as described previously [15]. Results from nerve stimulation are shown on fig S4. Fatigue was measured as time taken to reach 50% of the maximum force produced. After contractile measurements, the animals were killed by cervical dislocation. TA muscles were then dissected and weighed to determine specific maximal force. Transverse cryosections (8 μm) of mouse skeletal muscles were prepared, fixed, and stained with succinate dehydrogenase (SDH)[16]. Sections were imaged with a slide scanner NanoZoomer 2HT.

**REFERENCES**

1. Herault Y, Rassoulzadegan M, Cuzin F, Duboule D. Engineering chromosomes in mice through targeted meiotic recombination (TAMERE). Nature Genetics. 1998;20(4):381-4. PubMed PMID: WOS:000077199600028.

2. Brault V, Pereira P, Duchon A, Herault Y. Modeling chromosomes in mouse to explore the function of genes, genomic disorders, and chromosomal organization. Plos Genetics. 2006;2(7):911-9. doi: e86

10.1371/journal.pgen.0020086. PubMed PMID: WOS:000239494800001.

3. Tang SHE, Silva FJ, Tsark WMK, Mann JR. A Cre/loxP-deleter transgenic line in mouse strain 129S1/SvImJ. Genesis. 2002;32(3):199-202. doi: 10.1002/gene.10030. PubMed PMID: WOS:000174538100002.

4. Duchon A, Pothion S, Brault V, Sharp AJ, Tybulewicz VLJ, Fisher EMC, et al. The telomeric part of the human chromosome 21 from Cstb to Prmt2 is not necessary for the locomotor and short-term memory deficits observed in the Tc1 mouse model of Down syndrome. Behavioural Brain Research. 2011;217(2):271-81. doi: 10.1016/j.bbr.2010.10.023. PubMed PMID: WOS:000286698300003.

5. Moy SS, Nadler JJ, Perez A, Barbaro RP, Johns JM, Magnuson TR, et al. Sociability and preference for social novelty in five inbred strains: an approach to assess autistic-like behavior in mice. Genes Brain Behav. 2004;3(5):287-302. doi: 10.1111/j.1601-1848.2004.00076.x. PubMed PMID: 15344922.

6. Aubert L, Reiss D, Ouagazzal AM. Auditory and visual prepulse inhibition in mice: parametric analysis and strain comparisons. Genes Brain Behav. 2006;5(5):423-31. doi: 10.1111/j.1601-183X.2005.00178.x. PubMed PMID: 16879636.

7. Ouagazzal AM, Reiss D, Romand R. Effects of age-related hearing loss on startle reflex and prepulse inhibition in mice on pure and mixed C57BL and 129 genetic background. Behav Brain Res. 2006;172(2):307-15. doi: 10.1016/j.bbr.2006.05.018. PubMed PMID: 16814879.

8. Ramanjaneyulu R, Ticku MK. Interactions of pentamethylenetetrazole and tetrazole analogues with the picrotoxinin site of the benzodiazepine-GABA receptor-ionophore complex. Eur J Pharmacol. 1984;98(3-4):337-45. PubMed PMID: 6327331.

9. Meldrum B. Do preclinical seizure models preselect certain adverse effects of antiepileptic drugs. Epilepsy Res. 2002;50(1-2):33-40. PubMed PMID: 12151115.

10. Yang M, Crawley JN. Simple behavioral assessment of mouse olfaction. Curr Protoc Neurosci. 2009;Chapter 8:Unit 8.24. doi: 10.1002/0471142301.ns0824s48. PubMed PMID: 19575474; PubMed Central PMCID: PMCPMC2753229.

11. Ferguson JN, Young LJ, Hearn EF, Matzuk MM, Insel TR, Winslow JT. Social amnesia in mice lacking the oxytocin gene. Nat Genet. 2000;25(3):284-8. doi: 10.1038/77040. PubMed PMID: 10888874.

12. Kuhn S, Ingham N, Pearson S, Gribble SM, Clayton S, Steel KP, et al. Auditory function in the Tc1 mouse model of down syndrome suggests a limited region of human chromosome 21 involved in otitis media. PLoS One. 2012;7(2):e31433. doi: 10.1371/journal.pone.0031433. PubMed PMID: 22348087; PubMed Central PMCID: PMCPMC3279367.

13. Kopanitsa MV, Afinowi NO, Grant SG. Recording long-term potentiation of synaptic transmission by three-dimensional multi-electrode arrays. BMC Neurosci. 2006;7:61. doi: 10.1186/1471-2202-7-61. PubMed PMID: 16942609; PubMed Central PMCID: PMCPMC1574331.

14. Coba MP, Komiyama NH, Nithianantharajah J, Kopanitsa MV, Indersmitten T, Skene NG, et al. TNiK is required for postsynaptic and nuclear signaling pathways and cognitive function. J Neurosci. 2012;32(40):13987-99. doi: 10.1523/JNEUROSCI.2433-12.2012. PubMed PMID: 23035106; PubMed Central PMCID: PMCPMC3978779.

15. Cowling BS, Toussaint A, Amoasii L, Koebel P, Ferry A, Davignon L, et al. Increased expression of wild-type or a centronuclear myopathy mutant of dynamin 2 in skeletal muscle of adult mice leads to structural defects and muscle weakness. Am J Pathol. 2011;178(5):2224-35. doi: 10.1016/j.ajpath.2011.01.054. PubMed PMID: 21514436; PubMed Central PMCID: PMCPMC3081151.

16. Brault V, Duchon A, Romestaing C, Sahun I, Pothion S, Karout M, et al. Opposite phenotypes of muscle strength and locomotor function in mouse models of partial trisomy and monosomy 21 for the proximal Hspa13-App region. PLoS Genet. 2015;11(3):e1005062. doi: 10.1371/journal.pgen.1005062. PubMed PMID: 25803843; PubMed Central PMCID: PMCPMC4372517.
